# Supplementary figures and images for: Appraising the quality standard of clinical practice guidelines related to central venous catheter-related thrombosis prevention: a systematic review of clinical practice guidelines
Source: BMJ Open. 2024 Mar 11;14(3):e074854. doi: 10.1136/bmjopen-2023-074854 (PMC10936513; doi:10.1136/bmjopen-2023-074854)

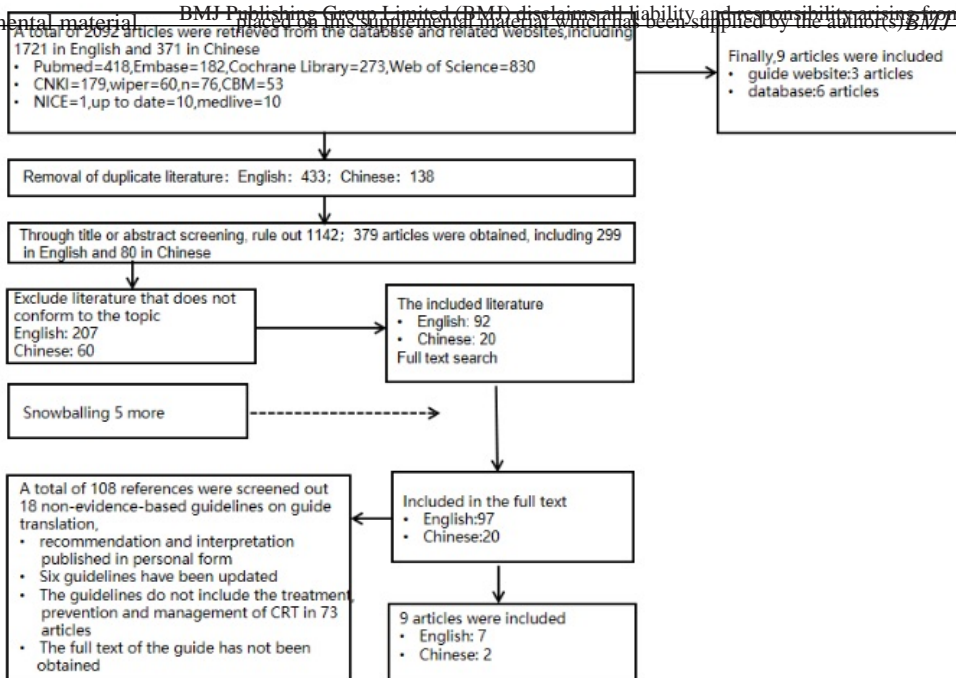

Supplement: Supplementary data [file bmjopen-2023-074854supp002.pdf]
