# Supplementary material for: Appraising the quality standard of clinical practice guidelines related to central venous catheter-related thrombosis prevention: a systematic review of clinical practice guidelines
Source: BMJ Open. 2024 Mar 11;14(3):e074854. doi: 10.1136/bmjopen-2023-074854 (PMC10936513; doi:10.1136/bmjopen-2023-074854)
Supplement: Supplementary data [file bmjopen-2023-074854supp003.pdf]

Table S1. Includes the basic characteristics table of the guideline

Number	Name	Year	Publishing agency	Subject	language	Whether or not evidence-based guidelines	Number of references
1	Chinese Expert Consensus on the Prevention and Treatment of Infusion Catheter-related Venous Thrombosis (2020 Edition)	2020	Vascular Alliance China Chapter	Catheter-related thrombosis prevention	Chinese	Not	62
2	Venous Thromboembolism Prophylaxis and Treatment in Patients With Cancer: ASCO Clinical Practice Guideline Update	2019	American Society of Clinical Oncology	Prevention of cancer-related blood clots	English	Yes	155
3	Guidelines for the prevention and treatment of thrombotic diseases in China	2018	Chinese Medical Association	Diagnosis and prevention of thrombosis	Chinese	Not	41
4	SEOM clinical guideline of venous thromboembolism (VTE) and cancer	2019	Spanish Society of Medical Oncology	Prevention and treatment of cancer-related thrombosis	English	Yes	88
5	Cancer Associated Venous Thromboembolic Disease	2021	National Comprehensive Cancer Network	Strategies for the assessment, prevention, and management of cancer-related thrombosis	English	Yes	222

6	American Society of Hematology 2020 guidelines for management of venous thromboembolism: optimal management of anticoagulation therapy treatment and prophylaxis of venous thromboembolism in patients with cancer	2020	American Society of Hematology	Drug prophylaxis strategies for venous thrombosis	English	Yes	360
7	2019 international clinical practice guidelines for thetreatment and prophylaxis of venous thromboembolism in patients with cancer treatment and prophylaxis of venous thromboembolism in patients with cancer	2019	International Thrombosis and Cancer Initiative	Prevention strategies for cancer-related thrombosis	English	Yes	94
8	Venous thromboembolism in over 16s: reducing the risk of hospital-acquired deep vein thrombosis or pulmonary embolism	2018	National Institute for Health and Care Excellence, UK	Strategies for the prevention and treatment of blood clots	English	Not	330
9	Infusion Therapy Standards of Practice	2021	Infusion Nurses Association	Methods and strategies of clinical infusion therapy	English	Not	471

Note: American Society of Clinical Oncology (ASCO); Spanish Society of Medical Oncology (SEMO); National Comprehensive Cancer Network (NCCN); American Society Hematological(ASO); International Initiative on Thrombosis and Cancer(ITAC-CME); National Institute for Health and Clinical Excellence(NICE); Infusion Nursing(INS).

Zhang J, *et al. BMJ Open* 2024; 14:e074854. doi: 10.1136/bmjopen-2023-074854

Table S2 Specific standardized scores for each AGREE II domain

number	Standardized score by domain (%)						The number of standardized scores ≧ 60%.	recommend level
	range and objective	Participants	Rigour	Clarity	Application	independence		
1	62.96	31.48	31.94	64.81	31.94	44.44	2	B
2	75.93	72.22	63.89	68.52	66.67	75.00	6	A
3	74.07	81.48	77.08	55.56	72.22	69.44	5	B
4	38.89	33.33	30.56	61.11	41.67	61.11	2	B
5	70.37	68.52	66.67	62.96	47.22	61.11	5	B
6	79.63	83.33	84.03	77.78	80.56	72.22	6	A
7	64.81	62.96	64.58	61.11	66.67	52.78	5	B
8	72.22	77.78	72.92	72.22	69.44	72.22	6	A
9	61.11	35.19	65.28	59.26	68.06	63.89	4	B
Mean Score	66.67	60.70	61.88	64.81	60.49	63.58	-	-

Table S3 Specific standardized scores of AGREE REX in each field

number	AGREE REX Score by Sector (%)			Overall rating
	Clinical applicability	Values and preferences	Implementability	
1	42.59	41.67	50.00	43.83
2	61.11	61.11	66.67	62.35
3	74.07	65.28	77.78	70.99
4	37.04	41.67	61.11	44.44
5	50.00	61.11	72.22	59.88
6	83.33	73.61	77.78	77.78
7	59.26	50.00	63.89	56.17
8	66.67	66.67	77.78	69.14
9	48.15	59.72	52.78	54.32

Table S4. Summary of recommendations included in the guidelines

Items		Source guideline	Recommendation
Risk screening	Thrombosis assessment tool	1、2、3、5、7、8、9	1. The main risk factors assessed included catheterization factors (catheter materials, placement techniques, location and type of catheter, presence of other catheters or catheters, etc.); potential co-disease factors (antiangiogenic therapy, hormone therapy, parenteral nutrition and radiotherapy, tumor and trauma and complex surgery) Patient factors (age, gender, family history or medical history of venous thrombosis, hypercoagulation status and abnormal coagulation gene).<br>2. At present, there is no scale dedicated to CRT risk assessment, and it is recommended to use mature VTE risk assessment scale in clinical practice. Modified Khorana score and Wells scale should be used in CRT risk assessment. Padua scale is recommended for medical patients and IMPED and SAVED scales are recommended for patients with multiple myeloma.
	Timing of thrombo	1、2、3、5、8、9	3. Admission / pre-catheterization / maintenance catheter / symptoms and signs / catheter extraction / discharge / anticoagulant therapy.

	sis evaluation		
Preventions	Identify as soon as possible	1 、 3 、 9	4. Pay close attention to CRT related symptoms and signs such as pain, swelling and skin pruritus on the side of catheterization, and monitor blood routine and coagulation function in time.
	Stay alert.	2 、4	5. Correct the wrong operation in time through quality control, conduct regular staff meetings to summarize and feedback on the recent work, and formulate staff briefing warnings to remind other personnel.
	Timely report	1 、 2 、 4 、 8	6. Pay attention to high-risk patients and timely report adverse reactions to prevent them.
	Classified prevention	1 、 2 、 3 、 5 、 8	7. To evaluate the risk factors and existing symptoms and signs of CRT in patients, and target to prevent and manage them according to different factors and clinical manifestations.<br>8. According to the risk benefit model, individualized drug prevention and physical prevention.
	Multi-disciplinary preventi	1 、 2 、 4 、 5 、 6 、 9	9. To construct a venous access management team composed of doctors, nurses, pharmacists, medical technologists, evidence-based medicine experts and managers to improve the scientific and comprehensive of CRT prevention and

	on		treatment.
	Physical prevention	1、2、5、8、9	10.Patients who stay in bed for a long time or have limited activity can massage their lower extremities with intermittent inflatable pressure pump to prevent thrombosis for 1 to 2 weeks. The skin condition of the patients should be evaluated before and after use.<br>11.To carry out daily activities as soon as possible, CVC catheter side should carry out early functional exercise, such as repeatedly clenching , loose, chest breathing and abdominal breathing, etc.<br>12.Replenish the patient with water in time to relieve dehydration.<br>13.For patients at risk of post-thrombosis syndrome, elastic socks are not recommended.
Preventions	Drug prevention	2、4、5、6、8	14.After evaluating the risk of bleeding and thrombosis, unfractionated heparin, low molecular heparin, vitamin K antagonist or direct oral anticoagulant drug can be used for prevention. After taking the drug, the laboratory indexes of the patients should be monitored, INR should be monitored in patients with Warfarin, and the arm circumference of the catheterization side should be measured regularly to ask if the patient has bleeding related manifestations.

Common catheter-related thrombosis prevention	1、2、3、4、5、6、7、8、9	15. When CVC intubation, the catheter with small diameter, less lumen and less trauma should be selected. If conditions permit, the ratio of the outer diameter of the catheter to the diameter of the catheterized vein should be less than 45%.<br>16. Ultrasonic guided intubation should be used in CVC catheterization, the elbow and other frequently moving positions should be avoided when catheterization, upper elbow with low activity, large diameter veins, fast blood flow rate and more blood flow should be selected. The catheter tip should be the lower third of the superior vena cava or the intersection of chambers<br>17. At present, CVC is not removed except for complications such as any longer indwelling CVC, blockage or dysfunction of CVC catheter, not within normal range, complicated with CRT-related blood flow infection, and so on.<br>18. It is recommended that CVC should be treated with anticoagulant therapy for 1 or 2 weeks before extubation, and then extubation should be anticoagulation at least 3 months after extubation. If CVC functions are normal and decide to continue to use it, even if the anticoagulant period has reached 3 months, it is necessary to continue anticoagulation until the CVC catheter is completely removed.

Knowledge training	medical staff	2、9	20. Learn CRT diagnosis, prevention related knowledge, master the correct treatment process, participate in bedside rounds and medical record discussions, participate in other healthcare organizations and expert academic exchanges
	Patient	2、3、9	21. Inform patients of disease-related knowledge, such as physical preventive measures, related symptoms and signs, side effects of drugs, emergency management, etc.
	Multi-disciplinary participation	2、3、5、7	22. Multi-disciplinary sharing, active organization of book exchange meetings, regular academic sharing.

Table S5. Summary of views on preventive measures included in the guidelines

Number	Advice and recommendation	Do not recommend comments
1	In order to reduce the injury caused by CVC, the catheter with small diameter and small number of lumens should be selected as much as possible; ultrasonic guidance should be used to reduce the number of catheterization in CVC catheterization; the elbow joint that often moves should be avoided in CVC catheterization and the upper elbow should be selected; The middle and lower 1/3 of superior vena cava and the intersection of chambers and chambers is the best intubation choice at the tip of CVC; early active after CVC catheterization. The indexes of blood routine and coagulation function and related symptoms and signs were monitored in time after the use of anticoagulant drugs.	Prophylactic use of anticoagulant or thrombolysis drugs is not recommended. Chest enhancement CT and three-dimensional lung imaging are not recommended to screen CRT patients for pulmonary embolism.
2	The prevention of CRT in cancer patients can be based on the combination of drug prevention and mechanical prevention; drug prevention can use heparin (low molecular weight heparin, unfractionated heparin) drugs; CRT high risk patients should be evaluated according to the actual situation after they can decide the time to start anticoagulation; CRT high risk	Mechanical prophylaxis is not recommended as a single measure of VTE prevention; anticoagulant is not

	patients (major surgery patients and cancer patients) should be given health education.	recommended to improve the survival rate of cancer patients without VTE symptoms.
3	CVC function is normal, the catheter can be retained; all patients should be treated with anticoagulant treatment after removing CVC for at least 3 months; if the anticoagulant time is more than 3 months after CVC catheterization, anticoagulation should continue until the catheter is removed.	For patients with deep venous thrombosis at risk of post-thrombotic syndrom , routine use of elastic socks is not recommended.
4	Risk factors for CRT formation include catheter factors (catheter material, placement techniques, location and type of catheter) and potential co-disease factors (antiangiogenic therapy, hormone therapy, parenteral nutrition and radiotherapy), as well as patient factors (age, sex and thrombosis-related diseases) The first choice of prevention after CVC intubation is to use low molecular heparin anticoagulant therapy for 3-6 months, if CVC has not been removed for more than 3 months, then low molecular heparin is used for anticoagulation indefinitely; direct oral	Except for CVC related infection, unsuitable anticoagulant, poor anticoagulant effect and decision to remove CVC, CVC transfer is not recommended..

	anticoagulant DOAC is also recommended; if anticoagulant therapy is ineffective or anticoagulant failure, CVC should be removed after 5 or 7 days of anticoagulation.	
5	If the CVC function is normal, you can continue to use it. When CVC is retained, it is recommended that anticoagulant therapy be carried out for CRT formation; if CVC is no longer needed, anticoagulant therapy should be performed 5 or 7 days before CVC removal; if CVC related infection, CRT or worsening symptoms, CVC should be removed when there is anticoagulant contraindication, and the risk of thrombosis should be taken into account, and the anticoagulant treatment should be decided according to the specific situation. In order to prevent CRT, cancer patients should be treated with anticoagulant therapy for at least 3 months, or anticoagulant therapy should be performed throughout the catheterization period; medical personnel should inform patients of the risks and benefits of anticoagulant, and determine the duration of treatment after being known to the patient.	Prophylactic anticoagulant therapy for patients with CVC intubation is not recommended.
6	The use of anticoagulant drugs alone in the treatment of symptomatic CRT in children can minimize complications and do not need to be pulled out of the catheter; for non-functional or no longer used CVC, it is recommended that anticoagulant	Multiple CVC intubation is not recommended; routine use of elastic

	therapy remove CVC a few days later.	socks is not recommended.
7	The right jugular vein is the first choice for CVC catheterization, in which the intersection of the right atrium and the superior vena cava is the best position for the distal tip of the catheter; for patients with PICC catheterization, the implantation port should be used; for symptomatic CRT, anticoagulant therapy should be performed for at least 3 months as long as CVC is in situ; there is no guideline to compare the anticoagulant effects of low molecular weight heparin, DOAC and vitamin K antagonists If the CVC catheter is unblocked the position of the catheter is normal, no infection occurs, and other symptoms disappear during anticoagulant, CVC can remain in place.	Undifferentiated anticoagulant therapy is not recommended for prevention of CRT formation.
8	When considering whether to use drugs to prevent CRT, it is necessary to analyze the risks and benefits of anticoagulant; LMWH or DOAC can be selected for CRT drug prevention; patients should be educated about VTE risk factors and related symptoms and signs.	Routine thrombosis prevention is not recommended in all patients; thrombosis prevention time is not recommended for less than 12 weeks.
9	The risk of CRT should be assessed before CVC intubation;	It is not

the appropriate intubation location and catheter type should be selected before catheterization; the selected veins should be observed and measured by ultrasound in advance before PICC intubation, and the catheter with the ratio of catheter diameter to vein diameter less than 45% should be preferred; the tip of CVC should be located at 1/3 of superior vena cava or the junction of vena cava and atrial vein; and the circumference of upper arm should be measured before insertion of PICC to assess the presence of edema and deep venous thrombosis. When CVC is located correctly, functionally normal and has no sign of infection, CVC does not need to be removed even if deep venous thrombosis exists.	recommended that CVC intubation be located in the anterior cubital fossa and prophylactic anticoagulant therapy is not recommended.

Zhang J, *et al. BMJ Open* 2024; 14:e074854. doi: 10.1136/bmjopen-2023-074854
