# Supplementary material for: Appraising the quality standard of clinical practice guidelines related to central venous catheter-related thrombosis prevention: a systematic review of clinical practice guidelines
Source: BMJ Open. 2024 Mar 11;14(3):e074854. doi: 10.1136/bmjopen-2023-074854 (PMC10936513; doi:10.1136/bmjopen-2023-074854)
Supplement: Supplementary data [file bmjopen-2023-074854supp001.pdf]

Pubmed Search Strategy

No	Search query
#1	Catheter-related thrombosis[Title/Abstract]
#2	Catheter-related thromboembolism[Title/Abstract]
#3	peripherally inserted central catheter-related thrombosis[Title/Abstract]
#4	venous thromboembolism[Title/Abstract]
#5	venous thrombosis[Title/Abstract]
#6	vein thrombosis[Title/Abstract]
#7	Catheter-related thrombosis[MeSH Terms]
#8	Catheter-related thromboembolism[MeSH Terms]
#9	peripherally inserted central catheter-related thrombosis[MeSH Terms]
#10	venous thromboembolism[MeSH Terms]
#11	venous thrombosis[MeSH Terms]
#12	vein thrombosis[MeSH Terms]
#13	#1 OR #2 OR #3 OR #4 OR #5 OR #6 OR #7 OR #8 OR #9 OR #10 OR #11 OR #12
#14	((clinical practice guideline[Title/Abstract]) OR (guideline[Title/Abstract])) OR (Evidence-Based Practice[Title/Abstract]) Filters: from 2017 - 2022
#15	#13 AND #14

Embase Search Strategy

No	Search query
1	Catheter-related thrombosis.m_titl.
2	Catheter-related thromboembolism.m_titl.
3	peripherally inserted central catheter-related thrombosis.m_titl.
4	venous thromboembolism.m_titl.
5	venous thrombosis.m_titl.
6	vein thrombosis.m_titl.
7	Catheter-related thrombosis.mp.
8	Catheter-related thromboembolism.mp.
9	peripherally inserted central catheter-related thrombosis.mp.
10	venous thromboembolism.mp.
11	venous thrombosis.mp.
12	vein thrombosis.mp.
13	1 or 2 or 3 or 4 or 5 or 6 or 7 or 8 or 9 or 10 or 11 or 12
14	clinical practice guideline.m_titl.
15	guideline.m_titl.
16	Evidence-Based Practice.m_titl.
17	14 or 15 or 16
18	13 and 17
19	limit 18 to yr="2017 - 2022"

Cochrane librarySearch Strategy

No	Search query
1	Catheter-related thrombosis OR Catheter-related thromboembolism OR peripherally inserted central catheter-related thrombosis in Title Abstract Keyword
2	venous thromboembolism OR venous thrombosis OR vein thrombosis in Title Abstract Keyword
3	Catheter-related thrombosis OR Catheter-related thromboembolism OR peripherally inserted central catheter-related thrombosis in Abstract
4	venous thromboembolism OR venous thrombosis OR vein thrombosis in Abstract
5	clinical practice guideline OR guideline OR Evidence-Based Practice in Title Abstract Keyword
6	1 OR 2 OR 3 OR 4 AND 5
7	Custom Range:01/01/2017 to 26/03/2022

Web of Science Search query:

((KP=(Catheter-related thrombosis OR Catheter-related thromboembolism OR peripherally inserted central catheter-related thrombosis OR venous thromboembolism OR venous thrombosis OR vein thrombosis)) OR KP=(Catheter-related thrombosis OR Catheter-related thromboembolism OR peripherally inserted central catheter-related thrombosis OR venous thromboembolism OR venous thrombosis OR vein thrombosis)) AND KP=(clinical practice guideline OR guideline OR Evidence-Based Practice)

publication date: 2017-01-01 to 2022-03-26

Up to date Search query:

(Catheter-related thrombosis OR Catheter-related thromboembolism OR peripherally inserted central catheter-related thrombosis OR venous thromboembolism OR venous thrombosis OR vein thrombosis)) OR (Catheter-related thrombosis OR Catheter-related thromboembolism OR peripherally inserted central catheter-related thrombosis OR venous thromboembolism OR venous thrombosis OR vein thrombosis)) AND (clinical practice guideline OR guideline OR Evidence-Based

Practice)

NICE Search query:
(Catheter-related thrombosis OR Catheter-related thromboembolism OR peripherally inserted central catheter-related thrombosis OR venous thromboembolism OR venous thrombosis OR vein thrombosis OR Catheter-related thrombosis OR Catheter-related thromboembolism OR peripherally inserted central catheter-related thrombosis OR venous thromboembolism OR venous thrombosis OR vein thrombosis) AND (clinical practice guideline OR guideline OR Evidence-Based Practice)
From date 2017/01/01 To date 2023/03/26

Wanfang search query:
((Title or keyword: (catheter-associated thrombosis) or Title or keyword: (catheter-related thromboembolism) or Title or keyword: (Thrombosis through peripheral venous placement of central venous catheter) or Title or keyword: (venous thromboembolism) or All: (venous thrombosis))) AND (Title or keyword: (clinical practice guidelines) or Title or keyword: (guideline) or Title or keyword: (evidence-based practice))). Search time: 2017.1.1~2022.3.26;

VIP: ((((Title or keyword=catheter-related thrombosis OR Title/keyword=catheter-related thromboembolism) OR Title or keyword=central venous catheter thrombosis via peripheral vein) OR (Title/keyword=venous thromboembolism OR title/keyword=venous thrombosis)) AND ((Title or keyword=clinical practice guideline=OR title/keyword=guideline) OR title/keyword=evidence-based practice)) AND (years:[2017 TO 2022])

CNKI:
(Subject: Catheter-associated thrombosis)OR (Subject: Catheter-associated thromboembolism)OR (Subject: Thrombosis of a central venous catheter inserted

through a peripheral vein)OR (Subject: Venous Thromboembolism)OR (Subject: Venous Thrombosis)AND (Subject: Clinical Practice Guidelines)OR (Subject: Guidelines)OR (Subject: Evidence-Based Practice). Publication time: 2017-01-01 to 2022-03-26

Medical Pulse Communication:

(((((Catheter-associated thrombosis) or (catheter-associated thromboembolism) or (peripherally inserted central venous catheter thrombosis) or (venous thrombosis) or (venous thrombosis)AND ((((Clinical Practice Guidelines) or (Guidelines) or (Evidence-Based Practice)

Chinese Biomedical Literature Database:

"Catheter-associated thrombosis" [Title: Smart] OR "Catheter-associated thromboembolism" [Title: Smart] OR "Thrombosis of a central venous catheter through a peripheral vein" [Title: Smart] OR "Venous Thromboembolism" [Title: Smart] OR "Venous Thrombosis" [Title: Smart] AND "Clinical Practice Guidelines" [Title: Smart] AND "Guideline" [Title: Smart] AND "Evidence-Based Practice" [Title: Smart].2017-2022
