# Supplementary material for: Appraising the quality standard of clinical practice guidelines related to central venous catheter-related thrombosis prevention: a systematic review of clinical practice guidelines
Source: BMJ Open. 2024 Mar 11;14(3):e074854. doi: 10.1136/bmjopen-2023-074854 (PMC10936513; doi:10.1136/bmjopen-2023-074854)
Supplement: Author's manuscript [file bmjopen-2023-074854.draft_revisions.pdf]

BMJ Open

BMJ Open is committed to open peer review. As part of this commitment we make the peer review history of every article we publish publicly available.

When an article is published we post the peer reviewers' comments and the authors' responses online. We also post the versions of the paper that were used during peer review. These are the versions that the peer review comments apply to.

The versions of the paper that follow are the versions that were submitted during the peer review process. They are not the versions of record or the final published versions. They should not be cited or distributed as the published version of this manuscript.

BMJ Open is an open access journal and the full, final, typeset and author-corrected version of record of the manuscript is available on our site with no access controls, subscription charges or pay-per-view fees (http://bmjopen.bmj.com).

If you have any questions on BMJ Open's open peer review process please email info.bmjopen@bmj.com

BMJ Open

Quality evaluation and content analysis of clinical practice guidelines related to central venous catheter-related thrombosis prevention

Journal:	*BMJ Open*
Manuscript ID	bmjopen-2023-074854
Article Type:	Original research
Date Submitted by the Author:	19-Apr-2023
Complete List of Authors:	Zhang, Jing; Sichuan University<br>Wu, Yongya; Sichuan University<br>Zhang, Shuai; Sichuan University<br>Yao, Wenmo; Sichuan University<br>Bu, Faqian; Sichuan University<br>Wang, Aoxue; Sichuan University<br>Hu, Xiuying; Sichuan University<br>Wang, Guan; Sichuan University
Keywords:	Thromboembolism < CARDIOLOGY, Systematic Review, VASCULAR MEDICINE

SCHOLARONE™
Manuscripts

I, the Submitting Author has the right to grant and does grant on behalf of all authors of the Work (as defined in the below author licence), an exclusive licence and/or a non-exclusive licence for contributions from authors who are: i) UK Crown employees; ii) where BMJ has agreed a CC-BY licence shall apply, and/or iii) in accordance with the terms applicable for US Federal Government officers or employees acting as part of their official duties; on a worldwide, perpetual, irrevocable, royalty-free basis to BMJ Publishing Group Ltd ("BMJ") its licensees and where the relevant Journal is co-owned by BMJ to the co-owners of the Journal, to publish the Work in this journal and any other BMJ products and to exploit all rights, as set out in our licence.

The Submitting Author accepts and understands that any supply made under these terms is made by BMJ to the Submitting Author unless you are acting as an employee on behalf of your employer or a postgraduate student of an affiliated institution which is paying any applicable article publishing charge ("APC") for Open Access articles. Where the Submitting Author wishes to make the Work available on an Open Access basis (and intends to pay the relevant APC), the terms of reuse of such Open Access shall be governed by a Creative Commons licence – details of these licences and which Creative Commons licence will apply to this Work are set out in our licence referred to above.

Other than as permitted in any relevant BMJ Author's Self Archiving Policies, I confirm this Work has not been accepted for publication elsewhere, is not being considered for publication elsewhere and does not duplicate material already published. I confirm all authors consent to publication of this Work and authorise the granting of this licence.

For peer review only

Quality evaluation and content analysis of clinical practice guidelines related to central venous catheter-related thrombosis prevention

Jing Zhang[1], Yongya Wu[1], Shuai Zhang[1], Wenmo Yao[1], Faqian Bu[1], Aoxue Wang[1], Xiuying Hu[1], Guan Wang[1]

[1] Innovation Center of Nursing Research, Nursing Key Laboratory of Sichuan Province, State Key Laboratory of Biotherapy and Cancer Center, West China Hospital, Sichuan University /West China School of Nursing, Sichuan University, Chengdu 610041, China.

Correspondence to

Dr Guan Wang; guan8079@163.com

Dr Xiuying Hu; huxiuying@scu.edu.cn

For peer review only

Abstract:

Background: Central venous catheter (CVC)operation is convenient and relatively safe, and it is widely used in clinical practice. Catheter-associated venous thrombosis is one of the common complications after CVC catheterization. Several clinical practice guidelines(CPGs) for Catheter-related thrombosis (CRT) prevention have been published, but their quality is unclear.

Objective: To evaluate the quality and analyze the content of clinical practice guidelines regarding central venous catheter-related thrombosis to provide evidence for formulating an evidence-based practice protocol and a risk assessment scale to prevent it.

Methods: Clinical practice guidelines regarding central venous catheter-related thrombosis prevention were searched in guidelines websites and databases such as National Institute for Health and Care Excellence, Up to date, Pubmed, Web of Science, CNKI, and others. The included guidelines were evaluated using AGREE II.

Results: A total of nine guidelines were included, and the quality grade results showed that three were at A-level and six were at B-level. The included guidelines are mainly recommended the prevention measure of central venous catheter-related thrombosis from three aspects: risk screening, prevention strategies, and knowledge training, with a total of 22 suggestions were recommended.

Conclusion: The overall quality of the guidelines is high, but there are few preventive measures for central venous catheter-related thrombosis involved in the guidelines. All preventive measures have not been systematically integrated and evaluated, and no risk assessment scale dedicated to this field has been recommended. Therefore it is urgent to

For peer review only

develop an evidence-based practice protocol and a risk assessment scale to prevent it.

Keywords: Central venous catheter; Catheter-related thrombosis; Venous thromboembolism; Clinical practice guidelines; Prevention

Introduction

The central venous catheter is catheter inserted through the proximal central vein or peripheral vein, with the catheter tip located at one third of the junction between the superior vena cava and the right atrium(1). The CVC includes subclavian vein/ jugular vein/femoral vein puncture catheters, peripherally inserted central catheter(PICC), and implantable venous access port(2). It has many advantages, such as convenient and safe operation, rapid establishment of intravenous access, reduction of puncture times, and infusion of irritating liquid; therefore it has been widely used in chemotherapy, hemodialysis, nutrition support, and surgery support(3–6).

Catheter-related thrombosis refers to the formation of blood clots on the outer wall or inner wall of catheter. CRT is one of the common complications after catheterization of CVC, which can lead to unplanned extubation and pulmonary embolism etc, affecting the progress of treatment and increasing the cost of treatment(7–10). The risk factors of CRT could be categorized into patient-related, diseases/treatment-related, and catheter-related factors. Patient-related factors include sex, advanced age, long-term bedridden, venous thromboembolism history, acquired or hereditary blood hypercoagulability state, etc(11–13). Diseases/treatment-related factors include the grade of cancer, chemotherapy, radiotherapy, parenteral nutrition support, surgical

For peer review only - http://bmjopen.bmj.com/site/about/guidelines.xhtml

trauma or excessive bleeding, etc(14). Catheter-related factors include catheter material, catheter-to vein ratio, puncture times, length of catheter indwelling, the location of catheter tip, etc(15).

CRT has the characteristics of high incidence, early onset and clinically silent symptoms(16). The clinical manifestations of CRT are as follows: deep venous thrombosis (DVT), superficial thrombophlebitis, asymptomatic thrombosis and thrombotic catheter failure. The study reported CRT incidence ranged markedly from 1.9 to 73.0%(5,8,15–17). It could happen as early as the first day after catheterization(16,18), the average time of occurrence was within 2 weeks after catheterization（5, 10, 19, 20）. However, the early symptoms and signs were silent, only a few patients showed clinical symptoms. Due to the limited medical resources, vascular surgery in most medical institutions in China can not monitor the occurrence of CRT in time, and can not correctly prevent and deal with CRT, resulting in the increase of hospitalization time, resulting in irreversible effects, and affecting the quality of follow-up treatment of patients.

Therefore, assessment and taking measurements early to prevent patients at high risk of CRT risk are essential to ensure the safety of patients. Clinical Practice guideline is formulated by clinical workers after systematically integrating and evaluating relevant research and clinical experience according to evidence-based concept to a certain common symptom, disease or problem, to provide guiding suggestions for clinical workers and patients, and scientifically promote the application of evidence in clinical practice(21). At present, several clinical practice guidelines

For peer review only - http://bmjopen.bmj.com/site/about/guidelines.xhtml

about CRT prevention have been published, but their quality is unclear. Therefore, through the systematic retrieval, screening and reading of clinical practice guidelines related to CRT prevention, and the quality evaluation and content analysis of the guidelines, this review was expected to provide scientific basis for the development of evidence-based practice protocols and risk assessment scales for CRT.

Information and methods

Search strategy

Search databases

We searched in both English and Chinese databases, including Pubmed, Embase, Cochrane library, Web of Science, CNKI, Wanfang, VIP, and Chinese Biomedical Literature Database from January 1st, 2017 to March 26th, 2022. In addition, the relevant websites of the guideline, including up to date, National Institute for Health and Care Excellence (NICE), Medline, were searched. The manual search of relevant studies, and bibliography was also adopted.

Search strategies

The literature retrieval adopted strategy of the combination of MeSH terms and free words. The MeSH terms and synonyms of catheter-related thrombosis and clinical practice guideline were searched in English and Chinese databases. Take Pubmed as the retrieval representative of English database. The search strategy is shown in table S1.

Inclusion and exclusion criteria

For peer review only - http://bmjopen.bmj.com/site/about/guidelines.xhtml

The inclusion criteria were (1) guidelines including the contents of treatment, prevention or management of CRT; (2) guidelines published or updated after Jaunary 1st,2017; and (3) guidelines published in English or Chinese. The exclusion criteria were (1) guidelines developed by expert consensus exclusively; (2) incomplete guidelines that are in the process of development; (3) the translation or reinterpreting of the guidelines.

Guideline screening and data extraction

The literature was searched by two researchers who had received systematic literature retrieval and training. The searched literature was imported into Endnote X9 software and removed the duplicates. Then, two researchers independently read the title and abstracts for preliminary screening to exclude obviously unrelated articles. Subsequently, two reviewers read the full text carefully and screen it again according to the established criteria for inclusion and exclusion. In case of disagreement, the third party will be consulted before determining. Finally, a predefined data extraction table was used to extract the data from the included studies.

Quality appraisal

The AGREE II scale, a widely used tool for evaluating the quality of the guideline, was used to evaluate the included guidelines(22). It includes 6 domains, i.e. scope and purpose, rigorism, independence, clarity, application, and independence of writing, with 23 items. Each item has a maximum score of seven and a minimum of one. Seven indicates that the evaluator is fully satisfied with the area covered by the entry, and one

For peer review only - http://bmjopen.bmj.com/site/about/guidelines.xhtml

indicates that the evaluator is completely dissatisfied with the field. The higher the evaluator is satisfied with the area covered by the guideline, the more scores. The evaluator should score the satisfaction degree of the guideline according to the actual situation to ensure the fairness of the guideline. Two researchers who systematically trained the use methods of AGREE II scale were selected to evaluate the guideline to ensure that the researchers had a full understanding and consistent understanding of the items and to reduce the biases of evaluation. If there is a dispute over an item, experts in the relevant field would be consulted to resolve it. The standard rate of the two researchers in each field of the guideline was calculated, and the systematic evaluation and content analysis and summary of the guideline were carried out based on the evidence-based concept. Level A recommended the guideline, indicating that most entries in the guideline scored ≥ 5, and its standardized percentage ≥ 60%. Level B was recommended after modification, and indicated that the standard rate in more than three areas was 30-60%. Level C, indicated that the guideline was not recommended, and indicated that the standardized rate in three or more areas was less than 30%.

Statistical methods

SPSS22.0 was used to count the scores of the two researchers in each field of the guideline, and the Intraclass Correlation Coefficient (ICC) of the data was calculated. According to the results, the consistency of the two researchers' evaluation of the guideline was tested. ICC < 0.4 indicated poor consistency, 0.40 ≤ 0.59 indicated that the consistency is general, 0.60 ≤ 0.74 indicated good consistency, and > 0.74 indicated that the consistency is very good.

For peer review only - http://bmjopen.bmj.com/site/about/guidelines.xhtml

Result

Guideline retrieval results

A total of 2092 articles were retrieved, and 571 articles were removed by software and manual deletion. After read the title and abstract, 1142 articles were excluded. 379 articles were screened according to inclusion and exclusion criteria. 267 articles that did not meet the theme of the article were excluded, and 5 related guidelines were traced through the snowball strategy, and 117 articles were finally selected. Among them, 11 articles were not obtained, and a total of 106 complete articles were obtained. Full-text screened excluded 18 non-evidence-based guidelines issued in individual form, 6 guidelines that had been updated, and 73 guidelines that did not include CRT treatment, prevention, and management. Finally, 9 literatures were selected, including 2 Chinese literatures and 7 English literatures. The specific screened process is shown in Figure S1.

Basic characteristics of inclusion in the guideline

The nine guidelines were from the American Society of Clinical Oncology, the International Initiative on Thrombosis and Cancer, the Chinese Medical Association, the Spanish Medical Oncology Society, the Infusion Nursing Association, and other international authoritative medical associations. Only one of them is a guideline targeted for CRT risk assessment, prevention, and intervention[18]. Five guidelines were specifically for venous thrombosis prevention in cancer patients(14,22–25), and the

For peer review only - http://bmjopen.bmj.com/site/about/guidelines.xhtml

content related to CRT was only part of it. Two of which are guidelines for venous thrombosis of ordinary patients(26,31). CRT patients are part of the content, and only one guideline is targeted for clinical infusion therapy(27). CRT is part of the treatment (see Table S2).

Consistency between AGREEII evaluation results

After scoring the nine guidelines with the AGREEII tool, it was found that the recommended A-level guidelines were three(22,23,28). The recommended B-level guidelines are six(14,25–27). An analysis of the data showed that the highest score of clarity in the guideline was 80.87%, followed by scope and purpose (79.01%), independence of the writing (70.74%), rigorism and independence of the guideline (69.91%), participants (67.90%), and application (64.08%). The details are shown in Table S3. After the ICC calculation of the scores of each guideline by two evaluators, it was concluded that the ICC of the two evaluators for the nine guidelines was more than 0.8, and indicated that the consistency among the evaluators is good.

Recommendations for the guideline

Read all the recommendations and precautions in the included guidelines, it was found that the content of CRT prevention can be divided into three aspects: risk assessment, preventive measures, and health education. The specific contents are shown in Table S4.

Comparison of CRT prevention measures in the guidelines

Due to the differences in the topics and subjects of the guideline, the emphasis and

For peer review only - http://bmjopen.bmj.com/site/about/guidelines.xhtml

level of detail of the CRT related prevention strategies recommended by each guideline vary. Therefore, in order to better analyze and compare preventive measures, the relevant aspects of CRT prevention in the nine guidelines to be included are summarized in Table S5

Discussion

Basic characteristics of CRT Prevention related guidelines

The guidelines included in the study are widely used and have high impact factors. Guidelines issued by the China Branch of International Union of Angiology, for the first time, the preventive measures of CRT were systematically summarized, which filled the gap in this field, but the methodological quality was poor. Guidelines issued by ASCO, SEOM, ASH, NCCN, ITAC-CME(14,22,23,28). This paper mainly expounds the preventive measures of CRT from two aspects of drug prevention and CVC-related prevention, including the choice of anticoagulant drugs, the time of continuous anticoagulant treatment, and the choice of catheter location; however, these were only a small part of the guideline and the content were scattered in the included guidelines. Five included guidelines were targeted for cancer patients, and had certain limitations. Guidelines issued by the Chinese Medical Association and NICE(26,31), mainly put forward some suggestions on how to prevent various types of thrombosis, and the prevention measure for CRT was one of them, including drug prevention of CRT, CVC catheter related prevention and physical prevention. The guideline issued by INS described the latest clinical infusion treatment practice standards and specific

For peer review only - http://bmjopen.bmj.com/site/about/guidelines.xhtml

measures(27). The prevention of CVC related complications was part of them, which mainly describes how to deal with CRT.

Quality evaluation of the guidelines

In order to ensure the quality of the guidelines, researchers should follow the principles of professionalism, fairness, evidence-based and transparency when developing the guideline. As the guidelines included in this study are of good quality and high impact factors, the AGREEII scale is used to evaluate the six areas of the guideline, and it is found that the clarity, scope and purpose of the guidelines have reached the standard level, while the rigorism and independence, participants and application of the guidelines need to be further improved, the specific analysis was as follows:

rigorism and independence

In the aspect of rigorism, the expert consensus was released by the China Branch of International Union of Angiology. The guideline do not systematically retrieve CRT prevention related research, but only summarize the "experience" of clinical experts, lack of corresponding evidence support, and lack of scientific and rigorous. The guidelines issued by NCCN and NICE(22,25),1/1/0001 12:00:00 AM systematically evaluated the relevant research and conclusions, and recommended opinions and suggestions. But these two guidelines did not point out the steps and methods of systematic retrieval of evidence, as well as the criteria for accepting and excluding the research. In addition to the guidelines issued by INS(27), the rest of the guidelines do not specify the steps and methods for updating the guidelines. But the guidelines

For peer review only - http://bmjopen.bmj.com/site/about/guidelines.xhtml

indicated the timing of the update(27), and the extent to which the evidence level of the new guideline should be higher than that of the previous edition of the guideline, and indicated that the quality of the evidence in the guideline is constantly improving. Due to the differences in medical and health systems at home and abroad and the non-standard evaluation of guidelines, the study found that most of the guidelines published abroad put forward statements of interest in the study(14,22,23,25,27,28). However, the guideline issued by the China Branch of International Union of Angiology failed to state the statement of interests of the study. The interests of the panel members with other bodies are also not recorded and the fairness and independence of the study are not guaranteed. Therefore, we should pay full attention to the rigor and independence of the guideline, pursue evidence according to the concept of evidence-based medicine, avoid the influence of external factors and the biases of research caused by personal preferences, to ensure the rigour and independence of research.

participants

In the participant section, only two guidelines issued by the Chinese Medical Association and INS(27,31), the names and work units of all the experts involved in the formulation of the guidelines are listed, while the rest of the guidelines refer only to interdisciplinary teams or association support and without specify the relevant information of the researchers. In addition to the release guidelines of the Chinese Medical Association, NCCN, and ITAC-CME(23,25,31), the rest of the team did not explain how to collect comments and suggestions from users considering the relevant information of researchers, such as work experience, units and professional titles, can

For peer review only - http://bmjopen.bmj.com/site/about/guidelines.xhtml

reduce the bias caused by personal factors of researchers, which is conducive to the formulation of guidelines and reflects the universality and authority of the formulation of guidelines.

application

In the field of application, relevant studies have shown that the use of clinical guidelines is generally low. Most of the guidelines included in this study do not fully take into account the difficulties and obstacles that may be encountered in the follow-up application, nor are they accompanied by relevant systems and documents, and there is no training for clinical staff in the use of the guidelines, which is not conducive to the learning and use of the guidelines. Of the included guidelines, only this issued by NICE is presented(26). The guidelines are applied to specific clinical methods and measures, but the rest of the guidelines do not address the forward and reverse factors in the application of the guidelines and how to make use of potential resources, and fail to transform the theory into practice well. Therefore, it is necessary to carry out pre-experiments in the process of guideline formulation in the future, check the feasibility of guidelines recommendations in advance, collected the problems in the application of guidelines in advance, make corresponding preparations, and promote the transformation of guidelines to clinical practice.

analysis of the recommendations in guidelines

risk screening

Timely assessment of risk factors for CRT formation in patients is beneficial to clinical prevention of CRT formation, in addition to the guidelines issued by SEOM

For peer review only - http://bmjopen.bmj.com/site/about/guidelines.xhtml

and ITAC-AEM(14,23), the rest of the guidelines indicate that CRT risk factors need to be assessed before CVC intubation. The guidelines also clearly point out the high risk factors of CRT, mainly divided into patient-, catheter-, and disease/treatment-related factors. The patient-related factors were age, sex, family history or personal history of venous thrombosis, hypercoagulable state of blood, and abnormal coagulation gene. Catheter-related factors were catheter material, placement technique, location and type of catheter, and puncture times. And disease/treatment-related factors were antiangiogenic therapy, hormone therapy, parenteral nutrition and radiotherapy, tumor and trauma and complicated surgery. However, at present, there is no recognized scale dedicated to CRT risk assessment. Most of the guidelines recommend the use of VTE risk factor assessment scale, and the two guidelines recommend the use of Khorana scale and wells scale(22,23,31). The guideline issued by NCCN recommends that patients with medical diseases can use the Padua scale(25). At the same time, it is suggested that myeloma patients should be treated with targeted anticoagulant therapy after scoring with IMPED and SAVED scales. CRT risk assessment should be carried out at admission, before and after catheterization and replacement, and when symptoms and signs occurred. Timely evaluation of CRT related symptoms of patients, regular examination of related physiological and biochemical indicators, according to the actual situation flexible adjustment of anticoagulant program, the occurrence of special circumstances timely report.

Prevention strategies

Through the summary and analysis of the nine guidelines, it is found that the

For peer review only - http://bmjopen.bmj.com/site/about/guidelines.xhtml

preventive measures are mainly divided into the following five parts: classified prevention, physical prevention, drug prevention, common CRT prevention measures, and multi-disciplinary prevention. Classified prevention emphasizes individualized prevention according to the patients' own factors and the clinical manifestations of CRT. Physical prevention is mainly to help patients exercise actively or passively, promote their blood circulation, to reduce the formation of thrombosis. In order to urge patients to get out of bed as soon as possible, put tube side limbs to carry out functional exercises such as repeated clenching or loosening, promote blood circulation, timely replenish water, and prevent dehydration and blood coagulation. However, most guidelines indicate that patients at risk of post-thrombosis syndrome do not recommend routine use of elastic socks for prevention, as they may aggravate skin breakage and injury. Drug prevention mainly uses low-dose unfractionated heparin, low molecular weight heparin, and vitamin K antagonists, while some guidelines recommend the use of a new direct oral anticoagulant DOAC. The risk of bleeding should be taken into account when anticoagulants are used, and the risk and benefits of drug should be assessed and used with the informed consent of the patient. The common prevention of CRT is mainly the treatment of catheter, including the choice of catheter diameter and lumen, the way of catheterization, the location of catheterization, the time of catheterization and subsequent anticoagulant therapy, as shown in tables S4 and S5. Multi-disciplinary prevention refers to the construction of a multi-disciplinary team composed of vascular surgery, oncology, evidence-based medicine center, medical laboratory technicians, and nursing to carry out CRT prevention, so as to avoid the

For peer review only

limitations of clinical practice.

knowledge training

The guideline issued by INS suggests that nurses should constantly update their clinical knowledge and skills[39], and based on the concept of evidence-based transformation of the latest research into high-quality evidence, to promote its transformation to clinical practice. At present, the update of clinical nursing is slow, and most of the medical staff are still based on the traditional nursing methods, so it is difficult to introduce the new research results into clinical practice. Therefore, it is very necessary to carry out systematic scientific research education, organize reading exchange meetings and knowledge training for nurses. The guidelines issued by ASCO(22) 1/1/0001 12:00:00 AMindicate that, although CRT is closely related to cancer, patients' awareness of the risks and warning signals of CRT is still very low, emphasizing the need to increase patient education and awareness(24,29). At the same time, a study suggests that patients may not report new symptoms unless asked directly, as they usually consider symptoms to be a manifestation of cancer or a side effect of treatment. Therefore, health education for patients is helpful to ensure effective communication between medical staff and patients, and is conducive to patients' understanding of the disease(30).

limitations of the guidelines.

Although one of the nine guidelines included in this study is a specific guideline on CRT prevention, it does not systematically retrieve relevant research and evidence, and only recommends and compiles guidelines based on the experience of clinical

For peer review only - http://bmjopen.bmj.com/site/about/guidelines.xhtml

experts, which is lack of scientific. Most of the other guidelines are suitable for cancer patients, and the main prevention strategies related to CRT are drug prevention and CVC catheter prevention, but their contents are less and their views are scattered, the subjects of the guidelines are limited and lack of systematic integrated evaluation. Therefore, the formulation of evidence-based CRT prevention related clinical practice guidelines is helpful to guideline clinical staff to standardize the operation and ensure the treatment quality of patients. At the same time, after analyzing the content of the guideline, it is found that there is no tool dedicated to CRT risk assessment. At present, the guidelines recommend the use of mature VTE risk assessment scale, but this kind of scale does not involve the evaluation of CVC catheter, which is not targeted, which may lead to the bias of measurement. Therefore, the development of a risk assessment scale for CRT is conducive to the accurate selection of appropriate preventive measures and anticoagulant treatment methods, and reduces the injury caused to patients after the occurrence of CRT.

Conclusion

The overall quality of the nine guidelines included is good, and the corresponding advice and suggestions about CRT prevention are provided, but the guidelines still need to be improved in terms of rigorism and independence of writing, participants and applications. At present, there is still a lack of high-quality clinical practice guidelines for CRT prevention, and there is also a lack of dedicated CRT risk assessment tool.

For peer review only - http://bmjopen.bmj.com/site/about/guidelines.xhtml

Therefore, there is an urgent need to develop evidence-based CRT prevention related clinical practice guidelines and risk assessment tools based on the clinical situation to enable patients to participate in the management of CRT, thereafter to to better manage the CRT.

Contributors

GW and XH conceived and supervised the project. JZ,YW and SZ summed up the literature and drafted the manuscript. WY,FB and AW drew the figure and tables. GW and JZ revised the manuscript. All authors contributed, edited and approved the final manuscript.

Funding

This work was supported by the Sichuan Science and Technology Program (Grant 2022NSFSC1290), and West China Nursing Discipline Development Special Fund Project, Sichuan University (Grant HXHL21011).

Competing interests

The authors declare that they have no conflicts of interest.

Patient and public involvement

Patients and/or the public were not involved in the design, or conduct, or reporting, or dissemination plans of this research.

For peer review only

For peer review only - http://bmjopen.bmj.com/site/about/guidelines.xhtml

Patient consent for publication

Not required

Provenance and peer review

Not commissioned; externally peer reviewed

Supplemental material

This content has been supplied by the author(s). It has not been vetted by BMJ Publishing Group Limited (BMJ) and may not have been peer-reviewed. Any opinions or recommendations discussed are solely those of the author(s) and are not endorsed by BMJ. BMJ disclaims all liability and responsibility arising from any reliance placed on the content. Where the content includes any translated material, BMJ does not warrant the accuracy and reliability of the translations (including but not limited to local regulations, clinical guidelines, terminology, drug names and drug dosages), and is not responsible for any error and/or omissions arising from translation and adaptation or otherwise.

Open access

This is an open access article distributed in accordance with the Creative Commons Attribution Non Commercial (CC BY-NC 4.0) license, which permits others to distribute, remix, adapt, build upon this work non-commercially, and license their

For peer review only - http://bmjopen.bmj.com/site/about/guidelines.xhtml

derivative works on different terms, provided the original work is properly cited, appropriate credit is given, any changes made indicated, and the use is non-commercial. See: http://creativecommons.org/licenses/by-nc/4.

For peer review only

References

1. Smith RN, Nolan JP. Central venous catheters. *BMJ.* 2013 Nov 11;347:f6570.

2. Expert Group on Safety Management of Central Venous Access Device. [The expert consensus on safety management of central venous access device]. *Chinese Journal of Surgery.* 2020 Apr 1;58(4):261–72.

3. Matar CF, Kahale LA, Hakoum MB, Tsolakian IG, Etxeandia-Ikobaltzeta I, Yosuico VE, et al. Anticoagulation for perioperative thromboprophylaxis in people with cancer. *Cochrane Database Syst Rev.* 2018 Jul 11;7(7):CD009447.

4. Hao N, Xie X, Zhou Z, Li J, Kang L, Wu H, et al. Nomogram predicted risk of peripherally inserted central catheter related thrombosis. *Sci Rep.* 2017 Jul 24;7(1):6344.

5. Liu Y, Gao Y, Wei L, Chen W, Ma X, Song L. Peripherally inserted central catheter thrombosis incidence and risk factors in cancer patients: a double-center prospective investigation. *Ther Clin Risk Manag.* 2015;11:153–60.

6. Meyer BM. Developing an alternative workflow model for peripherally inserted central catheter placement. *J Infus Nurs.* 2012;35(1):34–42.

7. Takemoto CM, Sohi S, Desai K, Bharaj R, Khanna A, McFarland S, et al. Hospital-associated venous thromboembolism in children: incidence and clinical characteristics. *J Pediatr.* 2014 Feb;164(2):332–8.

8. Chen Y, Chen H, Yang J, Jin W, Fu D, Liu M, et al. Patterns and risk factors of peripherally inserted central venous catheter-related symptomatic thrombosis events in patients with malignant tumors receiving chemotherapy. *J Vasc Surg Venous Lymphat Disord.* 2020 Nov;8(6):919–29.

9. Chopra V, Anand S, Krein SL, Chenoweth C, Saint S. Bloodstream infection, venous thrombosis, and peripherally inserted central catheters: reappraising the evidence. *Am J Med.* 2012 Aug;125(8):733–41.

10. Marnejon T, Angelo D, Abu Abdou A, Gemmel D. Risk factors for upper extremity venous thrombosis associated with peripherally inserted central venous catheters. *J Vasc Access.* 2012;13(2):231–8.

11. Chopra V, Anand S, Hickner A, Buist M, Rogers MA, Saint S, et al. Risk of venous thromboembolism associated with peripherally inserted central catheters: a systematic review and meta-analysis. *Lancet.* 2013 Jul 27;382(9889):311–25.

For peer review only - http://bmjopen.bmj.com/site/about/guidelines.xhtml

12. Lowell JA, Bothe A. Central venous catheter related thrombosis. *Surg Oncol Clin N Am.* 1995 Jul;4(3):479–92.

13. Tran BH, Nguyen TJ, Hwang BH, Vidar EN, Davis GB, Chan LS, et al. Risk factors associated with venous thromboembolism in 49,028 mastectomy patients. *Breast.* 2013 Aug;22(4):444–8.

14. Ferro JM, Bousser MG, Canhão P, Coutinho JM, Crassard I, Dentali F, et al. European Stroke Organization guideline for the diagnosis and treatment of cerebral venous thrombosis - endorsed by the European Academy of Neurology. *Eur J Neurol.* 2017 Oct;24(10):1203–13.

15. Jones D, Wismayer K, Bozas G, Palmer J, Elliott M, Maraveyas A. The risk of venous thromboembolism associated with peripherally inserted central catheters in ambulant cancer patients. *Thromb J.* 2017;15:25.

16. Chen H, Tao L, Zhang X, Jing W, Su X, Chen H, et al. The effect of systemic and local risk factors on triggering peripherally inserted central catheter-related thrombosis in cancer patients: A prospective cohort study based on ultrasound examination and structural equation modeling. *Int J Nurs Stud.* 2021 Sep;121:104003.

17. Ahn DH, Illum HB, Wang DH, Sharma A, Dowell JE. Upper extremity venous thrombosis in patients with cancer with peripherally inserted central venous catheters: a retrospective analysis of risk factors. *J Oncol Pract.* 2013 Jan;9(1):e8-12.

18. Wang G, Li Y, Wu C, Guo L, Hao L, Liao H, et al. The clinical features and related factors of PICC-related upper extremity asymptomatic venous thrombosis in cancer patients: A prospective study. *Medicine (Baltimore).* 2020 Mar;99(12):e19409.

19. Bonizzoli M, Batacchi S, Cianchi G, Zagli G, Lapi F, Tucci V, et al. Peripherally inserted central venous catheters and central venous catheters related thrombosis in post-critical patients. *Intensive Care Med.* 2011 Feb;37(2):284–9.

20. Chopra V, Kaatz S, Grant P, Swaminathan L, Boldenow T, Conlon A, et al. Risk of Venous Thromboembolism Following Peripherally Inserted Central Catheter Exchange: An Analysis of 23,000 Hospitalized Patients. *Am J Med.* 2018 Jun;131(6):651–60.

21. Institute of Medicine (US) Committee to Advise the Public Health Service on Clinical Practice Guidelines. Clinical Practice Guidelines: Directions for a New Program. *National Academies Press (US).* 1990. doi:10.17226/1626

22. Key NS, Khorana AA, Kuderer NM, Bohlke K, Lee AYY, Arcelus JI, et al. Venous Thromboembolism Prophylaxis and Treatment in Patients With Cancer:

For peer review only - http://bmjopen.bmj.com/site/about/guidelines.xhtml

ASCO Clinical Practice Guideline Update. *J Clin Oncol.* 2020 Feb 10;38(5):496–520.

23. Farge D, Frere C, Connors JM, Ay C, Khorana AA, Munoz A, et al. 2019 international clinical practice guidelines for the treatment and prophylaxis of venous thromboembolism in patients with cancer. *Lancet Oncol.* 2019 Oct;20(10):e566–81.

24. Sousou T, Khorana AA. Cancer patients and awareness of venous thromboembolism. *Cancer Invest.* 2010 Jan;28(1):44–5.

25. Streiff MB, Holmstrom B, Angelini D, Ashrani A, Elshoury A, Fanikos J, et al. Cancer-Associated Venous Thromboembolic Disease, Version 2.2021, NCCN Clinical Practice Guidelines in Oncology. *J Natl Compr Canc Netw.* 2021 Oct 15;19(10):1181–201.

26. Venous thromboembolism in over 16s: reducing the risk of hospital-acquired deep vein thrombosis or pulmonary embolism. *London: National Institute for Health and Care Excellence (NICE).* 2019 Aug 13.

27. Hinck SM. Implementing the Infusion Therapy Standards of Practice. *Home Healthc Now.* 2021 Oct 1;39(5):295.

28. Witt DM, Nieuwlaat R, Clark NP, Ansell J, Holbrook A, Skov J, et al. American Society of Hematology 2018 guidelines for management of venous thromboembolism: optimal management of anticoagulation therapy. *Blood Adv.* 2018 Nov 27;2(22):3257–91.

29. Aggarwal A, Fullam L, Brownstein AP, Maynard GA, Ansell J, Varga EA, et al. Deep vein thrombosis (DVT) and pulmonary embolism (PE): awareness and prophylaxis practices reported by patients with cancer. *Cancer Invest.* 2015;33(9):405–10.

30. Gilligan T, Coyle N, Frankel RM, Berry DL, Bohlke K, Epstein RM, et al. Patient-Clinician Communication: American Society of Clinical Oncology Consensus Guideline. *J Clin Oncol*. 2017 Nov 1;35(31):3618–32.

31.Chinese Guidelines for the prevention and treatment of thrombotic Diseases. *Chin J Med*. 2018; 98 (36) : 2861-2888.

For peer review only - http://bmjopen.bmj.com/site/about/guidelines.xhtml

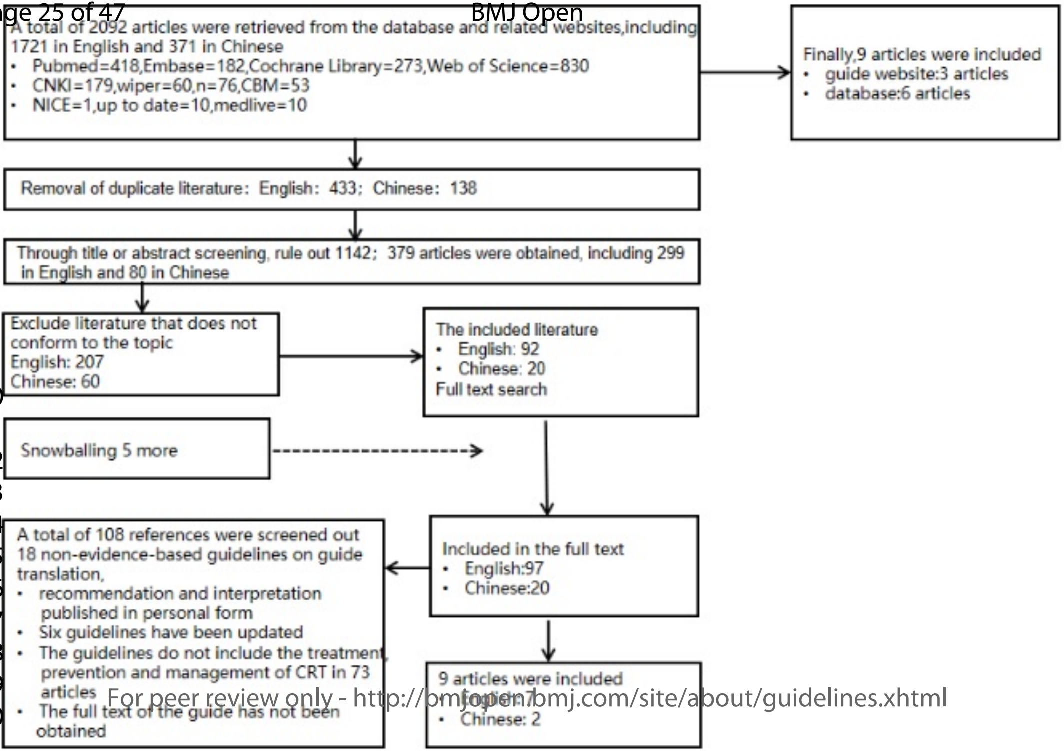

For peer review only - http://bmjopen.bmj.com/site/about/guidelines.xhtml

Table S1. Search strategy in Pubmed

No	Search type
#1	Catheter-related thrombosis [Title/Abstract]
# 2	Catheter-related thromboembolism [Title/Abstract]
# 3	Peripherally inserted central catheter,-related thrombosis [Title/Abstract]
# 4	Venous thromboembolism [Title/Abstract]
# 5	Venous thrombosis [Title/Abstract]
# 6	Vein thrombosis [Title/Abstract]
# 7	Catheter-related thrombosis [MeSH Terms]
# 8	Catheter-related thromboembolism [MeSH Terms]
# 9	Peripherally inserted central catheter-related thrombosis [MeSH Terms]
# 10	Venous thromboembolism [MeSH Terms]
# 11	Venous thrombosis [MeSH Terms]
# 12	Vein thrombosis [MeSH Terms]
# 13	# 1 OR # 2 OR # 3 OR # 4 OR # 5 OR # 6 OR # 7 OR # 8 OR # 9 OR # 10 OR # 11 OR # 12
# 14	((clinical practice guidelineline [Title/Abstract]) OR (guidance [title / Abstract]) OR (Evidence-Based Practice [Title/Abstract]) Filters: from 2017-2022
# 15	# 13 AND # 14

For peer review only - http://bmjopen.bmj.com/site/about/guidelines.xhtml

Table S2. Includes the basic characteristics table of the guideline

Numbe	Name	Year of	Publishing agency	Nature of	Subject	Influence
1	Chinese expert consensus on Prevention and treatment of Venous Thrombosis associated with	2020	the China Branch of International Union of	Clinical guideline	Prevention of catheter-related	1.655
2	Venous Thromboembolism Prophylaxis and Treatment in Patients With Cancer: ASCO	2019	American Society of Clinical Oncology	Evidence -based	Prevention of cancer-related thrombosis	44.544
3	guidelinelines for the prevention and treatment of thrombosis diseases in China	2018	Chinese Medical Association	Clinical guideline	Diagnosis and prevention of	1. 056
4	SEOM clinical guidelineline of venous thromboembolism (VTE) and cancer	2019	Spanish Society of Medical Oncology	Evidence -based	Prevention and treatment of cancer-	3. 405
5	Cancer Associated Venous Thromboembolic Disease	2021	National Comprehensive	Evidence -based	Evaluation, prevention and	11.908

For peer review only - http://bmjopen.bmj.com/site/about/guidelines.xhtml

Table S2 (continued) Includes the basic characteristics table of the guideline

Name	Year of	Publishing agency	Nature of	Subject	Influence
American Society of Hematology 2020 guidelines for management of venous thromboembolism: optimal management of [illegible]	2020	American Society of Hematology	Evidence-based guideline	Drug prevention strategy of venous thrombosis	6. 799
2019 international clinical practice guidelines for the treatment and prophylaxis of venous thromboembolism in patients with cancer	2019	International Initiative on Thrombosis and Cancer	Evidence-based guideline	Prevention and treatment of cancer-related thrombosis	41. 316
Venous thromboembolism in over 16s: reducing the risk of hospital-acquired deep	2018	National Institute of Health and Clinical	Clinical guideline	Prevention and treatment of	6. 998
Infusion Therapy Standards of Practice	2021	Infusion nurse	Clinical	Methods and	–

For peer review only - http://bmjopen.bmj.com/site/about/guidelines.xhtml

Num	6	7	8	9

Note: American Society of Clinical Oncology (ASCO); Spanish Society of Medical Oncology (SEMO); National Comprehensive Cancer Network (NCCN); American Society Hematological(ASO); International Initiative on Thrombosis and Cancer(ITAC-CME); National Institute for Health and Clinical Excellence(NICE); Infusion Nursing(INS).

For peer review only

Table S3. specific standardized scores in each area of the guideline

Number	Standardized score by field (%)						The number of standardized scores greater than 60%	RECOMMENDED Grade
	Scope and Purpose	Participants	rigorism	Clarity	Application	independence		
1	61.11	63.89	46.88	80.56	47.92	54.17	3	B
2	77.78	75.00	79.17	75.00	64.59	70.83	6	A
3	83.33	72.22	78.13	91.67	54.17	79.17	5	B
4	75.00	58.33	65.63	63.89	85.42	58.33	4	B
5	88.89	69.44	69.79	94.44	41.29	87.50	5	B
6	80.56	75.00	85.42	91.67	77.08	79.17	6	A
7	88.89	69.44	72.92	77.78	66.67	83.33	6	A
8	77.78	52.78	67.71	80.56	93.75	62.50	5	B
9	77.77	75.00	63.54	72.22	45.83	62.50	5	B

Mean Score	79.01	67.90	69.91	80.87	64.08	70.83	-	-

Table S4. Summary of recommendations included in the guidelines

Items		Source guideline	Recommendation
Risk screening	Thrombosis assessment tool	1、2、3、5、7、8、9	1. The main risk factors assessed included catheterization factors (catheter materials, placement techniques, location and type of catheter, presence of other catheters or catheters, etc.); potential co-disease factors (antiangiogenic therapy, hormone therapy, parenteral nutrition and radiotherapy, tumor and trauma and complex surgery) Patient factors (age, gender, family history or medical history of venous thrombosis, hypercoagulation status and abnormal coagulation gene).<br>2. At present, there is no scale dedicated to CRT risk assessment, and it is recommended to use mature VTE risk assessment scale in clinical practice. Modified Khorana score and Wells scale should be used in CRT risk assessment. Padua scale is recommended for

For peer review only

			medical patients and IMPED and SAVED scales are recommended for patients with multiple myeloma.
	Timing of thrombosis evaluation	1、2、3、5、8、9	3. Admission / pre-catheterization / maintenance catheter / symptoms and signs / catheter extraction / discharge / anticoagulant therapy.
Preventions	Identify as soon as possible	1、3、9	4. Pay close attention to CRT related symptoms and signs such as pain, swelling and skin pruritus on the side of catheterization, and monitor blood routine and coagulation function in time.
	Stay alert.	2、4	5. Correct the wrong operation in time through quality control, conduct regular staff meetings to summarize and feedback on the recent work, and formulate staff briefing warnings to remind other personnel.
	Timely report	1、2、4、	6. Pay attention to high-risk patients and timely report adverse reactions to prevent them.

For peer review only - http://bmjopen.bmj.com/site/about/guidelines.xhtml

	8	
Classified prevention	1、2、3、5、8	7. To evaluate the risk factors and existing symptoms and signs of CRT in patients, and target to prevent and manage them according to different factors and clinical manifestations.<br>8. According to the risk benefit model, individualized drug prevention and physical prevention.
Multi-disciplinary prevention	1、2、4、5、6、9	9. To construct a venous access management team composed of doctors, nurses, pharmacists, medical technologists, evidence-based medicine experts and managers to improve the scientific and comprehensive of CRT prevention and treatment.
Physical prevention	1、2、5、8、9	10.Patients who stay in bed for a long time or have limited activity can massage their lower extremities with intermittent inflatable pressure pump to prevent thrombosis for 1 to 2 weeks. The skin condition of the patients should be evaluated before and after use.<br>11.To carry out daily activities as soon as possible, CVC catheter side should carry out early functional exercise, such as repeatedly clenching , loose, chest breathing and abdominal breathing, etc.<br>12.Replenish the patient with water in time to relieve

For peer review only

			dehydration.<br>13.For patients at risk of post-thrombosis syndrome, elastic socks are not recommended.
Preventions	Drug prevention	2、4、5、6、8	14.After evaluating the risk of bleeding and thrombosis, unfractionated heparin, low molecular heparin, vitamin K antagonist or direct oral anticoagulant drug can be used for prevention. After taking the drug, the laboratory indexes of the patients should be monitored, INR should be monitored in patients with Warfarin, and the arm circumference of the catheterization side should be measured regularly to ask if the patient has bleeding related manifestations.
	Common catheter-related thrombosis prevention	1、2、3、4、5、6、7、8、9	15. When CVC intubation, the catheter with small diameter, less lumen and less trauma should be selected. If conditions permit, the ratio of the outer diameter of the catheter to the diameter of the catheterized vein should be less than 45%.<br>16. Ultrasonic guided intubation should be used in CVC catheterization, the elbow and other frequently moving positions should be avoided when catheterization, upper elbow with low activity, large

For peer review only

			diameter veins, fast blood flow rate and more blood flow should be selected. The catheter tip should be the lower third of the superior vena cava or the intersection of chambers<br>17. At present, CVC is not removed except for complications such as any longer indwelling CVC, blockage or dysfunction of CVC catheter, not within normal range, complicated with CRT-related blood flow infection, and so on.<br>18. It is recommended that CVC should be treated with anticoagulant therapy for 1 or 2 weeks before extubation, and then extubation should be anticoagulation at least 3 months after extubation. If CVC functions are normal and decide to continue to use it, even if the anticoagulant period has reached 3 months, it is necessary to continue anticoagulation until the CVC catheter is completely removed.
Knowledge training	medical staff	2、9	20. Learn CRT diagnosis, prevention related knowledge, master the correct treatment process, participate in bedside rounds and medical record discussions, participate in other healthcare organizations and expert academic exchanges

For peer review only

Patient	2、3、9	21. Inform patients of disease-related knowledge, such as physical preventive measures, related symptoms and signs, side effects of drugs, emergency management, etc.
Multi-disciplinary participation	2、3、5、7	22. Multi-disciplinary sharing, active organization of book exchange meetings, regular academic sharing.

Table S5. Summary of views on preventive measures included in the guidelines

Number	Advice and recommendation	Do not recommend comments
1	In order to reduce the injury caused by CVC, the catheter with small diameter and small number of lumens should be selected as much as possible; ultrasonic guidance should be used to reduce the number of catheterization in CVC catheterization; the elbow joint that often moves should be avoided in CVC catheterization and the upper elbow should be selected; The middle and lower 1/3 of superior vena	Prophylactic use of anticoagulant or thrombolysis drugs is not recommended. Chest enhancement CT and three-

For peer review only - http://bmjopen.bmj.com/site/about/guidelines.xhtml

	cava and the intersection of chambers and chambers is the best intubation choice at the tip of CVC; early active after CVC catheterization. The indexes of blood routine and coagulation function and related symptoms and signs were monitored in time after the use of anticoagulant drugs.	dimensional lung imaging are not recommended to screen CRT patients for pulmonary embolism.
2	The prevention of CRT in cancer patients can be based on the combination of drug prevention and mechanical prevention; drug prevention can use heparin (low molecular weight heparin, unfractionated heparin) drugs; CRT high risk patients should be evaluated according to the actual situation after they can decide the time to start anticoagulation; CRT high risk patients (major surgery patients and cancer patients) should be given health education.	Mechanical prophylaxis is not recommended as a single measure of VTE prevention; anticoagulant is not recommended to improve the survival rate of cancer patients without VTE symptoms.
3	CVC function is normal, the catheter can be retained; all patients should be treated with anticoagulant	For patients with deep venous

For peer review only - http://bmjopen.bmj.com/site/about/guidelines.xhtml

	treatment after removing CVC for at least 3 months; if the anticoagulant time is more than 3 months after CVC catheterization, anticoagulation should continue until the catheter is removed.	thrombosis at risk of post-thrombotic syndrom , routine use of elastic socks is not recommended.
4	Risk factors for CRT formation include catheter factors (catheter material, placement techniques, location and type of catheter) and potential co-disease factors (antiangiogenic therapy, hormone therapy, parenteral nutrition and radiotherapy), as well as patient factors (age, sex and thrombosis-related diseases) The first choice of prevention after CVC intubation is to use low molecular heparin anticoagulant therapy for 3-6 months, if CVC has not been removed for more than 3 months, then low molecular heparin is used for anticoagulation indefinitely; direct oral anticoagulant DOAC is also recommended; if anticoagulant therapy is ineffective or anticoagulant failure, CVC should be removed after 5 or 7 days of anticoagulation.	Except for CVC related infection, unsuitable anticoagulant, poor anticoagulant effect and decision to remove CVC, CVC transfer is not recommended..

For peer review only - http://bmjopen.bmj.com/site/about/guidelines.xhtml

5	If the CVC function is normal, you can continue to use it. When CVC is retained, it is recommended that anticoagulant therapy be carried out for CRT formation; if CVC is no longer needed, anticoagulant therapy should be performed 5 or 7 days before CVC removal; if CVC related infection, CRT or worsening symptoms, CVC should be removed when there is anticoagulant contraindication, and the risk of thrombosis should be taken into account, and the anticoagulant treatment should be decided according to the specific situation. In order to prevent CRT, cancer patients should be treated with anticoagulant therapy for at least 3 months, or anticoagulant therapy should be performed throughout the catheterization period; medical personnel should inform patients of the risks and benefits of anticoagulant, and determine the duration of treatment after being known to the patient.	Prophylactic anticoagulant therapy for patients with CVC intubation is not recommended.
6	The use of anticoagulant drugs alone in the treatment of symptomatic CRT in children can minimize complications and do not need to be pulled out of the catheter; for non-functional or no longer used CVC,	Multiple CVC intubation is not recommended; routine use of

For peer review only - http://bmjopen.bmj.com/site/about/guidelines.xhtml

	it is recommended that anticoagulant therapy remove CVC a few days later.	elastic socks is not recommended.
7	The right jugular vein is the first choice for CVC catheterization, in which the intersection of the right atrium and the superior vena cava is the best position for the distal tip of the catheter; for patients with PICC catheterization, the implantation port should be used; for symptomatic CRT, anticoagulant therapy should be performed for at least 3 months as long as CVC is in situ; there is no guideline to compare the anticoagulant effects of low molecular weight heparin, DOAC and vitamin K antagonists If the CVC catheter is unblocked the position of the catheter is normal, no infection occurs, and other symptoms disappear during anticoagulant, CVC can remain in place.	Undifferentiated anticoagulant therapy is not recommended for prevention of CRT formation.
8	When considering whether to use drugs to prevent CRT, it is necessary to analyze the risks and benefits of anticoagulant; LMWH or DOAC can be selected for CRT drug prevention; patients should be educated about VTE risk factors and related	Routine thrombosis prevention is not recommended in all patients;

For peer review only - http://bmjopen.bmj.com/site/about/guidelines.xhtml

	symptoms and signs.	thrombosis prevention time is not recommended for less than 12 weeks.
9	The risk of CRT should be assessed before CVC intubation; the appropriate intubation location and catheter type should be selected before catheterization; the selected veins should be observed and measured by ultrasound in advance before PICC intubation, and the catheter with the ratio of catheter diameter to vein diameter less than 45% should be preferred; the tip of CVC should be located at 1/3 of superior vena cava or the junction of vena cava and atrial vein; and the circumference of upper arm should be measured before insertion of PICC to assess the presence of edema and deep venous thrombosis. When CVC is located correctly, functionally normal and has no sign of infection, CVC does not need to be removed even if deep venous thrombosis exists.	It is not recommended that CVC intubation be located in the anterior cubital fossa and prophylactic anticoagulant therapy is not recommended.

For peer review only

Revised Standards for Quality Improvement Reporting Excellence (SQUIRE 2.0)
September 15, 2015

Text Section and Item Name	Section or Item Description
**Notes to authors**	. The SQUIRE guidelines provide a framework for reporting new knowledge about how to improve healthcare<br>. The SQUIRE guidelines are intended for reports that describe systemlevel work to improve the quality, safety, and value of healthcare, and used methods to establish that observed outcomes were due to the intervention(s).<br>. A range of approaches exists for improving healthcare. SQUIRE may be adapted for reporting any of these.<br>. Authors should consider every SQUIRE item, but it may be inappropriate or unnecessary to include every SQUIRE element in a particular manuscript.<br>. The SQUIRE Glossary contains definitions of many of the key words in SQUIRE.<br>. The Explanation and Elaboration document provides specific examples of well-written SQUIRE items, and an in-depth explanation of each item.<br>. Please cite SQUIRE when it is used to write a manuscript.
**Title and Abstract**	
**1. Title**	Quality evaluation and content analysis of clinical practice guidelines related to central venous catheter-related thrombosis prevention
**2. Abstract**	Background: Central venous catheter (CVC)operation is convenient and relatively safe, and it is widely used in clinical practice. Catheter-associated venous thrombosis is one of the common complications after CVC catheterization. Several clinical practice guidelines(CPGs) for Catheter-related thrombosis (CRT) prevention have been published, but their quality is unclear.<br>Objective: To evaluate the quality and analyze the content of clinical practice guidelines regarding central venous catheter-related thrombosis to provide evidence for formulating an evidence-based practice protocol and a risk assessment scale to prevent it.<br>Methods: Clinical practice guidelines regarding central venous catheter-related thrombosis prevention were searched in guidelines websites and databases such as National Institute for Health and Care Excellence, Up to date, Pubmed, Web of Science, CNKI, and others. The included guidelines were evaluated using AGREE II.<br>Results: A total of nine guidelines were included, and the quality grade results showed that three were at A-level and six were at B-level. The included

For peer review only - http://bmjopen.bmj.com/site/about/guidelines.xhtml

	guidelines are mainly recommended the prevention measure of central venous catheter-related thrombosis from three aspects: risk screening, prevention strategies, and knowledge training, with a total of 22 suggestions were recommended.<br>Conclusion: The overall quality of the guidelines is high, but there are few preventive measures for central venous catheter-related thrombosis involved in the guidelines. All preventive measures have not been systematically integrated and evaluated, and no risk assessment scale dedicated to this field has been recommended. Therefore it is urgent to develop an evidence-based practice protocol and a risk assessment scale to prevent it.
**Introduction**	*Why did you start?*
**3. Problem Description**	Catheter-related venous thrombosis is a common complication of central venous catheters in chemotherapy, haemodialysis, nutritional support, and surgical support, and several clinical practice guidelines on CRT prevention have been published, but their quality is unclear. It makes sense to evaluate the quality and analyze the content of the guide
**4. Available knowledge**	assessment and taking measurements early to prevent patients at high risk of CRT risk are essential to ensure the safety of patients. Clinical Practice guideline is formulated by clinical workers after systematically integrating and evaluating relevant research and clinical experience according to evidence-based concept to a certain common symptom, disease or problem, to provide guiding suggestions for clinical workers and patients, and scientifically promote the application of evidence in clinical practice. At present, several clinical practice guidelines about CRT prevention have been published, but their quality is unclear. Therefore, through the systematic retrieval, screening and reading of clinical practice guidelines related to CRT prevention, and the quality evaluation and content analysis of the guidelines, this review was expected to provide scientific basis for the development of evidence-based practice protocols and risk assessment scales for CRT.

For peer review only

**5. Rationale**	We searched guideline websites and databases for clinical practice guidelines on central venous catheter-associated thrombosis, and assessed the included guidelines using AGREEII.
**6. Specific aims**	To evaluate the quality and analyze the content of clinical practice guidelines regarding central venous catheter-related thrombosis to provide evidence for formulating an evidence-based practice protocol and a risk assessment scale to prevent it.
**Methods**	*What did you do?*
**7. Context**	Central venous catheter (CVC)operation is convenient and relatively safe, and it is widely used in clinical practice. Catheter-associated venous thrombosis is one of the common complications after CVC catheterization. Several clinical practice guidelines(CPGs) for Catheter-related thrombosis (CRT) prevention have been published, but their quality is unclear.
**8. Intervention(s)**	Clinical practice guidelines regarding central venous catheter-related thrombosis prevention were searched in guidelines websites and databases such as National Institute for Health and Care Excellence, Up to date, Pubmed, Web of Science, CNKI, and others. The included guidelines were evaluated using AGREE II.
**9. Study of the Intervention(s)**	The AGREE II scale, a widely used tool for evaluating the quality of the guideline, was used to evaluate the included guidelines(22). It includes 6 domains, i.e. scope and purpose, rigorism, independence, clarity, application, and independence of writing, with 23 items.
**10. Measures**	Two researchers who had systematically trained the use of the AGREE II scale were selected to conduct relevant research on the guidelines using the AGREE II scale and evaluate their quality, including a total of 6 areas, including scope and purpose, study rigor, independence, clarity of discussion, application, and independence of writing, a total of 23 items, and SPSS22.0 was used to count the scores of two investigators in each field of the guidelines and calculate the relevant results
**11. Analysis**	SPSS22.0 was used to count the scores of the two researchers in each field of the guideline, and the Intraclass Correlation Coefficient (ICC) of the data was calculated. According to the results, the consistency of the two researchers' evaluation of the guideline was tested. ICC < 0.4 indicated poor consistency, $0.40 \leq 0.59$ indicated that the consistency is general, $0.60 \leq 0.74$ indicated good consistency, and > 0.74 indicated that the consistency is very good.
**12. Ethical Considerations**	Competing interests： The authors declare that they have no conflicts of interest.<br>Ethical considerations：Not involved
**Results**	*What did you find?*
**13. Results**	a. A total of 2092 articles were retrieved, and 571 articles were removed by software and manual deletion. After read the title and abstract, 1142 articles were excluded. 379 articles were screened according to inclusion and exclusion criteria. 267 articles that did not meet the theme of the article were excluded, and 5 related guidelines were traced through the snowball strategy, and 117 articles were finally selected. Among them, 11 articles were not obtained, and a total of 106 complete articles were obtained. Full-text screened excluded 18 non-evidence-based guidelines issued in individual form, 6 guidelines that had been updated, and 73 guidelines that did not include CRT treatment, prevention, and management. Finally, 9 literatures were selected, including 2 Chinese literatures and 7 English literatures.<br>b. The nine guidelines were from the American Society of Clinical Oncology, the International Initiative on Thrombosis and Cancer, the Chinese Medical Association, the Spanish Medical Oncology Society, the Infusion Nursing Association, and other international authoritative

For peer review only - http://bmjopen.bmj.com/site/about/guidelines.xhtml

	medical associations. Only one of them is a guideline targeted for CRT risk assessment, prevention, and intervention18. Five guidelines were specifically for venous thrombosis prevention in cancer patients, and the content related to CRT was only part of it. Two of which are guidelines for venous thrombosis of ordinary patients. CRT patients are part of the content, and only one guideline is targeted for clinical infusion therapy. CRT is part of the treatment<br>c. After scoring the nine guidelines with the AGREEII tool, it was found that the recommended A-level guidelines were three(22,23,28). The recommended B-level guidelines are six(14,25–27). An analysis of the data showed that the highest score of clarity in the guideline was 80.87%, followed by scope and purpose (79.01%), independence of the writing (70.74%), rigorism and independence of the guideline (69.91%), participants (67.90%), and application (64.08%). The details are shown in Table S3. After the ICC calculation of the scores of each guideline by two evaluators, it was concluded that the ICC of the two evaluators for the nine guidelines was more than 0.8, and indicated that the consistency among the evaluators is good.<br>d. We summarised the similarities and differences in the guidelines for thromboprophylaxis associated with central venous catheters in a table
**Discussion**	*What does it mean?*
**14. Summary**	a. Key findings：The overall quality of the nine guidelines included is good, and the corresponding advice and suggestions about CRT prevention are provided, but the guidelines still need to be improved in terms of rigorism and independence of writing, participants and applications. At present, there is still a lack of high-quality clinical practice guidelines for CRT prevention, and there is also a lack of dedicated CRT risk assessment tool.<br>b. Particular strengths of the project：NA

For peer review only

**15. Interpretation**	a. Most of these guidelines are only based on the experience of clinical experts for advice and guideline preparation, and lack scientific nature. Most of the remaining guidelines are suitable for cancer patients, mainly involving CRT-related prevention strategies as drug prevention and CVC catheter prophylaxis, but their content is small and dispersed, and the guidelines are limited and lack systematic integrated evaluation.<br>b. Comparison of results with findings from other publications: NA<br>c. Impact of the project on people and systems : NA<br>d. By evaluating the content and quality of these guidelines, it is found that there is currently a lack of high-quality clinical practice guidelines dedicated to CRT prevention at home and abroad, and there is currently a lack of risk assessment scales dedicated to CRT in clinical practice. We believe that it is urgent to develop clinical practice guidelines and risk assessment scales related to CRT prevention based on evidence-based concepts, so as to involve patients in research based on clinical status, reduce the incidence of CRT, and improve the level of CRT detection, management and prevention.<br>e. Costs and strategic trade-offs, including opportunity costs: NA
**16. Limitations**	Although one of the nine guidelines included in this study is a specific guideline on CRT prevention, it does not systematically retrieve relevant research and evidence, and only recommends and compiles guidelines based on the experience of clinical experts, which is lack of scientific. Most of the other guidelines are suitable for cancer patients, and the main prevention strategies related to CRT are drug prevention and CVC catheter prevention, but their contents are less and their views are scattered, the subjects of the guidelines are limited and lack of systematic integrated evaluation. Therefore, the formulation of evidence-based CRT prevention related clinical practice guidelines is helpful to guideline clinical staff to standardize the operation and ensure the treatment quality of patients. At the same time, after analyzing the content of the guideline, it is found that there is no tool dedicated to CRT risk assessment. At present, the guidelines recommend the use of mature VTE risk assessment scale, but this kind of scale does not involve the evaluation of CVC catheter, which is not targeted, which may lead to the bias of measurement. Therefore, the development of a risk assessment scale for CRT is conducive to the accurate selection of appropriate preventive measures and anticoagulant treatment methods, and reduces the injury caused to patients after the occurrence of CRT.
**17. Conclusions**	The overall quality of the nine guidelines included is good, and the corresponding advice and suggestions about CRT prevention are provided, but the guidelines still need to be improved in terms of rigorism and independence of writing, participants and applications. At present, there is still a lack of high-quality clinical practice guidelines for CRT prevention, and there is also a lack of dedicated CRT risk assessment tool. Therefore, there is an urgent need to develop evidence-based CRT prevention

For peer review only

	related clinical practice guidelines and risk assessment tools based on the clinical situation to enable patients to participate in the management of CRT, thereafter to to better manage the CRT.
**Other information**	
**18. Funding**	This work was supported by the Sichuan Science and Technology Program (Grant 2022NSFSC1290), and West China Nursing Discipline Development Special Fund Project, Sichuan University (Grant HXHL21011).

For peer review only

Table 2. Glossary of key terms used in SQUIRE 2.0. This Glossary provides the intended meaning of selected words and phrases as they are used in the SQUIRE 2.0 Guidelines. They may, and often do, have different meanings in other disciplines, situations, and settings.

Assumptions
Reasons for choosing the activities and tools used to bring about changes in healthcare services at the system level.

Context
Physical and sociocultural makeup of the local environment (for example, external environmental factors, organizational dynamics, collaboration, resources, leadership, and the like), and the interpretation of these factors ("sense-making") by the healthcare delivery professionals, patients, and caregivers that can affect the effectiveness and generalizability of intervention(s).

Ethical aspects
The value of system-level initiatives relative to their potential for harm, burden, and cost to the stakeholders. Potential harms particularly associated with efforts to improve the quality, safety, and value of healthcare services include opportunity costs, invasion of privacy, and staff distress resulting from disclosure of poor performance.

Generalizability
The likelihood that the intervention(s) in a particular report would produce similar results in other settings, situations, or environments (also referred to as external validity).

Healthcare improvement
Any systematic effort intended to raise the quality, safety, and value of healthcare services, usually done at the system level. We encourage the use of this phrase rather than "quality improvement," which often refers to more narrowly defined approaches.

Inferences
The meaning of findings or data, as interpreted by the stakeholders in healthcare services – improvers, healthcare delivery professionals, and/or patients and families

Initiative
A broad term that can refer to organization-wide programs, narrowly focused projects, or the details of specific interventions (for example, planning, execution, and assessment)

Internal validity
Demonstrable, credible evidence for efficacy (meaningful impact or change) resulting from introduction of a specific intervention into a particular healthcare system.

Intervention(s)
The specific activities and tools introduced into a healthcare system with the aim of changing its performance for the better. Complete description of an intervention includes its inputs, internal activities, and outputs (in the form of a logic model, for example), and the mechanism(s) by which these components are expected to produce changes in a system's performance.

Opportunity costs

For peer review only

Loss of the ability to perform other tasks or meet other responsibilities resulting from the diversion of resources needed to introduce, test, or sustain a particular improvement initiative

Problem

Meaningful disruption, failure, inadequacy, distress, confusion or other dysfunction in a healthcare service delivery systemthat adversely affects patients, staff, or the system as a whole, or that prevents care from reaching its full potential

Process

The routines and other activities through which healthcare services are delivered

Rationale

Explanation of why particular intervention(s)were chosen and why it was expected to work, be sustainable, and be replicable elsewhere.

Systems

The interrelated structures, people, processes, and activities that together create healthcare services for and with individual patients and populations. For example, systems exist from the personal self-care system of a patient, to the individual provider-patient dyad system, to the microsystem, to the macrosystem, and all the way to the market/social/insurance system. These levels are nested within each other.

Theory or theories

Any “reason-giving” account that asserts causal relationships between variables (causal theory) or that makes sense of an otherwise obscure processor situation (explanatory theory). Theories come in many forms, and serve different purposes in the phases ofimprovementwork. It is important to be explicit and well-founded about any informal and formal theory (or theories) that are used.

For peer review only

Appraising the quality standard of clinical practice guidelines related to central venous catheter-related thrombosis prevention: a systematic review of reviews

Journal:	*BMJ Open*
Manuscript ID	bmjopen-2023-074854.R1
Article Type:	Original research
Date Submitted by the Author:	18-Jan-2024
Complete List of Authors:	Zhang, Jing; Sichuan University<br>Wu, Yongya; Sichuan University<br>Zhang, Shuai; Sichuan University<br>Yao, Wenmo; Sichuan University<br>Bu, Faqian; Sichuan University<br>Wang, Aoxue; Sichuan University<br>Hu, Xiuying; Sichuan University<br>Wang, Guan; Sichuan University
**Primary Subject Heading**:	Cardiovascular medicine
Secondary Subject Heading:	Cardiovascular medicine, Haematology (incl blood transfusion), Oncology
Keywords:	Thromboembolism < CARDIOLOGY, Systematic Review, VASCULAR MEDICINE, Anaesthesia in oncology < ANAESTHETICS

SCHOLARONE™
Manuscripts

I, the Submitting Author has the right to grant and does grant on behalf of all authors of the Work (as defined in the below author licence), an exclusive licence and/or a non-exclusive licence for contributions from authors who are: i) UK Crown employees; ii) where BMJ has agreed a CC-BY licence shall apply, and/or iii) in accordance with the terms applicable for US Federal Government officers or employees acting as part of their official duties; on a worldwide, perpetual, irrevocable, royalty-free basis to BMJ Publishing Group Ltd ("BMJ") its licensees and where the relevant Journal is co-owned by BMJ to the co-owners of the Journal, to publish the Work in this journal and any other BMJ products and to exploit all rights, as set out in our licence.

The Submitting Author accepts and understands that any supply made under these terms is made by BMJ to the Submitting Author unless you are acting as an employee on behalf of your employer or a postgraduate student of an affiliated institution which is paying any applicable article publishing charge ("APC") for Open Access articles. Where the Submitting Author wishes to make the Work available on an Open Access basis (and intends to pay the relevant APC), the terms of reuse of such Open Access shall be governed by a Creative Commons licence – details of these licences and which Creative Commons licence will apply to this Work are set out in our licence referred to above.

Other than as permitted in any relevant BMJ Author's Self Archiving Policies, I confirm this Work has not been accepted for publication elsewhere, is not being considered for publication elsewhere and does not duplicate material already published. I confirm all authors consent to publication of this Work and authorise the granting of this licence.

For peer review only

Appraising the quality standard of clinical practice guidelines related to central venous catheter-related thrombosis prevention: a systematic review of reviews

Jing Zhang[1], Yongya Wu[1], Shuai Zhang[1], Wenmo Yao[1], Faqian Bu[1], Aoxue Wang[1], Xiuying Hu[1], Guan Wang[1]

[1] Innovation Center of Nursing Research, Nursing Key Laboratory of Sichuan Province, State Key Laboratory of Biotherapy and Cancer Center, West China Hospital, Sichuan University /West China School of Nursing, Sichuan University, Chengdu 610041, China.

Correspondence to

Dr Guan Wang; guan8079@163.com

Dr. Xiuying Hu; huxiuying@scu.edu.cn

For peer review only

Abstract:

Background: Central venous catheter (CVC)operation is convenient and relatively safe, and it is widely used in clinical practice. Catheter-associated venous thrombosis is one of the common complications after CVC catheterization. Several clinical practice guidelines(CPGs) for Catheter-related thrombosis (CRT) prevention have been published, but their quality is unclear.

Objective: To evaluate the quality and analyze the content of clinical practice guidelines regarding central venous catheter-related thrombosis to provide evidence for formulating an evidence-based practice protocol and a risk assessment scale to prevent it.

Methods: Clinical practice guidelines regarding central venous catheter-related thrombosis prevention were searched in guidelines websites and databases such as the National Institute for Health and Care Excellence, Up to Date, Pubmed, Web of Science, CNKI, and others. The included guidelines were evaluated using AGREE II.

Results: A total of nine guidelines were included, and the quality grade results showed that three were at A-level and six were at B-level. The included guidelines mainly recommended the prevention measure of central venous catheter-related thrombosis from three aspects: risk screening, prevention strategies, and knowledge training, with a total of 22 suggestions being recommended.

Conclusion: The overall quality of the guidelines is high, but there are few preventive measures for central venous catheter-related thrombosis involved in the guidelines. All preventive measures have not been systematically integrated and evaluated, and no risk assessment scale dedicated to this field has been recommended. Therefore it is urgent to develop an evidence-based practice protocol and a risk assessment scale to prevent it.

Keywords: Central venous catheter; Catheter-related thrombosis; Venous thromboembolism; Clinical practice guidelines; Prevention

Strengths and limitations of this study

- A comprehensive and systematic review of CVC-related clinical guidelines was conducted, and two quality evaluation tools were used to comprehensively and rigorously evaluate the quality of CVC-related clinical guidelines

For peer review only - http://bmjopen.bmj.com/site/about/guidelines.xhtml

- This review clarifies the CVC guidelines with high evidence strength and the highest clinical applicability and provides a reference for clinical medical personnel to select the best guidelines
- The evidence in the guidelines included in this study is mainly from developed countries and some developing countries, and other countries need to be cautious when interpreting the results of this study

For peer review only

Introduction

The central venous catheter is a catheter inserted through the proximal central vein or peripheral vein, with the catheter tip located at one-third of the junction between the superior vena cava and the right atrium(1). The CVC includes subclavian vein/ jugular vein/femoral vein puncture catheters, peripherally inserted central catheter(PICC), and implantable venous access port(2). It has many advantages, such as convenient and safe operation, rapid establishment of intravenous access, reduction of puncture times, and infusion of irritating liquid; therefore it has been widely used in chemotherapy, hemodialysis, nutrition support, and surgery support(3–6).

Catheter-related thrombosis(7) refers to the formation of blood clots on the outer wall or inner wall of a catheter. CRT is one of the common complications after catheterization of CVC, which can lead to unplanned extubation and pulmonary embolism etc, affecting the progress of treatment and increasing the cost of treatment(8–11). The risk factors of CRT could be categorized into patient-related, disease/treatment-related, and catheter-related factors. Patient-related factors include sex, advanced age, long-term bedridden, venous thromboembolism history, acquired or hereditary blood hypercoagulability state, etc(12–14). Diseases/treatment-related factors include the grade of cancer, chemotherapy, radiotherapy, parenteral nutrition support, surgical trauma or excessive bleeding, etc(15). Catheter-related factors include catheter material, catheter-to-vein ratio, puncture times, length of catheter indwelling, the location of the catheter tip, etc(16).

For peer review only - http://bmjopen.bmj.com/site/about/guidelines.xhtml

CRT has the characteristics of high incidence, early onset, and clinically silent symptoms(17). The clinical manifestations of CRT are as follows: deep venous thrombosis(18) (DVT), superficial thrombophlebitis, asymptomatic thrombosis, and thrombotic catheter failure. The study reported CRT incidence ranged markedly from 1.9 to 73.0%(5,9,16,17,19). It could happen as early as the first day after catheterization(17,20), the average time of occurrence was within 2 weeks after catheterization(5,11,21,22). However, the early symptoms and signs were silent, only a few patients showed clinical symptoms. Due to the limited medical resources, vascular surgery in most medical institutions in China can not monitor the occurrence of CRT in time, and can not correctly prevent and deal with CRT, increasing of hospitalization time, resulting in irreversible effects, and affecting the quality of follow-up treatment of patients.

Therefore, assessment and taking measurements early to prevent patients at high risk of CRT risk are essential to ensure the safety of patients. Clinical Practice guideline is formulated by clinical workers after systematically integrating and evaluating relevant research and clinical experience according to evidence-based concepts to a certain common symptom, disease, or problem, to provide guiding suggestions for clinical workers and patients, and scientifically promote the application of evidence in clinical practice(23). At present, several clinical practice guidelines about CRT prevention have been published, but their quality is unclear. Therefore, through the systematic retrieval, screening, and reading of clinical practice guidelines related to CRT prevention, and the quality evaluation and content analysis of the guidelines, this review was expected to provide a scientific basis for the development of evidence-based practice protocols and risk assessment scales for CRT.

Information and methods

Search strategy

Search databases

We searched both English and Chinese databases, including Pubmed, Embase,

For peer review only - http://bmjopen.bmj.com/site/about/guidelines.xhtml

Cochrane Library, Web of Science, CNKI, Wanfang, VIP, and the Chinese Biomedical Literature Database from January 1st, 2017 to March 26th, 2022. In addition, the relevant websites of the guideline, including to date, National Institute for Health and Care Excellence (NICE), and Medline, were searched. The manual search of relevant studies and bibliography was also adopted.

Search strategies

The literature retrieval adopted a strategy of the combination of MeSH terms and free words. The MeSH terms and synonyms of catheter-related thrombosis and clinical practice guidelines were searched in English and Chinese databases. Take Pubmed as the retrieval representative of the English database. The search strategy is shown in Table S1.

Inclusion and exclusion criteria

The inclusion criteria were (1) guidelines including the contents of treatment, prevention, or management of CRT; (2) guidelines published or updated after January 1st,2017; and (3) guidelines published in English or Chinese. The exclusion criteria were (1) guidelines developed by expert consensus exclusively; (2) incomplete guidelines that are in the process of development; and (3) the translation or reinterpreting of the guidelines.

Guideline screening and data extraction

The literature was searched by two researchers who had received systematic literature retrieval and training. The searched literature was imported into Endnote X9 software and removed the duplicates. Then, two researchers independently read the title and abstracts for preliminary screening to exclude unrelated articles. Subsequently, two reviewers read the full text carefully and screened it again according to the established criteria for inclusion and exclusion. In case of disagreement, the third party will be consulted before determining. Finally, a predefined data extraction table was used to extract the data from the included studies.

Quality appraisal

For peer review only - http://bmjopen.bmj.com/site/about/guidelines.xhtml

The AGREE II scale(24), a widely used tool for evaluating the quality of the guideline, was used to evaluate the included guidelines. It includes 6 domains, i.e. scope and purpose, rigorism, independence, clarity, application, and independence of writing, with 23 items. Each item has a maximum score of seven and a minimum of one. Seven indicates that the evaluator is fully satisfied with the area covered by the entry, and one indicates that the evaluator is completely dissatisfied with the field. The higher the evaluator is satisfied with the area covered by the guideline, the higher the scores. The evaluator should score the satisfaction degree of the guideline according to the actual situation to ensure the fairness of the guideline. Three researchers who systematically trained the use methods of the AGREE II scale were selected to evaluate the guideline to ensure that the researchers had a full understanding and consistent understanding of the items and to reduce the biases of evaluation. If there is a dispute over an item, experts in the relevant field would be consulted to resolve it. The standard rate of the three researchers in each field of the guideline was calculated, and the systematic evaluation content analysis and summary of the guideline were carried out based on the evidence-based concept. Level A recommended the guideline, indicating that most entries in the guideline scored ≥ 5, and its standardized percentage was≥ 60%. Level B was recommended after modification, and indicated that the standard rate in more than three areas was 30-60%. Level C indicated that the guideline was not recommended, and indicated that the standardized rate in three or more areas was less than 30%.

To evaluate the quality and applicability of each recommendation, improve the comparability of the results, and increase the effectiveness of the evaluation process, the AGREE REX scale(25) was used to evaluate the included guidelines, and three researchers who had been systematically trained in the use of the AGREE REX scale evaluated the guidelines respectively, and the scores of the three researchers in each area of the guidelines were standardized to calculate, and the guidelines were systematically evaluated and content analyzed and summarized based on the evidence-based concept. Referring to the recommendations of the AGREE REX manual, the guidelines define an overall score of >70% as high quality, an overall score of <30% as low quality, and others as moderate quality.

For peer review only - http://bmjopen.bmj.com/site/about/guidelines.xhtml

Statistical methods

SPSS22.0 was used to count the scores of the three researchers in each field of the guideline, and the Intraclass Correlation Coefficient (ICC) of the data was calculated. According to the results, the consistency of the two researchers' evaluation of the guideline was tested. ICC < 0.4 indicated poor consistency, 0.40 ≤ 0.59 indicated that the consistency is general, 0.60 ≤ 0.74 indicated good consistency, and > 0.74 indicated that the consistency is very good.

Patient and public involvement

No patients or members of the public were involved in the development of the research question, nor the design or the conduct of the study, nor the interpretation and writing up of results.

Result

Guideline retrieval results

A total of 2092 articles were retrieved, and 571 articles were removed by software and manual deletion. After reading the title and abstract, 1142 articles were excluded. 379 articles were screened according to inclusion and exclusion criteria. 267 articles that did not meet the theme of the article were excluded, 5 related guidelines were traced through the snowball strategy, and 117 articles were finally selected. Among them, 11 articles were not obtained, and a total of 106 complete articles were obtained. Full-text screened excluded 18 non-evidence-based guidelines issued in individual form, 6 guidelines that had been updated, and 73 guidelines that did not include CRT treatment, prevention, and management. Finally, 9 literature were selected, including 2 Chinese literature and 7 English literature. The specific screened process is shown in Figure S1.

Basic characteristics of inclusion in the guideline

For peer review only - http://bmjopen.bmj.com/site/about/guidelines.xhtml

For peer review only

The nine guidelines were from the American Society of Clinical Oncology, the International Initiative on Thrombosis and Cancer, the Chinese Medical Association, the Spanish Medical Oncology Society, the Infusion Nursing Association, and other international authoritative medical associations. Only one of them is a guideline targeted for CRT risk assessment, prevention, and intervention(20). Five guidelines were specifically for venous thrombosis prevention in cancer patients(15,26–29), and the content related to CRT was only part of it. Two of which are guidelines for venous thrombosis of ordinary patients(26,30). CRT patients are part of the content, and only one guideline is targeted for clinical infusion therapy(30). CRT is part of the treatment (see Table S2).

Results of AGREE II and AGREE REX

evaluations The included guidelines used AGREE II and AGREE-REX to evaluate the quality of methodologies and recommendations, respectively. After scoring the 9 guidelines on the AGREE II scale, it was found that 3 guidelines(26,31,32) were recommended as A and 6 guidelines(27,29,33–36) were recommended as B, as shown in Table S3. The average score in all areas was greater than 60%. The ICC of the scores of the three reviewers for each domain of each guideline was greater than 0.8, indicating that the agreement among the reviewers was good. Specifically, Area 1 ("Scope and Purpose") addresses the general scope and purpose of CPGS, the specific clinical problems that need to be addressed, and the target population. The average score was 66.67%, of which 8 articles scored more than 60% and 5 papers scored more than 70%. Area 2 ("Participants") reflects whether the normative process includes input and participation from relevant stakeholders. The average score was 60.70%, and 6 of the articles scored above the average score. In terms of "rigor" (area 3), the average score was 61.88%, with 7 guidelines scoring above the average. Guideline 4(35) has the lowest scores in the three areas of scope and purpose, who is involved, and rigor, while most of the rest describe the development process, albeit with varying levels of detail. Area 4 ("Clarity") focuses on the specificity and clarity of these items, including the clear articulation of options for health management and the making of key recommendations that are easy to identify. With an average score of 64.81%, there was

For peer review only - http://bmjopen.bmj.com/site/about/guidelines.xhtml

the smallest difference in all guideline scores in this area, even though guideline scores 1, 2, 8, and 9 were all below the average. "Application" (area 5) refers to facilitators and obstacles to the implementation of recommendations within the context of the guidelines, strategies for their implementation, and possible resource implications. The CPGS scored an average of 60.49 % in this area, with 3 scores below 60%. Finally, the sixth area ("independence") aims to ensure that there is a lack of bias in the development of guidelines. The score in this area was relatively good, with a median CPGS of 63.58%, 3 above 70%, and 7 above 60%. Most described funding, potential impact, or associated conflicts of interest. The guidelines scored slightly worse than the others in the areas of participants, rigor of development, applicability, and editorial independence.

After evaluating the nine guidelines according to the AGREE REX scale, the overall scores of guidelines 3(34) and 6(32) were > 70%, which was of high quality, and the overall scores of the other guidelines were ≥ 30%, which was of moderate quality. (see Table S4)The three reviewers agreed well, with an ICC value greater than 0.8, and each guideline scored slightly lower than the implement ability domain in the values and preference domains and clinical practicality. In the field of clinical applicability, guideline 6(32) scored the highest score of 83.33%, with a large score difference between guidelines. guideline 1(33) had the lowest score of 42.59%, as detailed in Table S4.

Comparative analysis of the specific content of CRT preventive measures in the guidelines

After reading all the recommendations and precautions included in the guidelines, it was found that the content of CRT prevention can be mainly divided into three aspects: risk assessment, preventive measures, and health education, and the specific content and methods are shown in Table S5. Due to differences in the topic of the guidelines and the participants of the study, the focus and level of detail of the prevention strategies recommended for CRT vary among the guidelines. Therefore, for analysis and comparison, the relevant contents are summarized in Table S6.

For peer review only - http://bmjopen.bmj.com/site/about/guidelines.xhtml

Discussion

Basic characteristics of CRT Prevention guidelines

The guidelines included in the study are widely used and have high impact factors. Guidelines issued by the China Branch of the International Union of Angiology, for the first time, the preventive measures of CRT were systematically summarized, which filled the gap in this field, but the methodological quality was poor. Guidelines issued by ASCO, SEOM, ASH, NCCN, ITAC-CME(15,26,27,32). This paper mainly expounds on the preventive measures of CRT from two aspects of drug prevention and CVC-related prevention, including the choice of anticoagulant drugs, the time of continuous anticoagulant treatment, and the choice of catheter location; however, these were only a small part of the guideline and the content were scattered in the included guidelines. Five included guidelines were targeted at cancer patients and had certain limitations. Guidelines issued by the Chinese Medical Association and NICE(26,30), mainly put forward some suggestions on how to prevent various types of thrombosis, and the prevention measures for CRT were one of them, including drug prevention of CRT, CVC catheter-related prevention, and physical prevention. The guideline issued by INS described the latest clinical infusion treatment practice standards and specific measures(30). The prevention of CVC-related complications was part of them, which mainly describes how to deal with CRT.

Quality evaluation of the guidelines

To ensure the quality of the guidelines, researchers should follow the principles of professionalism, fairness, evidence-based and transparency when developing the guidelines. As the guidelines included in this study are of good quality and high impact factors, the AGREE II scale is used to evaluate the six areas of the guideline, and it is found that the clarity, scope, and purpose of the guidelines have reached the standard level, while the rigorism and independence, participants and application of the guidelines need to be further improved, the specific analysis was as follows:

rigorism and independence

For peer review only - http://bmjopen.bmj.com/site/about/guidelines.xhtml

In the aspect of rigorism, the expert consensus was released by the China Branch of the International Union of Angiology. The guideline does not systematically retrieve CRT prevention-related research but only summarizes the "experience" of clinical experts, lack of corresponding evidence support, and lack of scientific and rigorous. The guidelines issued by NCCN and NICE(26,29),1/1/0001 12:00:00 AM systematically evaluated the relevant research and conclusions, and recommended opinions and suggestions. However, these two guidelines did not point out the steps and methods of systematic retrieval of evidence, as well as the criteria for accepting and excluding the research. In addition to the guidelines issued by INS(30), the rest of the guidelines do not specify the steps and methods for updating the guidelines. However, the guidelines indicated the timing of the update(30), and the extent to which the evidence level of the new guideline should be higher than that of the previous edition of the guideline, and indicated that the quality of the evidence in the guideline is constantly improving. Due to the differences in medical and health systems at home and abroad and the non-standard evaluation of guidelines, the study found that most of the guidelines published abroad put forward statements of interest in the study(15,26,27,29,30,32). However, the guideline issued by the China Branch of the International Union of Angiology failed to state the statement of interest of the study. The interests of the panel members with other bodies are also not recorded and the fairness and independence of the study are not guaranteed. Therefore, we should pay full attention to the rigor and independence of the guideline, pursue evidence according to the concept of evidence-based medicine, and avoid the influence of external factors and the biases of research caused by personal preferences, to ensure the rigor and independence of research.

participants

In the participant section, only two guidelines issued by the Chinese Medical Association and INS(27,30), the names and work units of all the experts involved in the formulation of the guidelines are listed, while the rest of the guidelines refer only to interdisciplinary teams or association support and without specify the relevant information of the researchers. In addition to the release guidelines of the Chinese

For peer review only - http://bmjopen.bmj.com/site/about/guidelines.xhtml

Medical Association, NCCN, and ITAC-CME(23,25,30), the rest of the team did not explain how to collect comments and suggestions from users considering the relevant information of researchers, such as work experience, units, and professional titles, can reduce the bias caused by personal factors of researchers, which is conducive to the formulation of guidelines and reflects the universality and authority of the formulation of guidelines.

application

In the field of application, relevant studies have shown that the use of clinical guidelines is generally low. Most of the guidelines included in this study do not fully take into account the difficulties and obstacles that may be encountered in the follow-up application, nor are they accompanied by relevant systems and documents, and there is no training for clinical staff in the use of the guidelines, which is not conducive to the learning and use of the guidelines. Of the included guidelines, only this issued by NICE is presented(30). The guidelines are applied to specific clinical methods and measures, but the rest of the guidelines do not address the forward and reverse factors in the application of the guidelines and how to make use of potential resources and fail to transform the theory into practice well. Therefore, it is necessary to carry out pre-experiments in the process of guideline formulation in the future, check the feasibility of guideline recommendations in advance, collect the problems in the application of guidelines in advance, make corresponding preparations, and promote the transformation of guidelines to clinical practice.

analysis of the recommendations in the guidelines

risk screening

Timely assessment of risk factors for CRT formation in patients is beneficial to clinical prevention of CRT formation, in addition to the guidelines issued by SEOM and ITAC-AEM(15,27), the rest of the guidelines indicate that CRT risk factors need to be assessed before CVC intubation. The guidelines also clearly point out the high-risk factors of CRT, mainly divided into patient-, catheter-, and disease/treatment-related factors. The patient-related factors were age, sex, family history or personal

For peer review only - http://bmjopen.bmj.com/site/about/guidelines.xhtml

history of venous thrombosis, hypercoagulable state of blood, and abnormal coagulation gene. Catheter-related factors were catheter material, placement technique, location and type of catheter, and puncture times. Disease/treatment-related factors were antiangiogenic therapy, hormone therapy, parenteral nutrition and radiotherapy, tumor, and trauma, and complicated surgery. However, at present, there is no recognized scale dedicated to CRT risk assessment. Most of the guidelines recommend the use of the VTE risk factor assessment scale, and the two guidelines recommend the use of the Khorana scale and Wells scale(22,23,30). The guideline issued by NCCN recommends that patients with medical diseases can use the Padua scale(29). At the same time, it is suggested that myeloma patients should be treated with targeted anticoagulant therapy after scoring with IMPED and SAVED scales. CRT risk assessment should be carried out at admission, before and after catheterization and replacement, and when symptoms and signs occur. Timely evaluation of CRT-related symptoms of patients, regular examination of related physiological and biochemical indicators, according to the actual situation flexible adjustment of anticoagulant program, and the occurrence of special circumstances timely report.

Prevention strategies

Through the summary and analysis of the nine guidelines, it is found that the preventive measures are mainly divided into the following five parts: classified prevention, physical prevention, drug prevention, common CRT prevention measures, and multi-disciplinary prevention. Classified prevention emphasizes individualized prevention according to the patients' factors and the clinical manifestations of CRT. Physical prevention is mainly to help patients exercise actively or passively, promote their blood circulation, to reduce the formation of thrombosis. To urge patients to get out of bed as soon as possible, put tube side limbs to carry out functional exercises such as repeated clenching or loosening, promote blood circulation, timely replenish water, and prevent dehydration and blood coagulation. However, most guidelines indicate that patients at risk of post-thrombosis syndrome do not recommend routine use of elastic socks for prevention, as they may aggravate skin breakage and injury. Drug prevention mainly uses low-dose unfractionated heparin, low molecular weight heparin, and

For peer review only - http://bmjopen.bmj.com/site/about/guidelines.xhtml

vitamin K antagonists, while some guidelines recommend the use of a new direct oral anticoagulant DOAC. The risk of bleeding should be taken into account when anticoagulants are used, and the risks and benefits of the drug should be assessed and used with the informed consent of the patient. The common prevention of CRT is mainly the treatment of a catheter, including the choice of catheter diameter and lumen, the way of catheterization, the location of catheterization, the time of catheterization, and subsequent anticoagulant therapy, as shown in tables S4 and S5. Multi-disciplinary prevention refers to the construction of a multi-disciplinary team composed of vascular surgery, oncology, evidence-based medicine centers, medical laboratory technicians, and nurses to carry out CRT prevention, to avoid the limitations of clinical practice.

knowledge training

The guideline issued by INS suggests that nurses should constantly update their clinical knowledge and skills[39], and based on the concept of evidence-based transformation of the latest research into high-quality evidence, to promote its transformation to clinical practice. At present, the update of clinical nursing is slow, and most of the medical staff are still based on traditional nursing methods, so it is difficult to introduce new research results into clinical practice. Therefore, it is very necessary to carry out systematic scientific research education and organize reading exchange meetings and knowledge training for nurses. The guidelines issued by ASCO(26) indicate that, although CRT is closely related to cancer, patients' awareness of the risks and warning signals of CRT is still very low, emphasizing the need to increase patient education and awareness(28,37). At the same time, a study suggests that patients may not report new symptoms unless asked directly, as they usually consider symptoms to be a manifestation of cancer or a side effect of treatment. Therefore, health education for patients is helpful to ensure effective communication between medical staff and patients and is conducive to patients' understanding of the disease(38).

limitations of the guidelines.

Although one of the nine guidelines included in this study is a specific guideline

For peer review only - http://bmjopen.bmj.com/site/about/guidelines.xhtml

on CRT prevention, it does not systematically retrieve relevant research and evidence, and only recommends and compiles guidelines based on the experience of clinical experts, which lack scientific. Most of the other guidelines are suitable for cancer patients, and the main prevention strategies related to CRT are drug prevention and CVC catheter prevention, but their contents are fewer and their views are scattered, the subjects of the guidelines are limited, and lack of systematic integrated evaluation. Therefore, the formulation of evidence-based CRT prevention-related clinical practice guidelines is helpful to guideline clinical staff to standardize the operation and ensure the treatment quality of patients. At the same time, after analyzing the content of the guideline, it is found that there is no tool dedicated to CRT risk assessment. At present, the guidelines recommend the use of a mature VTE risk assessment scale, but this kind of scale does not involve the evaluation of the CVC catheter, which is not targeted, which may lead to a bias of measurement. Therefore, the development of a risk assessment scale for CRT is conducive to the accurate selection of appropriate preventive measures and anticoagulant treatment methods and reduces the injury caused to patients after the occurrence of CRT.

In summary, the quality of the nine clinical guidelines included in this study is moderately high, but there are some differences in the guidelines in different regions and countries. The guidelines need to be further improved in the following aspects: (1) there is no risk assessment scale dedicated to CRT, and a targeted evaluation form should be developed based on evidence-based evidence, which is conducive to the accurate selection of appropriate preventive measures and anticoagulation treatments; (2) clinical practice guidelines and risk assessment scales related to CRT prevention based on evidence-based concepts should be formulated to enable patients to participate in the study based on the clinical status quo to increase the practicability of the guidelines.

Conclusion

For peer review only - http://bmjopen.bmj.com/site/about/guidelines.xhtml

The overall quality of the nine guidelines included is good, and the corresponding advice and suggestions about CRT prevention are provided, but the guidelines still need to be improved in terms of rigor and independence of writing, participants, and applications. At present, there is still a lack of high-quality clinical practice guidelines for CRT prevention, and there is also a lack of dedicated CRT risk assessment tools. Therefore, there is an urgent need to develop evidence-based CRT prevention-related clinical practice guidelines and risk assessment tools based on the clinical situation to enable patients to participate in the management of CRT and, thereafter to to better manage the CRT.

Contributors

GW and XH conceived and supervised the project. JZ, YW, and SZ summed up the literature and drafted the manuscript. WY, FB, and AW drew the figures and tables. GW and JZ revised the manuscript. All authors contributed, edited, and approved the final manuscript.

Funding

This work was supported by the Sichuan Science and Technology Program (Grant 2022NSFSC1290), and the West China Nursing Discipline Development Special Fund Project, Sichuan University (Grant HXHL21011).

Competing interests

The authors declare that they have no conflicts of interest.

Patient and public involvement

Patients and/or the public were not involved in the design, conduct, reporting, or dissemination plans of this research.

For peer review only - http://bmjopen.bmj.com/site/about/guidelines.xhtml

Patient consent for publication

Not required

Provenance and peer review

Not commissioned; externally peer-reviewed

Ethics approval

Not involved.

Data sharing statement

The manuscript was a systematic review of published studies; the full references of these studies have been provided in the reference.

Supplemental material

This content has been supplied by the author(s). It has not been vetted by BMJ Publishing Group Limited (BMJ) and may not have been peer-reviewed. Any opinions or recommendations discussed are solely those of the author(s) and are not endorsed by BMJ. BMJ disclaims all liability and responsibility arising from any reliance placed on the content. Where the content includes any translated material, BMJ does not warrant the accuracy and reliability of the translations (including but not limited to local regulations, clinical guidelines, terminology, drug names, and drug dosages), and is not responsible for any error and/or omissions arising from translation and adaptation or otherwise.

Open access

This is an open-access article distributed in accordance with the Creative Commons Attribution Non Commercial (CC BY-NC 4.0) license, which permits others to distribute, remix, adapt, build upon this work non-commercially, and license their

For peer review only - http://bmjopen.bmj.com/site/about/guidelines.xhtml

derivative works on different terms, provided the original work is properly cited, appropriate credit is given, any changes made indicated, and the use is non-commercial. See: http://creativecommons.org/licenses/by-nc/4.

For peer review only

References

1. Smith RN, Nolan JP. Central venous catheters. BMJ. 2013 Nov 11;347:f6570.
2. Expert Group on Safety Management of Central Venous Access Device. Zhonghua Wai Ke Za Zhi. 2020 Apr 1;58(4):261–72.
3. Matar CF, Kahale LA, Hakoum MB, Tsolakian IG, Etxeandia-Ikobaltzeta I, Yosuico VE, et al. Anticoagulation for perioperative thromboprophylaxis in people with cancer. Cochrane Database Syst Rev. 2018 Jul 11;7(7):CD009447.
4. Hao N, Xie X, Zhou Z, Li J, Kang L, Wu H, et al. Nomogram predicted risk of peripherally inserted central catheter related thrombosis. Sci Rep. 2017 Jul 24;7(1):6344.
5. Liu Y, Gao Y, Wei L, Chen W, Ma X, Song L. Peripherally inserted central catheter thrombosis incidence and risk factors in cancer patients: a double-center prospective investigation. Ther Clin Risk Manag. 2015;11:153–60.
6. Meyer BM. Developing an alternative workflow model for peripherally inserted central catheter placement. J Infus Nurs. 2012;35(1):34–42.
7. Falanga A, Ay C, Di Nisio M, Gerotziafas G, Jara-Palomares L, Langer F, et al. Venous thromboembolism in cancer patients: ESMO Clinical Practice Guideline. Ann Oncol. 2023 May;34(5):452–67.
8. Takemoto CM, Sohi S, Desai K, Bharaj R, Khanna A, McFarland S, et al. Hospital-associated venous thromboembolism in children: incidence and clinical characteristics. J Pediatr. 2014 Feb;164(2):332–8.
9. Chen Y, Chen H, Yang J, Jin W, Fu D, Liu M, et al. Patterns and risk factors of peripherally inserted central venous catheter-related symptomatic thrombosis events in patients with malignant tumors receiving chemotherapy. J Vasc Surg Venous Lymphat Disord. 2020 Nov;8(6):919–29.
10. Chopra V, Anand S, Krein SL, Chenoweth C, Saint S. Bloodstream infection, venous thrombosis, and peripherally inserted central catheters: reappraising the evidence. Am J Med. 2012 Aug;125(8):733–41.
11. Marnejon T, Angelo D, Abu Abdou A, Gemmel D. Risk factors for upper extremity venous thrombosis associated with peripherally inserted central venous catheters. J Vasc Access. 2012;13(2):231–8.
12. Chopra V, Anand S, Hickner A, Buist M, Rogers MA, Saint S, et al. Risk of venous thromboembolism associated with peripherally inserted central catheters: a systematic review and meta-analysis. Lancet. 2013 Jul 27;382(9889):311–25.
13. Lowell JA, Bothe A. Central venous catheter related thrombosis. Surg Oncol Clin N Am. 1995 Jul;4(3):479–92.
14. Tran BH, Nguyen TJ, Hwang BH, Vidar EN, Davis GB, Chan LS, et al. Risk factors associated with venous thromboembolism in 49,028 mastectomy patients. Breast. 2013 Aug;22(4):444–8.
15. Ferro JM, Bousser MG, Canhão P, Coutinho JM, Crassard I, Dentali F, et al. European Stroke Organization guideline for the diagnosis and treatment of cerebral

For peer review only - http://bmjopen.bmj.com/site/about/guidelines.xhtml

venous thrombosis - endorsed by the European Academy of Neurology. Eur J Neurol. 2017 Oct;24(10):1203–13.
16. Jones D, Wismayer K, Bozas G, Palmer J, Elliott M, Maraveyas A. The risk of venous thromboembolism associated with peripherally inserted central catheters in ambulant cancer patients. Thromb J. 2017;15:25.
17. Chen H, Tao L, Zhang X, Jing W, Su X, Chen H, et al. The effect of systemic and local risk factors on triggering peripherally inserted central catheter-related thrombosis in cancer patients: A prospective cohort study based on ultrasound examination and structural equation modeling. Int J Nurs Stud. 2021 Sep;121:104003.
18. Duffett L. Deep Venous Thrombosis. Ann Intern Med. 2022 Sep;175(9):ITC129–44.
19. Ahn DH, Illum HB, Wang DH, Sharma A, Dowell JE. Upper extremity venous thrombosis in patients with cancer with peripherally inserted central venous catheters: a retrospective analysis of risk factors. J Oncol Pract. 2013 Jan;9(1):e8-12.
20. Wang G, Li Y, Wu C, Guo L, Hao L, Liao H, et al. The clinical features and related factors of PICC-related upper extremity asymptomatic venous thrombosis in cancer patients: A prospective study. Medicine (Baltimore). 2020 Mar;99(12):e19409.
21. Bonizzoli M, Batacchi S, Cianchi G, Zagli G, Lapi F, Tucci V, et al. Peripherally inserted central venous catheters and central venous catheters related thrombosis in post-critical patients. Intensive Care Med. 2011 Feb;37(2):284–9.
22. Chopra V, Kaatz S, Grant P, Swaminathan L, Boldenow T, Conlon A, et al. Risk of Venous Thromboembolism Following Peripherally Inserted Central Catheter Exchange: An Analysis of 23,000 Hospitalized Patients. Am J Med. 2018 Jun;131(6):651–60.
23. Institute of Medicine (US) Committee to Advise the Public Health Service on Clinical Practice Guidelines. Clinical Practice Guidelines: Directions for a New Program. Field MJ, Lohr KN, editors. Washington (DC): National Academies Press (US); 1990. Available from: http://www.ncbi.nlm.nih.gov/books/NBK235751/
24. Brouwers MC, Kho ME, Browman GP, Burgers JS, Cluzeau F, Feder G, et al. AGREE II: Advancing guideline development, reporting, and evaluation in health care. Preventive Medicine. 2010 Nov 1;51(5):421–4.
25. Development and Validation of a Tool to Assess the Quality of Clinical Practice Guideline Recommendations. JAMA Netw Open. 2020;3(5):e205535. Published 2020 May 1
26. Key NS, Khorana AA, Kuderer NM, Bohlke K, Lee AYY, Arcelus JI, et al. Venous Thromboembolism Prophylaxis and Treatment in Patients With Cancer: ASCO Clinical Practice Guideline Update. J Clin Oncol. 2020 Feb 10;38(5):496–520.
27. Farge D, Frere C, Connors JM, Ay C, Khorana AA, Munoz A, et al. 2019 international clinical practice guidelines for the treatment and prophylaxis of venous thromboembolism in patients with cancer. Lancet Oncol. 2019 Oct;20(10):e566–81.
28. Sousou T, Khorana AA. Cancer patients and awareness of venous thromboembolism. Cancer Invest. 2010 Jan;28(1):44–5.
29. Streiff MB, Holmstrom B, Angelini D, Ashrani A, Elshoury A, Fanikos J, et al. Cancer-Associated Venous Thromboembolic Disease, Version 2.2021, NCCN

For peer review only

For peer review only - http://bmjopen.bmj.com/site/about/guidelines.xhtml

Clinical Practice Guidelines in Oncology. J Natl Compr Canc Netw. 2021 Oct 15;19(10):1181–201.
30. Hinck SM. Implementing the Infusion Therapy Standards of Practice. Home Healthc Now. 2021 Oct 1;39(5):295.
31. Venous thromboembolism in over 16s: reducing the risk of hospital-acquired deep vein thrombosis or pulmonary embolism London: National Institute for Health and Care Excellence (NICE); 2019. (National Institute for Health and Care Excellence: Guidelines).
32. Witt DM, Nieuwlaat R, Clark NP, Ansell J, Holbrook A, Skov J, et al. American Society of Hematology 2018 guidelines for management of venous thromboembolism: optimal management of anticoagulation therapy. Blood Adv. 2018 Nov 27;2(22):3257–91.
33. Chinese Expert Consensus on the Prevention and Treatment of Infusion Catheter-related Venous Thrombosis (2020 Edition) [J]. Chinese Journal of Practical Surgery,2020,40(4):377-383.
34. Guidelines for the prevention and treatment of thrombotic diseases in China - Chinese Medical Journal. National Medical Journal of China. 2018 Sep 25;98(36):2861–88.
35. SEOM clinical guideline of venous thromboembolism (VTE) and cancer (2019) - PubMed.
36. Infusion Therapy Standards of Practice, 8th Edition : Journal of Infusion Nursing.
37. Aggarwal A, Fullam L, Brownstein AP, Maynard GA, Ansell J, Varga EA, et al. Deep vein thrombosis (DVT) and pulmonary embolism (PE): awareness and prophylaxis practices reported by patients with cancer. Cancer Invest. 2015;33(9):405–10.
38. Gilligan T, Coyle N, Frankel RM, Berry DL, Bohlke K, Epstein RM, et al. Patient-Clinician Communication: American Society of Clinical Oncology Consensus Guideline. J Clin Oncol. 2017 Nov 1;35(31):3618–32.

For peer review only

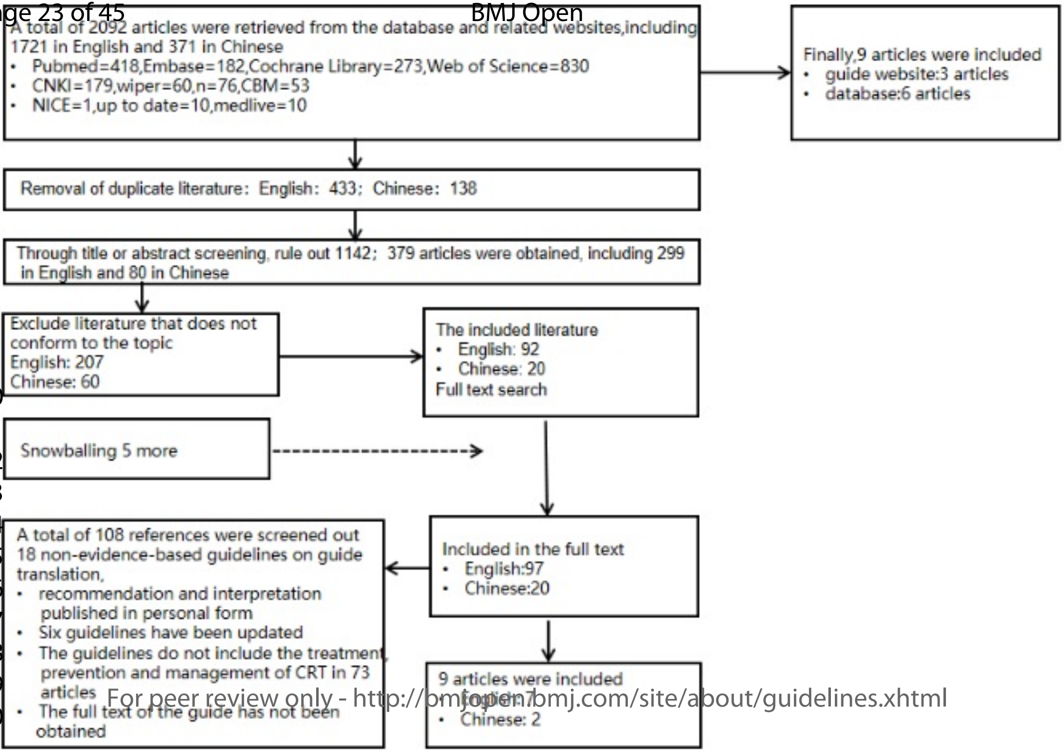

For peer review only - http://bmjopen.bmj.com/site/about/guidelines.xhtml

Table S1. Search strategy in Pubmed

No	Search type
#1	Catheter-related thrombosis [Title/Abstract]
# 2	Catheter-related thromboembolism [Title/Abstract]
# 3	Peripherally inserted central catheter,-related thrombosis [Title/Abstract]
# 4	Venous thromboembolism [Title/Abstract]
# 5	Venous thrombosis [Title/Abstract]
# 6	Vein thrombosis [Title/Abstract]
# 7	Catheter-related thrombosis [MeSH Terms]
# 8	Catheter-related thromboembolism [MeSH Terms]
# 9	Peripherally inserted central catheter-related thrombosis [MeSH Terms]
# 10	Venous thromboembolism [MeSH Terms]
# 11	Venous thrombosis [MeSH Terms]
# 12	Vein thrombosis [MeSH Terms]
# 13	# 1 OR # 2 OR # 3 OR # 4 OR # 5 OR # 6 OR # 7 OR # 8 OR # 9 OR # 10 OR # 11 OR # 12
# 14	((clinical practice guidelineline [Title/Abstract]) OR (guidance [title / Abstract]) OR (Evidence-Based Practice [Title/Abstract]) Filters: from 2017-2022
# 15	# 13 AND # 14

For peer review only - http://bmjopen.bmj.com/site/about/guidelines.xhtml

Table S2. Includes the basic characteristics table of the guideline

Number	Name	Year	Publishing agency	Subject	language	Whether or not evidence-based guidelines	Number of references
1	Chinese Expert Consensus on the Prevention and Treatment of Infusion Catheter-related Venous Thrombosis (2020 Edition)	2020	Vascular Alliance China Chapter	Catheter-related thrombosis prevention	Chinese	Not	62
2	Venous Thromboembolism Prophylaxis and Treatment in Patients With Cancer: ASCO Clinical Practice Guideline Update	2019	American Society of Clinical Oncology	Prevention of cancer-related blood clots	English	Yes	155
3	Guidelines for the prevention and treatment of thrombotic diseases in China	2018	Chinese Medical Association	Diagnosis and prevention of thrombosis	Chinese	Not	41
4	SEOM clinical guideline of venous thromboembolism (VTE) and cancer	2019	Spanish Society of Medical Oncology	Prevention and treatment of cancer-related thrombosis	English	Yes	88
5	Cancer Associated Venous Thromboembolic Disease	2021	National Comprehensive Cancer Network	Strategies for the assessment, prevention, and management of cancer-related thrombosis	English	Yes	222

6	American Society of Hematology 2020 guidelines for management of venous thromboembolism: optimal management of anticoagulation therapy treatment and prophylaxis of venous thromboembolism in patients with cancer	2020	American Society of Hematology	Drug prophylaxis strategies for venous thrombosis	English	Yes	360
7	2019 international clinical practice guidelines for thetreatment and prophylaxis of venous thromboembolism in patients with cancer treatment and prophylaxis of venous thromboembolism in patients with cancer	2019	International Thrombosis and Cancer Initiative	Prevention strategies for cancer-related thrombosis	English	Yes	94
8	Venous thromboembolism in over 16s: reducing the risk of hospital-acquired deep vein thrombosis or pulmonary embolism	2018	National Institute for Health and Care Excellence, UK	Strategies for the prevention and treatment of blood clots	English	Not	330
9	Infusion Therapy Standards of Practice	2021	Infusion Nurses Association	Methods and strategies of clinical infusion therapy	English	Not	471

For peer review only - http://bmjopen.bmj.com/site/about/guidelines.xhtml

Note: American Society of Clinical Oncology (ASCO); Spanish Society of Medical Oncology (SEMO); National Comprehensive Cancer Network (NCCN); American Society Hematological(ASO); International Initiative on Thrombosis and Cancer(ITAC-CME); National Institute for Health and Clinical Excellence(NICE); Infusion Nursing(INS).

For peer review only

For peer review only - http://bmjopen.bmj.com/site/about/guidelines.xhtml

Table S3 Specific standardized scores for each AGREE II domain

number	Standardized score by domain (%)						The number of standardized scores ≧ 60%.	recommend level
	range and objective	Participants	Rigour	Clarity	Application	independence		
1	62.96	31.48	31.94	64.81	31.94	44.44	2	B
2	75.93	72.22	63.89	68.52	66.67	75.00	6	A
3	74.07	81.48	77.08	55.56	72.22	69.44	5	B
4	38.89	33.33	30.56	61.11	41.67	61.11	2	B
5	70.37	68.52	66.67	62.96	47.22	61.11	5	B
6	79.63	83.33	84.03	77.78	80.56	72.22	6	A
7	64.81	62.96	64.58	61.11	66.67	52.78	5	B
8	72.22	77.78	72.92	72.22	69.44	72.22	6	A
9	61.11	35.19	65.28	59.26	68.06	63.89	4	B
Mean Score	66.67	60.70	61.88	64.81	60.49	63.58	-	-

For peer review only

For peer review only - http://bmjopen.bmj.com/site/about/guidelines.xhtml

Table S4 Specific standardized scores of AGREE REX in each field

number	AGREE REX Score by Sector (%)			Overall rating
	Clinical applicability	Values and preferences	Implementability	
1	42.59	41.67	50.00	43.83
2	61.11	61.11	66.67	62.35
3	74.07	65.28	77.78	70.99
4	37.04	41.67	61.11	44.44
5	50.00	61.11	72.22	59.88
6	83.33	73.61	77.78	77.78
7	59.26	50.00	63.89	56.17
8	66.67	66.67	77.78	69.14
9	48.15	59.72	52.78	54.32

For peer review only

Table S5. Summary of recommendations included in the guidelines

Items		Source guideline	Recommendation
Risk screening	Thrombosis assessment tool	1、2、3、5、7、8、9	1. The main risk factors assessed included catheterization factors (catheter materials, placement techniques, location and type of catheter, presence of other catheters or catheters, etc.); potential co-disease factors (antiangiogenic therapy, hormone therapy, parenteral nutrition and radiotherapy, tumor and trauma and complex surgery) Patient factors (age, gender, family history or medical history of venous thrombosis, hypercoagulation status and abnormal coagulation gene).<br>2. At present, there is no scale dedicated to CRT risk assessment, and it is recommended to use mature VTE risk assessment scale in clinical practice. Modified Khorana score and Wells scale should be used in CRT risk assessment. Padua scale is recommended for medical patients and IMPED and SAVED scales are recommended for patients with multiple myeloma.
	Timing of thrombo	1、2、3、5、8、9	3. Admission / pre-catheterization / maintenance catheter / symptoms and signs / catheter extraction / discharge / anticoagulant therapy.

For peer review only - http://bmjopen.bmj.com/site/about/guidelines.xhtml

	sis evaluation		
Preventions	Identify as soon as possible	1、3、9	4. Pay close attention to CRT related symptoms and signs such as pain, swelling and skin pruritus on the side of catheterization, and monitor blood routine and coagulation function in time.
	Stay alert.	2、4	5. Correct the wrong operation in time through quality control, conduct regular staff meetings to summarize and feedback on the recent work, and formulate staff briefing warnings to remind other personnel.
	Timely report	1、2、4、8	6. Pay attention to high-risk patients and timely report adverse reactions to prevent them.
	Classified prevention	1、2、3、5、8	7. To evaluate the risk factors and existing symptoms and signs of CRT in patients, and target to prevent and manage them according to different factors and clinical manifestations.<br>8. According to the risk benefit model, individualized drug prevention and physical prevention.
	Multi-disciplinary preventi	1、2、4、5、6、9	9. To construct a venous access management team composed of doctors, nurses, pharmacists, medical technologists, evidence-based medicine experts and managers to improve the scientific and comprehensive of CRT prevention and

For peer review only

	on		treatment.
	Physical preventi on	1、2、5、8、9	10.Patients who stay in bed for a long time or have limited activity can massage their lower extremities with intermittent inflatable pressure pump to prevent thrombosis for 1 to 2 weeks. The skin condition of the patients should be evaluated before and after use.<br>11.To carry out daily activities as soon as possible, CVC catheter side should carry out early functional exercise, such as repeatedly clenching , loose, chest breathing and abdominal breathing, etc.<br>12.Replenish the patient with water in time to relieve dehydration.<br>13.For patients at risk of post-thrombosis syndrome, elastic socks are not recommended.
Preventi ons	Drug preventi on	2、4、5、6、8	14.After evaluating the risk of bleeding and thrombosis, unfractionated heparin, low molecular heparin, vitamin K antagonist or direct oral anticoagulant drug can be used for prevention. After taking the drug, the laboratory indexes of the patients should be monitored, INR should be monitored in patients with Warfarin, and the arm circumference of the catheterization side should be measured regularly to ask if the patient has bleeding related manifestations.

For peer review only

Common catheter-related thrombosis prevention	1、2、3、4、5、6、7、8、9	15. When CVC intubation, the catheter with small diameter, less lumen and less trauma should be selected. If conditions permit, the ratio of the outer diameter of the catheter to the diameter of the catheterized vein should be less than 45%.<br>16. Ultrasonic guided intubation should be used in CVC catheterization, the elbow and other frequently moving positions should be avoided when catheterization, upper elbow with low activity, large diameter veins, fast blood flow rate and more blood flow should be selected. The catheter tip should be the lower third of the superior vena cava or the intersection of chambers<br>17. At present, CVC is not removed except for complications such as any longer indwelling CVC, blockage or dysfunction of CVC catheter, not within normal range, complicated with CRT-related blood flow infection, and so on.<br>18. It is recommended that CVC should be treated with anticoagulant therapy for 1 or 2 weeks before extubation, and then extubation should be anticoagulation at least 3 months after extubation. If CVC functions are normal and decide to continue to use it, even if the anticoagulant period has reached 3 months, it is necessary to continue anticoagulation until the CVC catheter is completely removed.

For peer review only - http://bmjopen.bmj.com/site/about/guidelines.xhtml

Knowledge training	medical staff	2、9	20. Learn CRT diagnosis, prevention related knowledge, master the correct treatment process, participate in bedside rounds and medical record discussions, participate in other healthcare organizations and expert academic exchanges
	Patient	2、3、9	21. Inform patients of disease-related knowledge, such as physical preventive measures, related symptoms and signs, side effects of drugs, emergency management, etc.
	Multi-disciplinary participation	2、3、5、7	22. Multi-disciplinary sharing, active organization of book exchange meetings, regular academic sharing.

Table S6. Summary of views on preventive measures included in the guidelines

Number	Advice and recommendation	Do not recommend comments
1	In order to reduce the injury caused by CVC, the catheter with small diameter and small number of lumens should be selected as much as possible; ultrasonic guidance should be used to reduce the number of catheterization in CVC catheterization;	Prophylactic use of anticoagulant or thrombolysis drugs is not recommended.

For peer review only - http://bmjopen.bmj.com/site/about/guidelines.xhtml

	the elbow joint that often moves should be avoided in CVC catheterization and the upper elbow should be selected; The middle and lower 1/3 of superior vena cava and the intersection of chambers and chambers is the best intubation choice at the tip of CVC; early active after CVC catheterization. The indexes of blood routine and coagulation function and related symptoms and signs were monitored in time after the use of anticoagulant drugs.	Chest enhancement CT and three-dimensional lung imaging are not recommended to screen CRT patients for pulmonary embolism.
2	The prevention of CRT in cancer patients can be based on the combination of drug prevention and mechanical prevention; drug prevention can use heparin (low molecular weight heparin, unfractionated heparin) drugs; CRT high risk patients should be evaluated according to the actual situation after they can decide the time to start anticoagulation; CRT high risk patients (major surgery patients and cancer patients) should be given health education.	Mechanical prophylaxis is not recommended as a single measure of VTE prevention; anticoagulant is not recommended to improve the survival rate of cancer patients without VTE symptoms.
3	CVC function is normal, the catheter can be retained; all patients should be treated with anticoagulant treatment after removing CVC for at least 3 months; if the anticoagulant time	For patients with deep venous thrombosis at risk of

For peer review only - http://bmjopen.bmj.com/site/about/guidelines.xhtml

	is more than 3 months after CVC catheterization, anticoagulation should continue until the catheter is removed.	post-thrombotic syndrom , routine use of elastic socks is not recommended.
4	Risk factors for CRT formation include catheter factors (catheter material, placement techniques, location and type of catheter) and potential co-disease factors (antiangiogenic therapy, hormone therapy, parenteral nutrition and radiotherapy), as well as patient factors (age, sex and thrombosis-related diseases) The first choice of prevention after CVC intubation is to use low molecular heparin anticoagulant therapy for 3-6 months, if CVC has not been removed for more than 3 months, then low molecular heparin is used for anticoagulation indefinitely; direct oral anticoagulant DOAC is also recommended; if anticoagulant therapy is ineffective or anticoagulant failure, CVC should be removed after 5 or 7 days of anticoagulation.	Except for CVC related infection, unsuitable anticoagulant, poor anticoagulant effect and decision to remove CVC, CVC transfer is not recommended..
5	If the CVC function is normal, you can continue to use it. When CVC is retained, it is recommended that anticoagulant therapy be carried out for CRT formation; if CVC is no longer needed, anticoagulant therapy should be performed 5 or 7 days before CVC removal; if CVC related infection, CRT or	Prophylactic anticoagulant therapy for patients with CVC intubation is not recommended.

For peer review only - http://bmjopen.bmj.com/site/about/guidelines.xhtml

	worsening symptoms, CVC should be removed when there is anticoagulant contraindication, and the risk of thrombosis should be taken into account, and the anticoagulant treatment should be decided according to the specific situation. In order to prevent CRT, cancer patients should be treated with anticoagulant therapy for at least 3 months, or anticoagulant therapy should be performed throughout the catheterization period; medical personnel should inform patients of the risks and benefits of anticoagulant, and determine the duration of treatment after being known to the patient.	
6	The use of anticoagulant drugs alone in the treatment of symptomatic CRT in children can minimize complications and do not need to be pulled out of the catheter; for non-functional or no longer used CVC, it is recommended that anticoagulant therapy remove CVC a few days later.	Multiple CVC intubation is not recommended; routine use of elastic socks is not recommended.
7	The right jugular vein is the first choice for CVC catheterization, in which the intersection of the right atrium and the superior vena cava is the best position for the distal tip of the catheter; for patients with PICC catheterization, the implantation port should be used; for symptomatic CRT, anticoagulant therapy should be performed for at least 3	Undifferentiated anticoagulant therapy is not recommended for prevention of CRT formation.

For peer review only - http://bmjopen.bmj.com/site/about/guidelines.xhtml

	months as long as CVC is in situ; there is no guideline to compare the anticoagulant effects of low molecular weight heparin, DOAC and vitamin K antagonists If the CVC catheter is unblocked the position of the catheter is normal, no infection occurs, and other symptoms disappear during anticoagulant, CVC can remain in place.	
8	When considering whether to use drugs to prevent CRT, it is necessary to analyze the risks and benefits of anticoagulant; LMWH or DOAC can be selected for CRT drug prevention; patients should be educated about VTE risk factors and related symptoms and signs.	Routine thrombosis prevention is not recommended in all patients; thrombosis prevention time is not recommended for less than 12 weeks.
9	The risk of CRT should be assessed before CVC intubation; the appropriate intubation location and catheter type should be selected before catheterization; the selected veins should be observed and measured by ultrasound in advance before PICC intubation, and the catheter with the ratio of catheter diameter to vein diameter less than 45% should be preferred; the tip of CVC should be located at 1/3 of superior vena cava or the junction of vena cava and atrial vein; and the circumference of upper arm should be measured before insertion of PICC to	It is not recommended that CVC intubation be located in the anterior cubital fossa and prophylactic anticoagulant therapy is not recommended.

For peer review only

For peer review only - http://bmjopen.bmj.com/site/about/guidelines.xhtml

assess the presence of edema and deep venous thrombosis. When CVC is located correctly, functionally normal and has no sign of infection, CVC does not need to be removed even if deep venous thrombosis exists.

For peer review only

For peer review only - http://bmjopen.bmj.com/site/about/guidelines.xhtml

Revised Standards for Quality Improvement Reporting Excellence (SQUIRE 2.0) September 15, 2015

Text Section and Item Name	Section or Item Description
Notes to authors	. The SQUIRE guidelines provide a framework for reporting new knowledge about how to improve healthcare<br>. The SQUIRE guidelines are intended for reports that describe systemlevel work to improve the quality, safety, and value of healthcare, and used methods to establish that observed outcomes were due to the intervention(s).<br>. A range of approaches exists for improving healthcare. SQUIRE may be adapted for reporting any of these.<br>. Authors should consider every SQUIRE item, but it may be inappropriate or unnecessary to include every SQUIRE element in a particular manuscript.<br>. The SQUIRE Glossary contains definitions of many of the key words in SQUIRE.<br>. The Explanation and Elaboration document provides specific examples of well-written SQUIRE items, and an in-depth explanation of each item.<br>. Please cite SQUIRE when it is used to write a manuscript.
**Title and Abstract**	
1. Title	Appraising the quality standard of clinical practice guidelines related to central venous catheter-related thrombosis prevention: a systematic review of reviews(Page 1)
2. Abstract	Background: Central venous catheter (CVC)operation is convenient and relatively safe, and it is widely used in clinical practice. Catheter-associated venous thrombosis is one of the common complications after CVC catheterization. Several clinical practice guidelines(CPGs) for Catheter-related thrombosis (CRT) prevention have been published, but their quality is unclear.<br>Objective: To evaluate the quality and analyze the content of clinical practice guidelines regarding central venous catheter-related thrombosis to provide evidence for formulating an evidence-based practice protocol and a risk assessment scale to prevent it.<br>Methods: Clinical practice guidelines regarding central venous catheter-related thrombosis prevention were searched in guidelines websites and databases such as the National Institute for Health and Care Excellence, Up to Date, Pubmed, Web of Science, CNKI, and others. The included guidelines were evaluated using AGREE II.<br>Results: A total of nine guidelines were included, and the quality grade results showed that three were at A-level and six were at B-level. The included

For peer review only - http://bmjopen.bmj.com/site/about/guidelines.xhtml

	guidelines mainly recommended the prevention measure of central venous catheter-related thrombosis from three aspects: risk screening, prevention strategies, and knowledge training, with a total of 22 suggestions being recommended.<br>Conclusion: The overall quality of the guidelines is high, but there are few preventive measures for central venous catheter-related thrombosis involved in the guidelines. All preventive measures have not been systematically integrated and evaluated, and no risk assessment scale dedicated to this field has been recommended. Therefore it is urgent to develop an evidence-based practice protocol and a risk assessment scale to prevent it.(Page 2)
**Introduction**	*Why did you start?*
**3. Problem Description**	Catheter-related venous thrombosis is a common complication of central venous catheters in chemotherapy, haemodialysis, nutritional support, and surgical support, and several clinical practice guidelines on CRT prevention have been published, but their quality is unclear. It makes sense to evaluate the quality and analyze the content of the guide. (Page 2)
**4. Available knowledge**	assessment and taking measurements early to prevent patients at high risk of CRT risk are essential to ensure the safety of patients. Clinical Practice guideline is formulated by clinical workers after systematically integrating and evaluating relevant research and clinical experience according to evidence-based concept to a certain common symptom, disease or problem, to provide guiding suggestions for clinical workers and patients, and scientifically promote the application of evidence in clinical practice. At present, several clinical practice guidelines about CRT prevention have been published, but their quality is unclear. Therefore, through the systematic retrieval, screening and reading of clinical practice guidelines related to CRT prevention, and the quality evaluation and content analysis of the guidelines, this review was expected to provide scientific basis for the development of evidence-based practice protocols and risk assessment scales for CRT. (Page 4)

**5. Rationale**	We searched guideline websites and databases for clinical practice guidelines on central venous catheter-associated thrombosis, and assessed the included guidelines using AGREEII and AGREE REX scale. (Page 6)
**6. Specific aims**	To evaluate the quality and analyze the content of clinical practice guidelines regarding central venous catheter-related thrombosis to provide evidence for formulating an evidence-based practice protocol and a risk assessment scale to prevent it. (Page 2)
**Methods**	*What did you do?*
**7. Context**	Central venous catheter (CVC)operation is convenient and relatively safe, and it is widely used in clinical practice. Catheter-associated venous thrombosis is one of the common complications after CVC catheterization. Several clinical practice guidelines(CPGs) for Catheter-related thrombosis (CRT) prevention have been published, but their quality is unclear. (Page 2)
**8. Intervention(s)**	a. Clinical practice guidelines regarding central venous catheter-related thrombosis prevention were searched in guidelines websites and databases such as National Institute for Health and Care Excellence, Up to date, Pubmed, Web of Science, CNKI, and others. The included guidelines were evaluated using AGREE II and AGREE REX. (Page 4-6).<br>b. Three researchers who systematically trained the use methods of the AGREE II scale and AGREE REX scale were selected to evaluate the guideline to ensure that the researchers had a full understanding and consistent understanding of the items and to reduce the biases of evaluation. If there is a dispute over an item, experts in the relevant field would be consulted to resolve it. (Page 6)
**9. Study of the Intervention(s)**	The AGREE II scale, a widely used tool for evaluating the quality of the guideline, was used to evaluate the included guidelines. It includes 6 domains, i.e. scope and purpose, rigorism, independence, clarity, application, and independence of writing, with 23 items.<br>the AGREE REX scale was used to evaluate the included guidelines, and three researchers who had been systematically trained in the use of the AGREE REX scale evaluated the guidelines respectively, and the scores of the three researchers in each area of the guidelines were standardized to calculate, and the guidelines were systematically evaluated and content analyzed and summarized based on the evidence-based concept. Referring to the recommendations of the AGREE REX manual, the guidelines define an overall score of >70% as high quality, an overall score of <30% as low quality, and others as moderate quality.(Page 6)
**10. Measures**	We searched both English and Chinese databases, including Pubmed, Embase, Cochrane Library, Web of Science, CNKI, Wanfang, VIP, and the Chinese Biomedical Literature Database from January 1st, 2017 to March 26th, 2022. In addition, the relevant websites of the guideline, including to date, National Institute for Health and Care Excellence (NICE), and Medline, were searched. The manual search of relevant studies and bibliography was also adopted.<br>Three researchers who had systematically trained the use of the AGREE II and AGREE REX scale were selected to conduct relevant research on the guidelines using the scale and evaluate their quality, including a total of 6 areas, including scope and purpose, study rigor, independence, clarity of discussion, application, and independence of writing, a total of 23 items, and SPSS22.0 was used to count the scores of two investigators in each field of the guidelines and calculate the relevant results.(Page 6-7)
**11. Analysis**	SPSS22.0 was used to count the scores of the two researchers in each field of the guideline, and the Intraclass Correlation Coefficient (ICC) of the data was calculated. According to the results, the consistency of the two researchers' evaluation of the guideline was tested. ICC < 0.4 indicated poor consistency, $0.40 \leq 0.59$ indicated that the consistency is general, $0.60 \leq 0.74$ indicated good consistency, and > 0.74 indicated that the consistency is very good. (Page 7)

For peer review only

**12. Ethical Considerations**	Competing interests : The authors declare that they have no conflicts of interest.<br>Ethical considerations : Not involved
**Results**	*What did you find?*
**13. Results**	a. A total of 2092 articles were retrieved, and 571 articles were removed by software and manual deletion. After read the title and abstract, 1142 articles were excluded. 379 articles were screened according to inclusion and exclusion criteria. 267 articles that did not meet the theme of the article were excluded, and 5 related guidelines were traced through the snowball strategy, and 117 articles were finally selected. Among them, 11 articles were not obtained, and a total of 106 complete articles were obtained. Full-text screened excluded 18 non-evidence-based guidelines issued in individual form, 6 guidelines that had been updated, and 73 guidelines that did not include CRT treatment, prevention, and management. Finally, 9 literatures were selected, including 2 Chinese literatures and 7 English literatures. (Page 7)<br>b. The nine guidelines were from the American Society of Clinical Oncology, the International Initiative on Thrombosis and Cancer, the Chinese Medical Association, the Spanish Medical Oncology Society, the Infusion Nursing Association, and other international authoritative medical associations. Only one of them is a guideline targeted for CRT risk assessment, prevention, and intervention18. Five guidelines were specifically for venous thrombosis prevention in cancer patients, and the content related to CRT was only part of it. Two of which are guidelines for venous thrombosis of ordinary patients. CRT patients are part of the content, and only one guideline is targeted for clinical infusion therapy. CRT is part of the treatment.(Page 8)<br>c. After scoring the nine guidelines with the AGREEII tool, it was found that the recommended A-level guidelines were three. The recommended B-level guidelines are six. The average score in all areas was greater than 60%. The ICC of the scores of the three reviewers for each domain of each guideline was greater than 0.8, indicating that the agreement among the reviewers was good. The details are shown in Table S3.<br>After evaluating the nine guidelines according to the AGREE REX scale, the overall scores of guidelines 3 and 6were > 70%, which was of high quality, and the overall scores of the other guidelines were ≥ 30%, which was of moderate quality. The details are shown in Table S4. (Page 8-9)<br>d. We summarised the similarities and differences in the guidelines for thromboprophylaxis associated with central venous catheters in table S5 and table S6.(Page 9-10)
**Discussion**	*What does it mean?*
**14. Summary**	a. Key findings : The overall quality of the nine guidelines included is good, and the corresponding advice and suggestions about CRT prevention are provided, but the guidelines still need to be improved in terms of rigorism and independence of writing, participants and applications. At present, there is still a lack of high-quality clinical practice guidelines for CRT prevention, and there is also a lack of dedicated CRT risk assessment tool. (Page 8-9)<br>b. Particular strengths of the project : NA

For peer review only

**15. Interpretation**	a. Nature of the association between the intervention(s) and the outcomes: NA<br>b. Comparison of results with findings from other publications: NA<br>c. Impact of the project on people and systems : NA<br>d. By evaluating the content and quality of these guidelines, it is found that there is currently a lack of high-quality clinical practice guidelines dedicated to CRT prevention at home and abroad, and there is currently a lack of risk assessment scales dedicated to CRT in clinical practice. We believe that it is urgent to develop clinical practice guidelines and risk assessment scales related to CRT prevention based on evidence-based concepts, so as to involve patients in research based on clinical status, reduce the incidence of CRT, and improve the level of CRT detection, management and prevention.(Page 15-16)<br>e. Costs and strategic trade-offs, including opportunity costs: NA
**16. Limitations**	The evidence in the guidelines included in this study is mainly from developed countries and some developing countries, and other countries need to be cautious when interpreting the results of this study
**17. Conclusions**	The overall quality of the nine guidelines included is good, and the corresponding advice and suggestions about CRT prevention are provided, but the guidelines still need to be improved in terms of rigorism and independence of writing, participants and applications. At present, there is still a lack of high-quality clinical practice guidelines for CRT prevention, and there is also a lack of dedicated CRT risk assessment tool. Therefore, there is an urgent need to develop evidence-based CRT prevention related clinical practice guidelines and risk assessment tools based on the clinical situation to enable patients to participate in the management of CRT, thereafter to to better manage the CRT.(Page 16)
**Other information**	
**18. Funding**	This work was supported by the Sichuan Science and Technology Program (Grant 2022NSFSC1290), and West China Nursing Discipline Development Special Fund Project, Sichuan University (Grant HXHL21011). (Page 17)

For peer review only

Table 2. Glossary of key terms used in SQUIRE 2.0. This Glossary provides the intended meaning of selected words and phrases as they are used in the SQUIRE 2.0 Guidelines. They may, and often do, have different meanings in other disciplines, situations, and settings.

Assumptions

Reasons for choosing the activities and tools used to bring about changes in healthcare services at the system level.

Context

Physical and sociocultural makeup of the local environment (for example, external environmental factors, organizational dynamics, collaboration, resources, leadership, and the like), and the interpretation of these factors ("sense-making") by the healthcare delivery professionals, patients, and caregivers that can affect the effectiveness and generalizability of intervention(s).

Ethical aspects

The value of system-level initiatives relative to their potential for harm, burden, and cost to the stakeholders. Potential harms particularly associated with efforts to improve the quality, safety, and value of healthcare services include opportunity costs, invasion of privacy, and staff distress resulting from disclosure of poor performance.

Generalizability

The likelihood that the intervention(s) in a particular report would produce similar results in other settings, situations, or environments (also referred to as external validity).

Healthcare improvement

Any systematic effort intended to raise the quality, safety, and value of healthcare services, usually done at the system level. We encourage the use of this phrase rather than "quality improvement," which often refers to more narrowly defined approaches.

Inferences

The meaning of findings or data, as interpreted by the stakeholders in healthcare services – improvers, healthcare delivery professionals, and/or patients and families

Initiative

A broad term that can refer to organization-wide programs, narrowly focused projects, or the details of specific interventions (for example, planning, execution, and assessment)

Internal validity

Demonstrable, credible evidence for efficacy (meaningful impact or change) resulting from introduction of a specific intervention into a particular healthcare system.

Intervention(s)

The specific activities and tools introduced into a healthcare system with the aim of changing its performance for the better. Complete description of an intervention includes its inputs, internal activities, and outputs (in the form of a logic model, for example), and the mechanism(s) by which these components are expected to produce changes in a system's performance.

Opportunity costs

For peer review only

Loss of the ability to perform other tasks or meet other responsibilities resulting from the diversion of resources needed to introduce, test, or sustain a particular improvement initiative

Problem

Meaningful disruption, failure, inadequacy, distress, confusion or other dysfunction in a healthcare service delivery systemthat adversely affects patients, staff, or the system as a whole, or that prevents care from reaching its full potential

Process

The routines and other activities through which healthcare services are delivered

Rationale

Explanation of why particular intervention(s)were chosen and why it was expected to work, be sustainable, and be replicable elsewhere.

Systems

The interrelated structures, people, processes, and activities that together create healthcare services for and with individual patients and populations. For example, systems exist from the personal self-care system of a patient, to the individual provider-patient dyad system, to the microsystem, to the macrosystem, and all the way to the market/social/insurance system. These levels are nested within each other.

Theory or theories

Any “reason-giving” account that asserts causal relationships between variables (causal theory) or that makes sense of an otherwise obscure processor situation (explanatory theory). Theories come in many forms, and serve different purposes in the phases ofimprovementwork. It is important to be explicit and well-founded about any informal and formal theory (or theories) that are used.

For peer review only

BMJ Open

Appraising the quality standard of clinical practice guidelines related to central venous catheter-related thrombosis prevention: a systematic review of clinical practice guidelines

Journal:	*BMJ Open*
Manuscript ID	bmjopen-2023-074854.R2
Article Type:	Original research
Date Submitted by the Author:	14-Feb-2024
Complete List of Authors:	Zhang, Jing; Sichuan University<br>Wu, Yongya; Sichuan University<br>Zhang, Shuai; Sichuan University<br>Yao, Wenmo; Sichuan University<br>Bu, Faqian; Sichuan University<br>Wang, Aoxue; Sichuan University<br>Hu, Xiuying; Sichuan University<br>Wang, Guan; Sichuan University
**Primary Subject Heading**:	Cardiovascular medicine
Secondary Subject Heading:	Cardiovascular medicine, Haematology (incl blood transfusion), Oncology
Keywords:	Thromboembolism < CARDIOLOGY, Systematic Review, VASCULAR MEDICINE, Anaesthesia in oncology < ANAESTHETICS

SCHOLARONE™
Manuscripts

I, the Submitting Author has the right to grant and does grant on behalf of all authors of the Work (as defined in the below author licence), an exclusive licence and/or a non-exclusive licence for contributions from authors who are: i) UK Crown employees; ii) where BMJ has agreed a CC-BY licence shall apply, and/or iii) in accordance with the terms applicable for US Federal Government officers or employees acting as part of their official duties; on a worldwide, perpetual, irrevocable, royalty-free basis to BMJ Publishing Group Ltd ("BMJ") its licensees and where the relevant Journal is co-owned by BMJ to the co-owners of the Journal, to publish the Work in this journal and any other BMJ products and to exploit all rights, as set out in our licence.

The Submitting Author accepts and understands that any supply made under these terms is made by BMJ to the Submitting Author unless you are acting as an employee on behalf of your employer or a postgraduate student of an affiliated institution which is paying any applicable article publishing charge ("APC") for Open Access articles. Where the Submitting Author wishes to make the Work available on an Open Access basis (and intends to pay the relevant APC), the terms of reuse of such Open Access shall be governed by a Creative Commons licence – details of these licences and which Creative Commons licence will apply to this Work are set out in our licence referred to above.

Other than as permitted in any relevant BMJ Author's Self Archiving Policies, I confirm this Work has not been accepted for publication elsewhere, is not being considered for publication elsewhere and does not duplicate material already published. I confirm all authors consent to publication of this Work and authorise the granting of this licence.

For peer review only

Appraising the quality standard of clinical practice guidelines related to central venous catheter-related thrombosis prevention: a systematic review of clinical practice guidelines

Jing Zhang[1], Yongya Wu[1], Shuai Zhang[1], Wenmo Yao[1], Faqian Bu[1], Aoxue Wang[1], Xiuying Hu[1], Guan Wang[1]

[1] Innovation Center of Nursing Research, Nursing Key Laboratory of Sichuan Province, State Key Laboratory of Biotherapy and Cancer Center, West China Hospital, Sichuan University /West China School of Nursing, Sichuan University, Chengdu 610041, China.

Correspondence to

Dr Guan Wang; guan8079@163.com

Dr. Xiuying Hu; huxiuying@scu.edu.cn

For peer review only

Abstract:

Objective To evaluate the quality and analyze the content of clinical practice guidelines regarding central venous catheter-related thrombosis to provide evidence for formulating an evidence-based practice protocol and a risk assessment scale to prevent it.

Design Scoring and analysis of the guidelines using the AGREE II and AGREE REX scales.

Data sources Pubmed, Embase, Cochrane Library, Web of Science, CNKI, Wanfang, VIP, and the Chinese Biomedical Literature, and the relevant websites of the guideline, were searched from Jan 1, 2017 to Mar 26, 2022.

Eligibility criteria Guidelines covering CRT treatment, prevention, or management were included from Jan 1, 2017, to Mar 26, 2022.

Data extraction and synthesis Three independent reviewers systematically trained in using the AGREE II and AGREE REX scales were selected to evaluate these guidelines.

Results Nine guidelines were included, and the quality grade results showed that three were at A-level and six were at B-level. The included guidelines mainly recommended the prevention measure of central venous catheter-related thrombosis from three aspects: risk screening, prevention strategies, and knowledge training, with a total of 22 suggestions being recommended.

Conclusion The overall quality of the guidelines is high, but there are few preventive measures for central venous catheter-related thrombosis involved in the guidelines. All preventive measures have yet to be systematically integrated and evaluated, and no risk assessment scale dedicated to this field has been recommended. Therefore, developing an evidence-based practice protocol and a risk assessment scale to prevent it is urgent.

Keywords: Central venous catheter; Catheter-related thrombosis; Venous thromboembolism; Clinical practice guidelines; Prevention

Strengths and limitations of this study

- Two quality evaluation tools, AGREE II and AGREE REX, were used to comprehensively and rigorously evaluate the quality of CVC-related clinical guidelines.
- This review clarifies the CVC guidelines with high evidence strength and the

For peer review only - http://bmjopen.bmj.com/site/about/guidelines.xhtml

highest clinical applicability and provides a reference for clinical medical personnel to select the best guidelines.

- The evidence in the guidelines included in this study is mainly from developed countries and some developing countries, and other countries need to be cautious when interpreting the results of this study.
- Only guidance documents in English and Chinese were included, so we cannot rule out the possibility that some guidelines may have been omitted from this study.

For peer review only

Introduction

The central venous catheter is inserted through the proximal central or peripheral vein, with the catheter tip located at one-third of the junction between the superior vena cava and the right atrium(1). The CVC includes subclavian vein/ jugular vein/femoral vein puncture catheters, peripherally inserted central catheter(PICC), and implantable venous access port(2). It has many advantages, such as convenient and safe operation, rapid establishment of intravenous access, reduced puncture times, and infusion of irritating liquid; therefore, it has been widely used in chemotherapy, hemodialysis, nutrition support, and surgery support(3–6).

Catheter-related thrombosis(7) refers to the formation of blood clots on the outer wall or inner wall of a catheter. CRT is one of the common complications after catheterization of CVC, which can lead to unplanned extubation and pulmonary embolism, affecting the progress of treatment and increasing the cost of treatment(8–11). The risk factors of CRT could be categorized into patient-related, disease/treatment-related, and catheter-related factors. Patient-related factors include sex, advanced age, long-term bedridden, venous thromboembolism history, and acquired or hereditary blood hypercoagulability state(12–14). Diseases/treatment-related factors include the grade of cancer, chemotherapy, radiotherapy, parenteral nutrition support, surgical trauma, or excessive bleeding (15). Catheter-related factors include catheter material, catheter-to-vein ratio, puncture times, catheter indwelling length, and the catheter tip's location(16).

For peer review only - http://bmjopen.bmj.com/site/about/guidelines.xhtml

CRT has the characteristics of high incidence, early onset, and clinically silent symptoms(17). The clinical manifestations of CRT are as follows: deep venous thrombosis(18) (DVT), superficial thrombophlebitis, asymptomatic thrombosis, and thrombotic catheter failure. The study reported that CRT incidence ranged from 1.9 to 73.0%(5,9,16,17,19). It could happen as early as the first day after catheterization(17,20), and the average time of occurrence was within two weeks after catheterization(5,11,21,22). However, the early symptoms and signs were silent; only a few patients showed clinical symptoms. Due to the limited medical resources, vascular surgery in most medical institutions in China can not monitor the occurrence of CRT in time. It can not correctly prevent and deal with CRT, increasing hospitalization time, resulting in irreversible effects, and affecting the quality of follow-up treatment of patients.

Therefore, assessing and taking measurements early to prevent patients at high risk of CRT are essential to ensure clinical workers formulate their safety. Clinical Practice guidelines after systematically integrating and evaluating relevant research and clinical experience according to evidence-based concepts to a particular common symptom, disease, or problem, to provide guiding suggestions for clinical workers and patients, and scientifically promote the application of evidence in clinical practice(23). Several clinical practice guidelines about CRT prevention have been published, but their quality could be more apparent. Therefore, through the systematic retrieval, screening, and reading of clinical practice guidelines related to CRT prevention and the quality evaluation and content analysis of the guidelines, this review was expected to provide a scientific basis for developing evidence-based practice protocols and risk assessment scales for CRT.

Information and methods

Search strategy

Search databases

We searched English and Chinese databases, including Pubmed, Embase,

For peer review only - http://bmjopen.bmj.com/site/about/guidelines.xhtml

Cochrane Library, Web of Science, CNKI, Wanfang, VIP, and the Chinese Biomedical Literature Database from Jan 1, 2017 to Mar 26, 2022. In addition, the relevant websites of the guideline, including the National Institute for Health and Care Excellence (NICE) and Medline, were searched. A manual search of relevant studies and bibliographies was also adopted.

Search strategies

The literature retrieval adopted a strategy of the combination of MeSH terms and free words. The MeSH terms and synonyms of catheter-related thrombosis and clinical practice guidelines were searched in English and Chinese databases. Take Pubmed as the retrieval representative of the English database. The search strategy is shown in Supplementary Materials - Search strategies.

Inclusion and exclusion criteria

The inclusion criteria were (1) guidelines including the contents of treatment, prevention, or management of CRT; (2) guidelines published or updated after Jan 1, 2017; and (3) guidelines published in English or Chinese. The exclusion criteria were (1) guidelines developed by expert consensus exclusively, (2) incomplete guidelines that are in the process of development, and (3) the translation or reinterpreting of the guidelines.

Guideline screening and data extraction

Two researchers who had received systematic literature retrieval and training searched the literature. The searched literature was imported into Endnote X9 software, and the duplicates were removed. Then, two researchers independently read the title and abstracts for preliminary screening to exclude unrelated articles. Subsequently, two reviewers read the full text carefully and screened it again according to the established criteria for inclusion and exclusion. In case of disagreement, the third party will be consulted before determining. Finally, a predefined data extraction table was used to extract the data from the included studies.

Quality appraisal

For peer review only - http://bmjopen.bmj.com/site/about/guidelines.xhtml

The AGREE II scale(24), a widely used tool for evaluating the quality of the guideline, was used to evaluate the included guidelines. It includes six domains, i.e., scope and purpose, rigorism, independence, clarity, application, and independence of writing, with 23 items. Each item has a maximum score of seven and a minimum of one. Seven indicates that the evaluator is fully satisfied with the area covered by the entry, and one indicates that the evaluator is entirely dissatisfied with the field. The higher the evaluator is satisfied with the area covered by the guideline, the higher the scores. The evaluator should score the satisfaction degree of the guideline according to the actual situation to ensure the fairness of the guideline. Three researchers who systematically trained the use methods of the AGREE II scale were selected to evaluate the guidelines to ensure that the researchers had a complete understanding and consistent understanding of the items and to reduce the biases of evaluation. If there is a dispute over an item, experts in the relevant field would be consulted to resolve it. The standard rate of the three researchers in each field of the guideline was calculated, and the systematic evaluation content analysis and summary of the guideline were carried out based on the evidence-based concept. Level A recommended the guideline, indicating that most entries in the guideline scored ≥ 5, and its standardized percentage was ≥ 60%. Level B was recommended after modification and indicated that the standard rate in more than three areas was 30-60%. Level C indicated that the guideline was not recommended and that the standardized rate in three or more areas was less than 30%.

To evaluate the quality and applicability of each recommendation, improve the comparability of the results, and increase the effectiveness of the evaluation process, the AGREE REX scale(25) was used to evaluate the included guidelines. Three researchers systematically trained using the AGREE REX scale and evaluated the guidelines. The scores of the three researchers in each area of the guidelines were standardized to calculate. The guidelines were systematically evaluated, and content was analyzed and summarized based on the evidence-based concept. Referring to the recommendations of the AGREE REX manual, the guidelines define an overall score of >70% as high quality, an overall score of <30% as low quality, and others as

For peer review only - http://bmjopen.bmj.com/site/about/guidelines.xhtml

moderate quality.

Statistical methods

SPSS22.0 was used to count the scores of the three researchers in each field of the guideline, and the Intraclass Correlation Coefficient (ICC) of the data was calculated. According to the results, the consistency of the two researchers' guideline evaluation was tested. ICC < 0.4 indicated poor consistency, $0.40 \leq 0.59$ indicated that the consistency was general, $0.60 \leq 0.74$ indicated good consistency, and > 0.74 indicated perfect consistency.

Patient and public involvement

No patients or members of the public were involved in the development of the research question, the design or the conduct of the study, or the interpretation and writing up of results.

Result

Guideline retrieval results

A total of 2092 articles were retrieved, and 571 were removed by software and manual deletion. After reading the title and abstract, 1142 articles were excluded. 379 articles were screened according to inclusion and exclusion criteria. 267 articles that did not meet the article's theme were excluded, 5 related guidelines were traced through the snowball strategy, and 117 articles were finally selected. 11 articles were not obtained, and 106 complete articles were obtained. The full-text screening excluded 18 non-evidence-based guidelines issued in individual form, six guidelines that had been updated, and 73 guidelines that did not include CRT treatment, prevention, and management. Finally, nine literature were selected, including 2 Chinese literature and 7 English literature. The specific screened process is shown in Figure S1.

For peer review only - http://bmjopen.bmj.com/site/about/guidelines.xhtml

Essential characteristics of inclusion in the guideline

The nine guidelines were from the American Society of Clinical Oncology, the International Initiative on Thrombosis and Cancer, the Chinese Medical Association, the Spanish Medical Oncology Society, the Infusion Nursing Association, and other international authoritative medical associations. Only one guideline targets CRT risk assessment, prevention, and intervention(20). Five guidelines were specifically for venous thrombosis prevention in cancer patients(15,26–29), and the content related to CRT was only part of it. Two are guidelines for ordinary patients' venous thrombosis (26,30). CRT patients are part of the content, and only one guideline targets clinical infusion therapy(30). CRT is part of the treatment (see Table S1).

Results of AGREE II and AGREE REX

The included guidelines used AGREE II and AGREE-REX to evaluate the quality of methodologies and recommendations, respectively. After scoring the nine guidelines on the AGREE II scale, it was found that three guidelines(26,31,32) were recommended as A, and six guidelines(27,29,33–36) were recommended as B, as shown in Table S2. The average score in all areas was more than 60% excellent. The ICC of the scores of the three reviewers for each domain of each guideline was more significant than 0.8, indicating that the agreement among the reviewers was good. Specifically, Area 1 ("Scope and Purpose") addresses the general scope and purpose of CPGS, the specific clinical problems that need to be addressed, and the target population. The average score was 66.67%, of which eight articles scored more than 60% and five papers scored more than 70%. Area 2 ("Participants") reflects whether the normative process includes input and participation from relevant stakeholders. The average score was 60.70%, and 6 of the articles scored above the average score. Regarding "rigor" (area 3), the average score was 61.88%, with seven guidelines scoring above the average. Guideline 4(35) has the lowest scores in the three areas of scope and purpose, who is involved, and rigor, while most of the rest describe the development process, albeit with varying levels of detail.

Area 4 ("Clarity") focuses on the specificity and clarity of these items, including the

For peer review only - http://bmjopen.bmj.com/site/about/guidelines.xhtml

clear articulation of options for health management and making critical recommendations that are easy to identify. With an average score of 64.81%, there was the slightest difference in all guideline scores in this area, even though guideline scores 1, 2, 8, and 9 were below the average. "Application" (area 5) refers to facilitators and obstacles to implementing recommendations within the context of the guidelines, strategies for their implementation, and possible resource implications. The CPGS scored an average of 60.49 % in this area, with three scores below 60%. Finally, the sixth area ("independence") aims to ensure a lack of bias in developing guidelines. The score in this area was relatively good, with a median CPGS of 63.58%, three above 70%, and seven above 60%: most described funding, potential impact, or associated conflicts of interest. The guidelines scored slightly worse than the others in the areas of participants, rigor of development, applicability, and editorial independence.

After evaluating the nine guidelines according to the AGREE REX scale, the overall scores of guidelines 3(34) and 6(32) were > 70%, which was of high quality, and the overall scores of the other guidelines were ≥ 30%, which was of moderate quality. (see Table S2)The three reviewers agreed well, with an ICC value greater than 0.8, and each guideline scored slightly lower than the implement ability domain in the values and preference domains and clinical practicality. In clinical applicability, guideline 6(32) scored the highest, 83.33%, with a large score difference between guidelines. guideline 1(33) had the lowest score of 42.59%, as detailed in Table S2.

Comparative analysis of the specific content of CRT preventive measures in the guidelines

After reading all the recommendations and precautions included in the guidelines, it was found that the content of CRT prevention can be mainly divided into three aspects: risk assessment, preventive measures, and health education. The specific content and methods are shown in Table S3. Due to differences in the topic of the guidelines and the study participants, the focus and level of detail of the prevention strategies recommended for CRT vary among the guidelines. Therefore, for analysis and comparison, the relevant contents are summarized in Table S4.

For peer review only - http://bmjopen.bmj.com/site/about/guidelines.xhtml

Discussion

Essential characteristics of CRT Prevention guidelines

The guidelines included in the study are widely used and have high impact factors. Guidelines issued by the China Branch of the International Union of Angiology, for the first time, the preventive measures of CRT were systematically summarized, which filled the gap in this field. However, the methodological quality could have been better. Guidelines issued by ASCO, SEOM, ASH, NCCN, ITAC-CME(15,26,27,32). This paper mainly expounds on the preventive measures of CRT from two aspects of drug prevention and CVC-related prevention, including the choice of anticoagulant drugs, the time of continuous anticoagulant treatment, and the choice of catheter location; however, these were only a tiny part of the guideline, and the content was scattered in the included guidelines. Five included guidelines were targeted at cancer patients and had certain limitations. Guidelines issued by the Chinese Medical Association and NICE(26,30) mainly put forward some suggestions on how to prevent various types of thrombosis, and the prevention measures for CRT were one of them, including drug prevention of CRT, CVC catheter-related prevention, and physical prevention. The guidelines issued by INS describe the latest clinical infusion treatment practice standards and specific measures(30). The prevention of CVC-related complications was part of them, mainly describing how to deal with CRT.

Quality evaluation of the guidelines

To ensure the quality of the guidelines, researchers should follow the principles of professionalism, fairness, evidence-based and transparency when developing the guidelines. As the guidelines included in this study are of good quality and high impact factors, the AGREE II scale is used to evaluate the six areas of the guideline. The guidelines' clarity, scope, and purpose have reached the standard level. In contrast, the rigorism and independence, participants, and application of the guidelines must be further improved. The specific analysis was as follows:

rigorism and independence

For peer review only - http://bmjopen.bmj.com/site/about/guidelines.xhtml

Regarding rigorism, the expert consensus was released by the China Branch of the International Union of Angiology. The guideline does not systematically retrieve CRT prevention-related research but only summarizes the "experience" of clinical experts, lack of corresponding evidence support, and lack of scientific and rigorous. The guidelines issued by NCCN and NICE(26,29),1/1/0001 12:00:00 AM systematically evaluated the relevant research and conclusions and recommended opinions and suggestions. However, these two guidelines should have pointed out the steps and methods of systematic retrieval of evidence and the criteria for accepting and excluding the research. In addition to the guidelines issued by INS(30), the rest do not specify the steps and methods for updating the guidelines. However, the guidelines indicated the timing of the update(30) and the extent to which the evidence level of the new guideline should be higher than that of the previous edition. They indicated that the quality of the evidence in the guideline is constantly improving. Due to the differences in medical and health systems at home and abroad and the non-standard evaluation of guidelines, the study found that most of the guidelines published abroad put forward statements of interest in the study(15,26,27,29,30,32). However, the guideline issued by the China Branch of the International Union of Angiology failed to state the study's statement of interest. The interests of the panel members with other bodies are also not recorded, and the fairness and independence of the study are not guaranteed. Therefore, we should pay full attention to the rigor and independence of the guideline, pursue evidence according to the concept of evidence-based medicine, and avoid the influence of external factors and research biases caused by personal preferences to ensure the rigor and independence of research.

participants

In the participant section, only two guidelines issued by the Chinese Medical Association and INS(27,30), the names and work units of all the experts involved in the formulation of the guidelines are listed, while the rest of the guidelines refer only to interdisciplinary teams or association support and without specify the relevant information of the researchers. In addition to the release guidelines of the Chinese Medical Association, NCCN, and ITAC-CME(23,25,30), the rest of the team did not

For peer review only - http://bmjopen.bmj.com/site/about/guidelines.xhtml

explain how to collect comments and suggestions from users considering the relevant information of researchers, such as work experience, units, and professional titles, can reduce the bias caused by personal factors of researchers, which is conducive to the formulation of guidelines and reflects the universality and authority of the formulation of guidelines.

application

Relevant studies have shown that the use of clinical guidelines could be much higher in the field of application. Most of the guidelines in this study need to fully consider the difficulties and obstacles encountered in the follow-up application and the relevant systems and documents accompanying them. There needs to be training for clinical staff in using the guidelines, which is not conducive to learning and using the guidelines. Of the included guidelines, only this issued by NICE is presented(30). The guidelines are applied to specific clinical methods and measures. However, the rest of the guidelines must address the forward and reverse factors in applying the guidelines, how to use potential resources, and how to transform the theory into practice. Therefore, it is necessary to carry out pre-experiments in the process of guideline formulation in the future, check the feasibility of guideline recommendations in advance, collect the problems in applying guidelines in advance, make corresponding preparations, and promote the transformation of guidelines to clinical practice.

analysis of the recommendations in the guidelines

risk screening

Timely assessment of risk factors for CRT formation in patients is beneficial to the clinical prevention of CRT formation. In addition to the guidelines issued by SEOM and ITAC-AEM(15,27), the rest indicate that CRT risk factors must be assessed before CVC intubation. The guidelines also clearly point out the high-risk factors of CRT, mainly divided into patient-, catheter-, and disease/treatment-related factors. The patient-related factors were age, sex, family history or personal history of venous thrombosis, hypercoagulable state of blood, and abnormal coagulation gene. Catheter-related factors were catheter material, placement technique, location and type of

For peer review only - http://bmjopen.bmj.com/site/about/guidelines.xhtml

For peer review only

catheter, and puncture times. Disease/treatment-related factors were antiangiogenic therapy, hormone therapy, parenteral nutrition, radiotherapy, tumor and trauma, and complicated surgery. However, there is yet to be a recognized scale dedicated to CRT risk assessment. Most of the guidelines recommend using the VTE risk factor assessment scale, and the two guidelines recommend using the Khorana scale and Wells scale(22,23,30). The guideline issued by NCCN recommends that patients with medical diseases can use the Padua scale(29). At the same time, it is suggested that myeloma patients should be treated with targeted anticoagulant therapy after scoring with IMPED and SAVED scales. CRT risk assessment should be conducted at admission, before and after catheterization and replacement, and when symptoms and signs occur. Timely evaluation of CRT-related symptoms of patients, regular examination of related physiological and biochemical indicators according to the actual situation, flexible adjustment of anticoagulant program, and the timely report of the occurrence of particular circumstances.

Prevention strategies

Through the summary and analysis of the nine guidelines, it is found that the preventive measures are mainly divided into the following five parts: classified prevention, physical prevention, drug prevention, standard CRT prevention measures, and multi-disciplinary prevention. Classified prevention emphasizes individualized prevention according to the patients' factors and the clinical manifestations of CRT. Physical prevention is mainly to help patients exercise actively or passively, promote their blood circulation, and reduce thrombosis formation. To urge patients to get out of bed as soon as possible, put tube side limbs to perform functional exercises such as repeated clenching or loosening, promote blood circulation, replenish water, and prevent dehydration and blood coagulation. However, most guidelines indicate that patients at risk of post-thrombosis syndrome do not recommend routine use of elastic socks for prevention, as they may aggravate skin breakage and injury. Drug prevention mainly uses low-dose unfractionated heparin, low molecular weight heparin, and vitamin K antagonists, while some guidelines recommend the use of a new direct oral anticoagulant, DOAC. The risk of bleeding should be considered when anticoagulants

For peer review only - http://bmjopen.bmj.com/site/about/guidelines.xhtml

are used, and the risks and benefits of the drug should be assessed and used with the patient's informed consent. The common prevention of CRT is mainly the treatment of a catheter, including the choice of catheter diameter and lumen, the way of catheterization, the location of catheterization, the time of catheterization, and subsequent anticoagulant therapy, as shown in tables S4 and S5. Multi-disciplinary prevention refers to constructing a multi-disciplinary team composed of vascular surgery, oncology, evidence-based medicine centers, medical laboratory technicians, and nurses to carry out CRT prevention to avoid the limitations of clinical practice.

knowledge training

The guideline issued by INS suggests that nurses should constantly update their clinical knowledge and skills, and based on the evidence-based transformation of the latest research into high-quality evidence, to promote its transformation to clinical practice. Currently, the update of clinical nursing is slow, and most medical staff are still based on traditional nursing methods, so it is not easy to introduce new research results into clinical practice. Therefore, it is necessary to carry out systematic scientific research education and organize reading exchange meetings and knowledge training for nurses. The guidelines issued by ASCO(26) indicate that, although CRT is closely related to cancer, patients' awareness of CRT's risks and warning signals is still deficient, emphasizing the need to increase patient education and awareness(28,37). At the same time, a study suggests that patients may not report new symptoms unless asked directly, as they usually consider symptoms to be a manifestation of cancer or a side effect of treatment. Therefore, health education for patients is helpful to ensure effective communication between medical staff and patients and is conducive to patients' understanding of the disease(38).

Limitations of the guidelines.

Although one of the nine guidelines in this study is a specific guideline on CRT prevention, it needs to retrieve relevant research and evidence systematically. It only recommends and compiles guidelines based on the experience of clinical experts, which need more scientific. Most other guidelines are suitable for cancer patients, and the

For peer review only - http://bmjopen.bmj.com/site/about/guidelines.xhtml

primary prevention strategies related to CRT are drug prevention and CVC catheter prevention. However, their contents are fewer, their views are scattered, the subjects of the guidelines are limited, and there needs to be a more systematic, integrated evaluation. Therefore, the formulation of evidence-based CRT prevention-related clinical practice guidelines is helpful to guideline clinical staff in standardizing the operation and ensuring the treatment quality of patients. At the same time, after analyzing the guideline's content, it is found that no tool is dedicated to CRT risk assessment. Presently, the guidelines recommend using a mature VTE risk assessment scale. However, this kind of scale does not involve the evaluation of the CVC catheter, which is not targeted, which may lead to a measurement bias. Therefore, developing a risk assessment scale for CRT is conducive to accurately selecting appropriate preventive measures and anticoagulant treatment methods and reduces the injury caused to patients after CRT.

In summary, the quality of the nine clinical guidelines included in this study is moderately high, but there are some differences in the guidelines in different regions and countries. The guidelines need to be further improved in the following aspects: (1) there is no risk assessment scale dedicated to CRT, and a targeted evaluation form should be developed based on evidence-based evidence, which is conducive to the accurate selection of appropriate preventive measures and anticoagulation treatments; (2) clinical practice guidelines and risk assessment scales related to CRT prevention based on evidence-based concepts should be formulated to enable patients to participate in the study based on the clinical status quo to increase the practicability of the guidelines.

Conclusion

The quality of the nine guidelines is good, and the corresponding advice and suggestions about CRT prevention are provided. However, the guidelines still need to be improved regarding the rigor and independence of writing, participants, and

For peer review only - http://bmjopen.bmj.com/site/about/guidelines.xhtml

applications. At present, there is still a need for high-quality clinical practice guidelines for CRT prevention, and there is also a need for dedicated CRT risk assessment tools. Therefore, there is an urgent need to develop evidence-based CRT prevention-related clinical practice guidelines and risk assessment tools based on the clinical situation to enable patients to participate in the management of CRT and, after that, to better manage CRT.

Contributors

GW and XH conceived and supervised the project. JZ, YW, and SZ summarized the literature and drafted the manuscript. WY, FB, and AW drew the figures and tables. GW and JZ revised the manuscript. All authors contributed, edited, and approved the final manuscript.

Funding

This work was supported by the Sichuan Science and Technology Program (Grant 2022NSFSC1290) and the West China Nursing Discipline Development Special Fund Project, Sichuan University (Grant HXHL21011).

Competing interests

The authors declare that they have no conflicts of interest.

Patient and public involvement

Patients and the public were not involved in this research's design, conduct, reporting, or dissemination plans.

Patient consent for publication

Not required

For peer review only - http://bmjopen.bmj.com/site/about/guidelines.xhtml

Provenance and peer review

Not commissioned; externally peer-reviewed

Ethics approval

Not involved.

Data sharing statement

No additional data available

Supplemental material

This content has been supplied by the author(s). It has not been vetted by BMJ Publishing Group Limited (BMJ) and may not have been peer-reviewed. Any opinions or recommendations discussed are solely those of the author(s) and are not endorsed by BMJ. BMJ disclaims all liability and responsibility arising from any reliance on the content. Where the content includes any translated material, BMJ does not warrant the accuracy and reliability of the translations (including but not limited to local regulations, clinical guidelines, terminology, drug names, and drug dosages) and is not responsible for any error or omissions arising from translation and adaptation or otherwise.

Open access

This is an open-access article distributed in accordance with the Creative Commons Attribution Non Commercial (CC BY-NC 4.0) license, which permits others to distribute, remix, adapt, build upon this work non-commercially, and license their derivative works on different terms, provided the original work is properly cited, appropriate credit is given, any changes made indicated, and the use is non-commercial. See: http://creativecommons.org/licenses/by-nc/4.

For peer review only

References

1. Smith RN, Nolan JP. Central venous catheters. BMJ. 2013 Nov 11;347:f6570.
2. Expert Group on Safety Management of Central Venous Access Devices. Zhonghua Wai Ke Za Zhi. 2020 Apr 1;58(4):261–72.
3. Matar CF, Kahale LA, Hakoum MB, Tsolakian IG, Etxeandia-Ikobaltzeta I, Yosuico VE, et al. Anticoagulation for perioperative thromboprophylaxis in people with cancer. Cochrane Database Syst Rev. 2018 Jul 11;7(7): CD009447.
4. Hao N, Xie X, Zhou Z, Li J, Kang L, Wu H, et al. A nomogram predicted the peripherally inserted central catheter-related thrombosis risk. Sci Rep. 2017 Jul 24;7(1):6344.
5. Liu Y, Gao Y, Wei L, Chen W, Ma X, Song L. Peripherally inserted central catheter thrombosis incidence and risk factors in cancer patients: a double-center prospective investigation. Ther Clin Risk Manag. 2015;11:153–60.
6. Meyer BM. Developing an alternative workflow model for peripherally inserted central catheter placement. J Infus Nurs. 2012;35(1):34–42.
7. Falanga A, Ay C, Di Nisio M, Gerotziafas G, Jara-Palomares L, Langer F, et al. Venous thromboembolism in cancer patients: ESMO Clinical Practice Guideline. Ann Oncol. 2023 May;34(5):452–67.
8. Takemoto CM, Sohi S, Desai K, Bharaj R, Khanna A, McFarland S, et al. Hospital-associated venous thromboembolism in children: incidence and clinical characteristics. J Pediatr. 2014 Feb;164(2):332–8.
9. Chen Y, Chen H, Yang J, Jin W, Fu D, Liu M, et al. Patterns and risk factors of peripherally inserted central venous catheter-related symptomatic thrombosis events in patients with malignant tumors receiving chemotherapy. J Vasc Surg Venous Lymphat Disord. 2020 Nov;8(6):919–29.
10. Chopra V, Anand S, Krein SL, Chenoweth C, Saint S. Bloodstream infection, venous thrombosis, and peripherally inserted central catheters: reappraising the evidence. Am J Med. 2012 Aug;125(8):733–41.
11. Marnejon T, Angelo D, Abu Abdou A, Gemmel D. Risk factors for upper extremity venous thrombosis associated with peripherally inserted central venous catheters. J Vasc Access. 2012;13(2):231–8.
12. Chopra V, Anand S, Hickner A, Buist M, Rogers MA, Saint S, et al. Risk of venous thromboembolism associated with peripherally inserted central catheters: a systematic review and meta-analysis. Lancet. 2013 Jul 27;382(9889):311–25.
13. Lowell JA, Bothe A. Central venous catheter-related thrombosis. Surg Oncol Clin N Am. 1995 Jul;4(3):479–92.
14. Tran BH, Nguyen TJ, Hwang BH, Vidar EN, Davis GB, Chan LS, et al. Risk factors associated with venous thromboembolism in 49,028 mastectomy patients. Breast. 2013 Aug;22(4):444–8.
15. Ferro JM, Bousser MG, Canhão P, Coutinho JM, Crassard I, Dentali F, et al. European Stroke Organization guideline for diagnosing and treating cerebral venous

For peer review only

For peer review only - http://bmjopen.bmj.com/site/about/guidelines.xhtml

thrombosis - endorsed by the European Academy of Neurology. Eur J Neurol. 2017 Oct;24(10):1203–13.
16. Jones D, Wismayer K, Bozas G, Palmer J, Elliott M, Maraveyas A. The risk of venous thromboembolism associated with peripherally inserted central catheters in ambulant cancer patients. Thromb J. 2017;15:25.
17. Chen H, Tao L, Zhang X, Jing W, Su X, Chen H, et al. The effect of systemic and local risk factors on triggering peripherally inserted central catheter-related thrombosis in cancer patients: A prospective cohort study based on ultrasound examination and structural equation modeling. Int J Nurs Stud. 2021 Sep;121:104003.
18. Duffett L. Deep Venous Thrombosis. Ann Intern Med. 2022 Sep;175(9): ITC129–44.
19. Ahn DH, Illum HB, Wang DH, Sharma A, Dowell JE. Upper extremity venous thrombosis in patients with cancer with peripherally inserted central venous catheters: a retrospective analysis of risk factors. J Oncol Pract. 2013 Jan;9(1):e8-12.
20. Wang G, Li Y, Wu C, Guo L, Hao L, Liao H, et al. The clinical features and factors of PICC-related upper extremity asymptomatic venous thrombosis in cancer patients: A prospective study. Medicine (Baltimore). 2020 Mar;99(12):e19409.
21. Bonizzoli M, Batacchi S, Cianchi G, Zagli G, Lapi F, Tucci V, et al. Peripherally inserted central venous catheters and central venous catheter-related thrombosis in post-critical patients. Intensive Care Med. 2011 Feb;37(2):284–9.
22. Chopra V, Kaatz S, Grant P, Swaminathan L, Boldenow T, Conlon A, et al. Risk of Venous Thromboembolism Following Peripherally Inserted Central Catheter Exchange: An Analysis of 23,000 Hospitalized Patients. Am J Med. 2018 Jun;131(6):651–60.
23. Institute of Medicine (US) Committee to Advise the Public Health Service on Clinical Practice Guidelines. Clinical Practice Guidelines: Directions for a New Program. Field MJ, Lohr KN, editors. Washington (DC): National Academies Press (US); 1990. Available from: http://www.ncbi.nlm.nih.gov/books/NBK235751/
24. Brouwers MC, Kho ME, Browman GP, Burgers JS, Cluzeau F, Feder G, et al. AGREE II: Advancing health care guideline development, reporting, and evaluation. Preventive Medicine. 2010 Nov 1;51(5):421–4.
25. Development and Validation of a Tool to Assess the Quality of Clinical Practice Guideline Recommendations. JAMA Netw Open. 2020;3(5):e205535. Published 2020 May 1
26. Key NS, Khorana AA, Kuderer NM, Bohlke K, Lee AYY, Arcelus JI, et al. Venous Thromboembolism Prophylaxis and Treatment in Patients With Cancer: ASCO Clinical Practice Guideline Update. J Clin Oncol. 2020 Feb 10;38(5):496–520.
27. Farge D, Frere C, Connors JM, Ay C, Khorana AA, Munoz A, et al. 2019 international clinical practice guidelines for the treatment and prophylaxis of venous thromboembolism in patients with cancer. Lancet Oncol. 2019 Oct;20(10):e566–81.
28. Sousou T, Khorana AA. Cancer patients and awareness of venous thromboembolism. Cancer Invest. 2010 Jan;28(1):44–5.
29. Streiff MB, Holmstrom B, Angelini D, Ashrani A, Elshoury A, Fanikos J, et al. Cancer-Associated Venous Thromboembolic Disease, Version 2.2021, NCCN

For peer review only

Clinical Practice Guidelines in Oncology. J Natl Compr Canc Netw. 2021 Oct 15;19(10):1181–201.
30. Hinck SM. Implementing the Infusion Therapy Standards of Practice. Home Health Now. 2021 Oct 1;39(5):295.
31. Venous thromboembolism in over 16s: reducing the risk of hospital-acquired deep vein thrombosis or pulmonary embolism London: National Institute for Health and Care Excellence (NICE); 2019. (National Institute for Health and Care Excellence: Guidelines).
32. Witt DM, Nieuwlaat R, Clark NP, Ansell J, Holbrook A, Skov J, et al. American Society of Hematology 2018 guidelines for management of venous thromboembolism: optimal management of anticoagulation therapy. Blood Adv. 2018 Nov 27;2(22):3257–91.
33. Chinese Expert Consensus on the Prevention and Treatment of Infusion Catheter-related Venous Thrombosis (2020 Edition) [J]. Chinese Journal of Practical Surgery,2020,40(4):377-383.
34. Guidelines for preventing and treating thrombotic diseases in China - Chinese Medical Journal. National Medical Journal of China. 2018 Sep 25;98(36):2861–88.
35. SEOM clinical guideline of venous thromboembolism (VTE) and cancer (2019) - PubMed.
36. Infusion Therapy Standards of Practice, 8th Edition : Journal of Infusion Nursing.
37. Aggarwal A, Fullam L, Brownstein AP, Maynard GA, Ansell J, Varga EA, et al. Deep vein thrombosis (DVT) and pulmonary embolism (PE): awareness and prophylaxis practices reported by patients with cancer. Cancer Invest. 2015;33(9):405–10.
38. Gilligan T, Coyle N, Frankel RM, Berry DL, Bohlke K, Epstein RM, et al. Patient-Clinician Communication: American Society of Clinical Oncology Consensus Guideline. J Clin Oncol. 2017 Nov 1;35(31):3618–32.

For peer review only

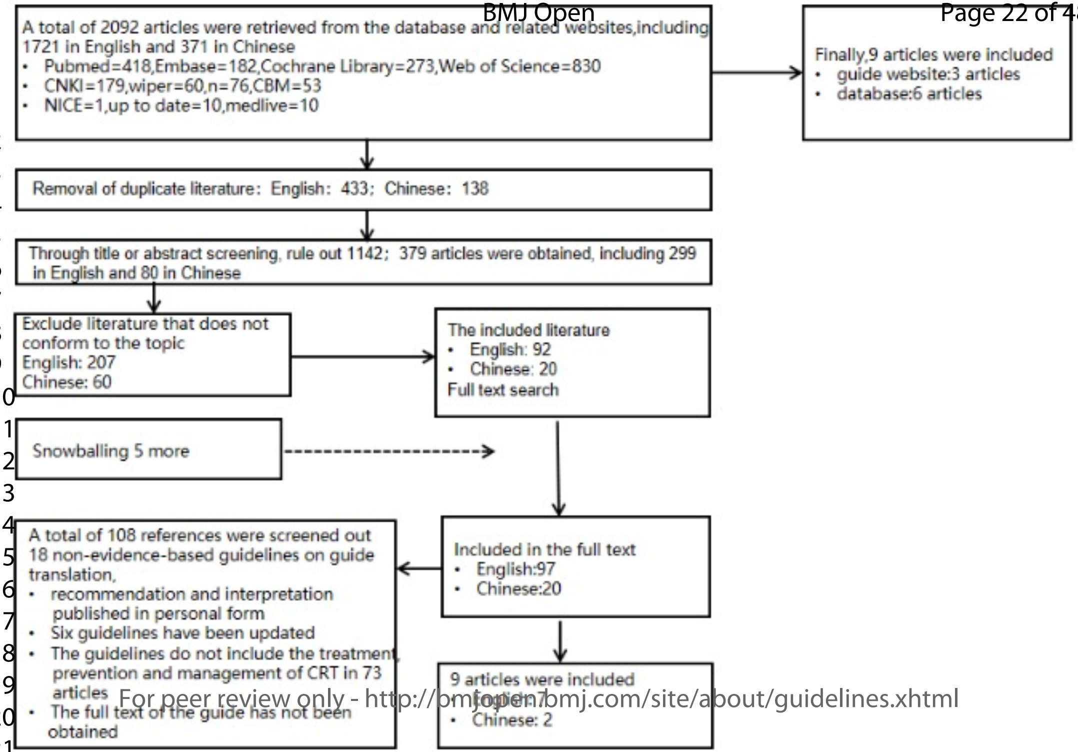
A total of 2092 articles were retrieved from the database and related websites,including 1721 in English and 371 in Chinese
• Pubmed=418,Embase=182,Cochrane Library=273,Web of Science=830
• CNKI=179,wiper=60,n=76,CBM=53
• NICE=1,up to date=10,medlive=10
Finally,9 articles were included
• guide website:3 articles
• database:6 articles
Removal of duplicate literature: English: 433; Chinese: 138
Through title or abstract screening, rule out 1142; 379 articles were obtained, including 299 in English and 80 in Chinese
Exclude literature that does not conform to the topic
English: 207
Chinese: 60
The included literature
• English: 92
• Chinese: 20
Full text search
Snowballing 5 more
A total of 108 references were screened out 18 non-evidence-based guidelines on guide translation,
• recommendation and interpretation published in personal form
• Six guidelines have been updated
• The guidelines do not include the treatment, prevention and management of CRT in 73 articles
• The full text of the guide has not been obtained
Included in the full text
• English:97
• Chinese:20
9 articles were included
• Chinese: 2

Table S1. Includes the basic characteristics table of the guideline

Number	Name	Year	Publishing agency	Subject	language	Whether or not evidence-based guidelines	Number of references
1	Chinese Expert Consensus on the Prevention and Treatment of Infusion Catheter-related Venous Thrombosis (2020 Edition)	2020	Vascular Alliance China Chapter	Catheter-related thrombosis prevention	Chinese	Not	62
2	Venous Thromboembolism Prophylaxis and Treatment in Patients With Cancer: ASCO Clinical Practice Guideline Update	2019	American Society of Clinical Oncology	Prevention of cancer-related blood clots	English	Yes	155
3	Guidelines for the prevention and treatment of thrombotic diseases in China	2018	Chinese Medical Association	Diagnosis and prevention of thrombosis	Chinese	Not	41
4	SEOM clinical guideline of venous thromboembolism (VTE) and cancer	2019	Spanish Society of Medical Oncology	Prevention and treatment of cancer-related thrombosis	English	Yes	88
5	Cancer Associated Venous Thromboembolic Disease	2021	National Comprehensive Cancer Network	Strategies for the assessment, prevention, and management of cancer-related thrombosis	English	Yes	222

For peer review only - http://bmjopen.bmj.com/site/about/guidelines.xhtml

6	American Society of Hematology 2020 guidelines for management of venous thromboembolism: optimal management of anticoagulation therapy treatment and prophylaxis of venous thromboembolism in patients with cancer	2020	American Society of Hematology	Drug prophylaxis strategies for venous thrombosis	English	Yes	360
7	2019 international clinical practice guidelines for thetreatment and prophylaxis of venous thromboembolism in patients with cancer treatment and prophylaxis of venous thromboembolism in patients with cancer	2019	International Thrombosis and Cancer Initiative	Prevention strategies for cancer-related thrombosis	English	Yes	94
8	Venous thromboembolism in over 16s: reducing the risk of hospital-acquired deep vein thrombosis or pulmonary embolism	2018	National Institute for Health and Care Excellence, UK	Strategies for the prevention and treatment of blood clots	English	Not	330
9	Infusion Therapy Standards of Practice	2021	Infusion Nurses Association	Methods and strategies of clinical infusion therapy	English	Not	471

Note: American Society of Clinical Oncology (ASCO); Spanish Society of Medical Oncology (SEMO); National Comprehensive Cancer Network (NCCN); American Society Hematological(ASO); International Initiative on Thrombosis and Cancer(ITAC-CME); National Institute for Health and Clinical Excellence(NICE); Infusion Nursing(INS).

For peer review only

For peer review only - http://bmjopen.bmj.com/site/about/guidelines.xhtml

Table S2 Specific standardized scores for each AGREE II domain

number	Standardized score by domain (%)						The number of standardized scores ≧ 60%.	recommend level
	range and objective	Participants	Rigour	Clarity	Application	independence		
1	62.96	31.48	31.94	64.81	31.94	44.44	2	B
2	75.93	72.22	63.89	68.52	66.67	75.00	6	A
3	74.07	81.48	77.08	55.56	72.22	69.44	5	B
4	38.89	33.33	30.56	61.11	41.67	61.11	2	B
5	70.37	68.52	66.67	62.96	47.22	61.11	5	B
6	79.63	83.33	84.03	77.78	80.56	72.22	6	A
7	64.81	62.96	64.58	61.11	66.67	52.78	5	B
8	72.22	77.78	72.92	72.22	69.44	72.22	6	A
9	61.11	35.19	65.28	59.26	68.06	63.89	4	B
Mean Score	66.67	60.70	61.88	64.81	60.49	63.58	-	-

For peer review only - http://bmjopen.bmj.com/site/about/guidelines.xhtml

Table S3 Specific standardized scores of AGREE REX in each field

number	AGREE REX Score by Sector (%)			Overall rating
	Clinical applicability	Values and preferences	Implementability	
1	42.59	41.67	50.00	43.83
2	61.11	61.11	66.67	62.35
3	74.07	65.28	77.78	70.99
4	37.04	41.67	61.11	44.44
5	50.00	61.11	72.22	59.88
6	83.33	73.61	77.78	77.78
7	59.26	50.00	63.89	56.17
8	66.67	66.67	77.78	69.14
9	48.15	59.72	52.78	54.32

For peer review only

Table S4. Summary of recommendations included in the guidelines

Items		Source guideline	Recommendation
Risk screening	Thrombosis assessment tool	1、2、3、5、7、8、9	1. The main risk factors assessed included catheterization factors (catheter materials, placement techniques, location and type of catheter, presence of other catheters or catheters, etc.); potential co-disease factors (antiangiogenic therapy, hormone therapy, parenteral nutrition and radiotherapy, tumor and trauma and complex surgery) Patient factors (age, gender, family history or medical history of venous thrombosis, hypercoagulation status and abnormal coagulation gene).<br>2. At present, there is no scale dedicated to CRT risk assessment, and it is recommended to use mature VTE risk assessment scale in clinical practice. Modified Khorana score and Wells scale should be used in CRT risk assessment. Padua scale is recommended for medical patients and IMPED and SAVED scales are recommended for patients with multiple myeloma.
	Timing of thrombo	1、2、3、5、8、9	3. Admission / pre-catheterization / maintenance catheter / symptoms and signs / catheter extraction / discharge / anticoagulant therapy.

For peer review only - http://bmjopen.bmj.com/site/about/guidelines.xhtml

	sis evaluation		
Preventions	Identify as soon as possible	1、3、9	4. Pay close attention to CRT related symptoms and signs such as pain, swelling and skin pruritus on the side of catheterization, and monitor blood routine and coagulation function in time.
	Stay alert.	2、4	5. Correct the wrong operation in time through quality control, conduct regular staff meetings to summarize and feedback on the recent work, and formulate staff briefing warnings to remind other personnel.
	Timely report	1、2、4、8	6. Pay attention to high-risk patients and timely report adverse reactions to prevent them.
	Classified prevention	1、2、3、5、8	7. To evaluate the risk factors and existing symptoms and signs of CRT in patients, and target to prevent and manage them according to different factors and clinical manifestations.<br>8. According to the risk benefit model, individualized drug prevention and physical prevention.
	Multi-disciplinary preventi	1、2、4、5、6、9	9. To construct a venous access management team composed of doctors, nurses, pharmacists, medical technologists, evidence-based medicine experts and managers to improve the scientific and comprehensive of CRT prevention and

For peer review only

	on		treatment.
	Physical prevention	1、2、5、8、9	10.Patients who stay in bed for a long time or have limited activity can massage their lower extremities with intermittent inflatable pressure pump to prevent thrombosis for 1 to 2 weeks. The skin condition of the patients should be evaluated before and after use.<br>11.To carry out daily activities as soon as possible, CVC catheter side should carry out early functional exercise, such as repeatedly clenching , loose, chest breathing and abdominal breathing, etc.<br>12.Replenish the patient with water in time to relieve dehydration.<br>13.For patients at risk of post-thrombosis syndrome, elastic socks are not recommended.
Preventions	Drug prevention	2、4、5、6、8	14.After evaluating the risk of bleeding and thrombosis, unfractionated heparin, low molecular heparin, vitamin K antagonist or direct oral anticoagulant drug can be used for prevention. After taking the drug, the laboratory indexes of the patients should be monitored, INR should be monitored in patients with Warfarin, and the arm circumference of the catheterization side should be measured regularly to ask if the patient has bleeding related manifestations.

For peer review only

For peer review only - http://bmjopen.bmj.com/site/about/guidelines.xhtml

Common catheter-related thrombosis prevention	1、2、3、4、5、6、7、8、9	15. When CVC intubation, the catheter with small diameter, less lumen and less trauma should be selected. If conditions permit, the ratio of the outer diameter of the catheter to the diameter of the catheterized vein should be less than 45%.<br>16. Ultrasonic guided intubation should be used in CVC catheterization, the elbow and other frequently moving positions should be avoided when catheterization, upper elbow with low activity, large diameter veins, fast blood flow rate and more blood flow should be selected. The catheter tip should be the lower third of the superior vena cava or the intersection of chambers<br>17. At present, CVC is not removed except for complications such as any longer indwelling CVC, blockage or dysfunction of CVC catheter, not within normal range, complicated with CRT-related blood flow infection, and so on.<br>18. It is recommended that CVC should be treated with anticoagulant therapy for 1 or 2 weeks before extubation, and then extubation should be anticoagulation at least 3 months after extubation. If CVC functions are normal and decide to continue to use it, even if the anticoagulant period has reached 3 months, it is necessary to continue anticoagulation until the CVC catheter is completely removed.

For peer review only

For peer review only - http://bmjopen.bmj.com/site/about/guidelines.xhtml

Knowledge training	medical staff	2、9	20. Learn CRT diagnosis, prevention related knowledge, master the correct treatment process, participate in bedside rounds and medical record discussions, participate in other healthcare organizations and expert academic exchanges
	Patient	2、3、9	21. Inform patients of disease-related knowledge, such as physical preventive measures, related symptoms and signs, side effects of drugs, emergency management, etc.
	Multi-disciplinary participation	2、3、5、7	22. Multi-disciplinary sharing, active organization of book exchange meetings, regular academic sharing.

For peer review only

For peer review only - http://bmjopen.bmj.com/site/about/guidelines.xhtml

Table S5. Summary of views on preventive measures included in the guidelines

Number	Advice and recommendation	Do not recommend comments
1	In order to reduce the injury caused by CVC, the catheter with small diameter and small number of lumens should be selected as much as possible; ultrasonic guidance should be used to reduce the number of catheterization in CVC catheterization; the elbow joint that often moves should be avoided in CVC catheterization and the upper elbow should be selected; The middle and lower 1/3 of superior vena cava and the intersection of chambers and chambers is the best intubation choice at the tip of CVC; early active after CVC catheterization. The indexes of blood routine and coagulation function and related symptoms and signs were monitored in time after the use of anticoagulant drugs.	Prophylactic use of anticoagulant or thrombolysis drugs is not recommended. Chest enhancement CT and three-dimensional lung imaging are not recommended to screen CRT patients for pulmonary embolism.
2	The prevention of CRT in cancer patients can be based on the combination of drug prevention and mechanical prevention; drug prevention can use heparin (low molecular weight heparin, unfractionated heparin) drugs; CRT high risk patients should be evaluated according to the actual situation after they can decide the time to start anticoagulation; CRT high risk	Mechanical prophylaxis is not recommended as a single measure of VTE prevention; anticoagulant is not

For peer review only - http://bmjopen.bmj.com/site/about/guidelines.xhtml

	patients (major surgery patients and cancer patients) should be given health education.	recommended to improve the survival rate of cancer patients without VTE symptoms.
3	CVC function is normal, the catheter can be retained; all patients should be treated with anticoagulant treatment after removing CVC for at least 3 months; if the anticoagulant time is more than 3 months after CVC catheterization, anticoagulation should continue until the catheter is removed.	For patients with deep venous thrombosis at risk of post-thrombotic syndrom , routine use of elastic socks is not recommended.
4	Risk factors for CRT formation include catheter factors (catheter material, placement techniques, location and type of catheter) and potential co-disease factors (antiangiogenic therapy, hormone therapy, parenteral nutrition and radiotherapy), as well as patient factors (age, sex and thrombosis-related diseases) The first choice of prevention after CVC intubation is to use low molecular heparin anticoagulant therapy for 3-6 months, if CVC has not been removed for more than 3 months, then low molecular heparin is used for anticoagulation indefinitely; direct oral	Except for CVC related infection, unsuitable anticoagulant, poor anticoagulant effect and decision to remove CVC, CVC transfer is not recommended..

For peer review only - http://bmjopen.bmj.com/site/about/guidelines.xhtml

	anticoagulant DOAC is also recommended; if anticoagulant therapy is ineffective or anticoagulant failure, CVC should be removed after 5 or 7 days of anticoagulation.	
5	If the CVC function is normal, you can continue to use it. When CVC is retained, it is recommended that anticoagulant therapy be carried out for CRT formation; if CVC is no longer needed, anticoagulant therapy should be performed 5 or 7 days before CVC removal; if CVC related infection, CRT or worsening symptoms, CVC should be removed when there is anticoagulant contraindication, and the risk of thrombosis should be taken into account, and the anticoagulant treatment should be decided according to the specific situation. In order to prevent CRT, cancer patients should be treated with anticoagulant therapy for at least 3 months, or anticoagulant therapy should be performed throughout the catheterization period; medical personnel should inform patients of the risks and benefits of anticoagulant, and determine the duration of treatment after being known to the patient.	Prophylactic anticoagulant therapy for patients with CVC intubation is not recommended.
6	The use of anticoagulant drugs alone in the treatment of symptomatic CRT in children can minimize complications and do not need to be pulled out of the catheter; for non-functional or no longer used CVC, it is recommended that anticoagulant	Multiple CVC intubation is not recommended; routine use of elastic

For peer review only - http://bmjopen.bmj.com/site/about/guidelines.xhtml

	therapy remove CVC a few days later.	socks is not recommended.
7	The right jugular vein is the first choice for CVC catheterization, in which the intersection of the right atrium and the superior vena cava is the best position for the distal tip of the catheter; for patients with PICC catheterization, the implantation port should be used; for symptomatic CRT, anticoagulant therapy should be performed for at least 3 months as long as CVC is in situ; there is no guideline to compare the anticoagulant effects of low molecular weight heparin, DOAC and vitamin K antagonists If the CVC catheter is unblocked the position of the catheter is normal, no infection occurs, and other symptoms disappear during anticoagulant, CVC can remain in place.	Undifferentiated anticoagulant therapy is not recommended for prevention of CRT formation.
8	When considering whether to use drugs to prevent CRT, it is necessary to analyze the risks and benefits of anticoagulant; LMWH or DOAC can be selected for CRT drug prevention; patients should be educated about VTE risk factors and related symptoms and signs.	Routine thrombosis prevention is not recommended in all patients; thrombosis prevention time is not recommended for less than 12 weeks.
9	The risk of CRT should be assessed before CVC intubation;	It is not

For peer review only - http://bmjopen.bmj.com/site/about/guidelines.xhtml

the appropriate intubation location and catheter type should be selected before catheterization; the selected veins should be observed and measured by ultrasound in advance before PICC intubation, and the catheter with the ratio of catheter diameter to vein diameter less than 45% should be preferred; the tip of CVC should be located at 1/3 of superior vena cava or the junction of vena cava and atrial vein; and the circumference of upper arm should be measured before insertion of PICC to assess the presence of edema and deep venous thrombosis. When CVC is located correctly, functionally normal and has no sign of infection, CVC does not need to be removed even if deep venous thrombosis exists.	recommended that CVC intubation be located in the anterior cubital fossa and prophylactic anticoagulant therapy is not recommended.

For peer review only - http://bmjopen.bmj.com/site/about/guidelines.xhtml

Pubmed Search Strategy

No	Search query
#1	Catheter-related thrombosis[Title/Abstract]
#2	Catheter-related thromboembolism[Title/Abstract]
#3	peripherally inserted central catheter-related thrombosis[Title/Abstract]
#4	venous thromboembolism[Title/Abstract]
#5	venous thrombosis[Title/Abstract]
#6	vein thrombosis[Title/Abstract]
#7	Catheter-related thrombosis[MeSH Terms]
#8	Catheter-related thromboembolism[MeSH Terms]
#9	peripherally inserted central catheter-related thrombosis[MeSH Terms]
#10	venous thromboembolism[MeSH Terms]
#11	venous thrombosis[MeSH Terms]
#12	vein thrombosis[MeSH Terms]
#13	#1 OR #2 OR #3 OR #4 OR #5 OR #6 OR #7 OR #8 OR #9 OR #10 OR #11 OR #12
#14	((clinical practice guideline[Title/Abstract]) OR (guideline[Title/Abstract])) OR (Evidence-Based Practice[Title/Abstract]) Filters: from 2017 - 2022
#15	#13 AND #14

For peer review only

Embase Search Strategy

No	Search query
1	Catheter-related thrombosis.m_titl.
2	Catheter-related thromboembolism.m_titl.
3	peripherally inserted central catheter-related thrombosis.m_titl.
4	venous thromboembolism.m_titl.
5	venous thrombosis.m_titl.
6	vein thrombosis.m_titl.
7	Catheter-related thrombosis.mp.
8	Catheter-related thromboembolism.mp.
9	peripherally inserted central catheter-related thrombosis.mp.
10	venous thromboembolism.mp.
11	venous thrombosis.mp.
12	vein thrombosis.mp.
13	1 or 2 or 3 or 4 or 5 or 6 or 7 or 8 or 9 or 10 or 11 or 12
14	clinical practice guideline.m_titl.
15	guideline.m_titl.
16	Evidence-Based Practice.m_titl.
17	14 or 15 or 16
18	13 and 17
19	limit 18 to yr="2017 - 2022"

For peer review only

Cochrane librarySearch Strategy

No	Search query
1	Catheter-related thrombosis OR Catheter-related thromboembolism OR peripherally inserted central catheter-related thrombosis in Title Abstract Keyword
2	venous thromboembolism OR venous thrombosis OR vein thrombosis in Title Abstract Keyword
3	Catheter-related thrombosis OR Catheter-related thromboembolism OR peripherally inserted central catheter-related thrombosis in Abstract
4	venous thromboembolism OR venous thrombosis OR vein thrombosis in Abstract
5	clinical practice guideline OR guideline OR Evidence-Based Practice in Title Abstract Keyword
6	1 OR 2 OR 3 OR 4 AND 5
7	Custom Range:01/01/2017 to 26/03/2022

Web of Science Search query:

((KP=(Catheter-related thrombosis OR Catheter-related thromboembolism OR peripherally inserted central catheter-related thrombosis OR venous thromboembolism OR venous thrombosis OR vein thrombosis)) OR KP=(Catheter-related thrombosis OR Catheter-related thromboembolism OR peripherally inserted central catheter-related thrombosis OR venous thromboembolism OR venous thrombosis OR vein thrombosis)) AND KP=(clinical practice guideline OR guideline OR Evidence-Based Practice)

publication date: 2017-01-01 to 2022-03-26

Up to date Search query:

(Catheter-related thrombosis OR Catheter-related thromboembolism OR peripherally inserted central catheter-related thrombosis OR venous thromboembolism OR venous thrombosis OR vein thrombosis)) OR (Catheter-related thrombosis OR Catheter-related thromboembolism OR peripherally inserted central catheter-related thrombosis OR venous thromboembolism OR venous thrombosis OR vein thrombosis)) AND (clinical practice guideline OR guideline OR Evidence-Based

For peer review only

Practice)

NICE Search query:

(Catheter-related thrombosis OR Catheter-related thromboembolism OR peripherally inserted central catheter-related thrombosis OR venous thromboembolism OR venous thrombosis OR vein thrombosis OR Catheter-related thrombosis OR Catheter-related thromboembolism OR peripherally inserted central catheter-related thrombosis OR venous thromboembolism OR venous thrombosis OR vein thrombosis) AND (clinical practice guideline OR guideline OR Evidence-Based Practice)

From date 2017/01/01 To date 2023/03/26

Wanfang search query:

((Title or keyword: (catheter-associated thrombosis) or Title or keyword: (catheter-related thromboembolism) or Title or keyword: (Thrombosis through peripheral venous placement of central venous catheter) or Title or keyword: (venous thromboembolism) or All: (venous thrombosis))) AND (Title or keyword: (clinical practice guidelines) or Title or keyword: (guideline) or Title or keyword: (evidence-based practice))). Search time: 2017.1.1~2022.3.26;

VIP: ((((Title or keyword=catheter-related thrombosis OR Title/keyword=catheter-related thromboembolism) OR Title or keyword=central venous catheter thrombosis via peripheral vein) OR (Title/keyword=venous thromboembolism OR title/keyword=venous thrombosis)) AND ((Title or keyword=clinical practice guideline=OR title/keyword=guideline) OR title/keyword=evidence-based practice)) AND (years:[2017 TO 2022])

CNKI:

(Subject: Catheter-associated thrombosis)OR (Subject: Catheter-associated thromboembolism)OR (Subject: Thrombosis of a central venous catheter inserted

For peer review only

through a peripheral vein)OR (Subject: Venous Thromboembolism)OR (Subject: Venous Thrombosis)AND (Subject: Clinical Practice Guidelines)OR (Subject: Guidelines)OR (Subject: Evidence-Based Practice). Publication time: 2017-01-01 to 2022-03-26

Medical Pulse Communication:

(((((Catheter-associated thrombosis) or (catheter-associated thromboembolism) or (peripherally inserted central venous catheter thrombosis) or (venous thrombosis) or (venous thrombosis)AND ((((Clinical Practice Guidelines) or (Guidelines) or (Evidence-Based Practice)

Chinese Biomedical Literature Database:

"Catheter-associated thrombosis" [Title: Smart] OR "Catheter-associated thromboembolism" [Title: Smart] OR "Thrombosis of a central venous catheter through a peripheral vein" [Title: Smart] OR "Venous Thromboembolism" [Title: Smart] OR "Venous Thrombosis" [Title: Smart] AND "Clinical Practice Guidelines" [Title: Smart] AND "Guideline" [Title: Smart] AND "Evidence-Based Practice" [Title: Smart].2017-2022

For peer review only

Revised Standards for Quality Improvement Reporting Excellence (SQUIRE 2.0)
September 15, 2015

Text Section and Item Name	Section or Item Description
**Notes to authors**	. The SQUIRE guidelines provide a framework for reporting new knowledge about how to improve healthcare<br><br>. The SQUIRE guidelines are intended for reports that describe systemlevel work to improve the quality, safety, and value of healthcare, and used methods to establish that observed outcomes were due to the intervention(s).<br><br>. A range of approaches exists for improving healthcare. SQUIRE may be adapted for reporting any of these.<br><br>. Authors should consider every SQUIRE item, but it may be inappropriate or unnecessary to include every SQUIRE element in a particular manuscript.<br><br>. The SQUIRE Glossary contains definitions of many of the key words in SQUIRE.<br><br>. The Explanation and Elaboration document provides specific examples of well-written SQUIRE items, and an in-depth explanation of each item.<br><br>. Please cite SQUIRE when it is used to write a manuscript.
**Title and Abstract**	
**1. Title**	Appraising the quality standard of clinical practice guidelines related to central venous catheter-related thrombosis prevention: a systematic review of reviews(Page 1)
**2. Abstract**	Background: Central venous catheter (CVC)operation is convenient and relatively safe, and it is widely used in clinical practice. Catheter-associated venous thrombosis is one of the common complications after CVC catheterization. Several clinical practice guidelines(CPGs) for Catheter-related thrombosis (CRT) prevention have been published, but their quality is unclear.<br>Objective: To evaluate the quality and analyze the content of clinical practice guidelines regarding central venous catheter-related thrombosis to provide evidence for formulating an evidence-based practice protocol and a risk assessment scale to prevent it.<br>Methods: Clinical practice guidelines regarding central venous catheter-related thrombosis prevention were searched in guidelines websites and databases such as the National Institute for Health and Care Excellence, Up to Date, Pubmed, Web of Science, CNKI, and others. The included guidelines were evaluated using AGREE II.<br>Results: A total of nine guidelines were included, and the quality grade results showed that three were at A-level and six were at B-level. The included

For peer review only

	guidelines mainly recommended the prevention measure of central venous catheter-related thrombosis from three aspects: risk screening, prevention strategies, and knowledge training, with a total of 22 suggestions being recommended.<br>Conclusion: The overall quality of the guidelines is high, but there are few preventive measures for central venous catheter-related thrombosis involved in the guidelines. All preventive measures have not been systematically integrated and evaluated, and no risk assessment scale dedicated to this field has been recommended. Therefore it is urgent to develop an evidence-based practice protocol and a risk assessment scale to prevent it.(Page 2)
**Introduction**	*Why did you start?*
**3. Problem Description**	Catheter-related venous thrombosis is a common complication of central venous catheters in chemotherapy, haemodialysis, nutritional support, and surgical support, and several clinical practice guidelines on CRT prevention have been published, but their quality is unclear. It makes sense to evaluate the quality and analyze the content of the guide. (Page 2)
**4. Available knowledge**	assessment and taking measurements early to prevent patients at high risk of CRT risk are essential to ensure the safety of patients. Clinical Practice guideline is formulated by clinical workers after systematically integrating and evaluating relevant research and clinical experience according to evidence-based concept to a certain common symptom, disease or problem, to provide guiding suggestions for clinical workers and patients, and scientifically promote the application of evidence in clinical practice. At present, several clinical practice guidelines about CRT prevention have been published, but their quality is unclear. Therefore, through the systematic retrieval, screening and reading of clinical practice guidelines related to CRT prevention, and the quality evaluation and content analysis of the guidelines, this review was expected to provide scientific basis for the development of evidence-based practice protocols and risk assessment scales for CRT. (Page 4)

For peer review only

5. Rationale	We searched guideline websites and databases for clinical practice guidelines on central venous catheter-associated thrombosis, and assessed the included guidelines using AGREEII and AGREE REX scale. (Page 6)
6. Specific aims	To evaluate the quality and analyze the content of clinical practice guidelines regarding central venous catheter-related thrombosis to provide evidence for formulating an evidence-based practice protocol and a risk assessment scale to prevent it. (Page 2)
**Methods**	*What did you do?*
7. Context	Central venous catheter (CVC)operation is convenient and relatively safe, and it is widely used in clinical practice. Catheter-associated venous thrombosis is one of the common complications after CVC catheterization. Several clinical practice guidelines(CPGs) for Catheter-related thrombosis (CRT) prevention have been published, but their quality is unclear. (Page 2)
8. Intervention(s)	a. Clinical practice guidelines regarding central venous catheter-related thrombosis prevention were searched in guidelines websites and databases such as National Institute for Health and Care Excellence, Up to date, Pubmed, Web of Science, CNKI, and others. The included guidelines were evaluated using AGREE II and AGREE REX. (Page 4-6).<br>b. Three researchers who systematically trained the use methods of the AGREE II scale and AGREE REX scale were selected to evaluate the guideline to ensure that the researchers had a full understanding and consistent understanding of the items and to reduce the biases of evaluation. If there is a dispute over an item, experts in the relevant field would be consulted to resolve it. (Page 6)
9. Study of the Intervention(s)	The AGREE II scale, a widely used tool for evaluating the quality of the guideline, was used to evaluate the included guidelines. It includes 6 domains, i.e. scope and purpose, rigorism, independence, clarity, application, and independence of writing, with 23 items.<br>the AGREE REX scale was used to evaluate the included guidelines, and three researchers who had been systematically trained in the use of the AGREE REX scale evaluated the guidelines respectively, and the scores of the three researchers in each area of the guidelines were standardized to calculate, and the guidelines were systematically evaluated and content analyzed and summarized based on the evidence-based concept. Referring to the recommendations of the AGREE REX manual, the guidelines define an overall score of >70% as high quality, an overall score of <30% as low quality, and others as moderate quality.(Page 6)
10. Measures	We searched both English and Chinese databases, including Pubmed, Embase, Cochrane Library, Web of Science, CNKI, Wanfang, VIP, and the Chinese Biomedical Literature Database from January 1st, 2017 to March 26th, 2022. In addition, the relevant websites of the guideline, including to date, National Institute for Health and Care Excellence (NICE), and Medline, were searched. The manual search of relevant studies and bibliography was also adopted.<br>Three researchers who had systematically trained the use of the AGREE II and AGREE REX scale were selected to conduct relevant research on the guidelines using the scale and evaluate their quality, including a total of 6 areas, including scope and purpose, study rigor, independence, clarity of discussion, application, and independence of writing, a total of 23 items, and SPSS22.0 was used to count the scores of two investigators in each field of the guidelines and calculate the relevant results.(Page 6-7)
11. Analysis	SPSS22.0 was used to count the scores of the two researchers in each field of the guideline, and the Intraclass Correlation Coefficient (ICC) of the data was calculated. According to the results, the consistency of the two researchers' evaluation of the guideline was tested. ICC < 0.4 indicated poor consistency, 0.40 ≤ 0.59 indicated that the consistency is general, 0.60 ≤ 0.74 indicated good consistency, and > 0.74 indicated that the consistency is very good. (Page 7)

For peer review only

**12. Ethical Considerations**	Competing interests： The authors declare that they have no conflicts of interest.<br>Ethical considerations：Not involved
**Results**	*What didyoufind?*
**13. Results**	a. A total of 2092 articles were retrieved, and 571 articles were removed by software and manual deletion. After read the title and abstract, 1142 articles were excluded. 379 articles were screened according to inclusion and exclusion criteria. 267 articles that did not meet the theme of the article were excluded, and 5 related guidelines were traced through the snowball strategy, and 117 articles were finally selected. Among them, 11 articles were not obtained, and a total of 106 complete articles were obtained. Full-text screened excluded 18 non-evidence-based guidelines issued in individual form, 6 guidelines that had been updated, and 73 guidelines that did not include CRT treatment, prevention, and management. Finally, 9 literatures were selected, including 2 Chinese literatures and 7 English literatures. (Page 7)<br>b. The nine guidelines were from the American Society of Clinical Oncology, the International Initiative on Thrombosis and Cancer, the Chinese Medical Association, the Spanish Medical Oncology Society, the Infusion Nursing Association, and other international authoritative medical associations. Only one of them is a guideline targeted for CRT risk assessment, prevention, and intervention18. Five guidelines were specifically for venous thrombosis prevention in cancer patients, and the content related to CRT was only part of it. Two of which are guidelines for venous thrombosis of ordinary patients. CRT patients are part of the content, and only one guideline is targeted for clinical infusion therapy. CRT is part of the treatment.(Page 8)<br>c. After scoring the nine guidelines with the AGREEII tool, it was found that the recommended A-level guidelines were three. The recommended B-level guidelines are six. The average score in all areas was greater than 60%. The ICC of the scores of the three reviewers for each domain of each guideline was greater than 0.8, indicating that the agreement among the reviewers was good. The details are shown in Table S3.<br>After evaluating the nine guidelines according to the AGREE REX scale, the overall scores of guidelines 3 and 6were > 70%, which was of high quality, and the overall scores of the other guidelines were ≥ 30%, which was of moderate quality. The details are shown in Table S4. (Page 8-9)<br>d. We summarised the similarities and differences in the guidelines for thromboprophylaxis associated with central venous catheters in table S5 and table S6.(Page 9-10)
**Discussion**	*What does it mean?*
**14. Summary**	a. Key findings：The overall quality of the nine guidelines included is good, and the corresponding advice and suggestions about CRT prevention are provided, but the guidelines still need to be improved in terms of rigorism and independence of writing, participants and applications. At present, there is still a lack of high-quality clinical practice guidelines for CRT prevention, and there is also a lack of dedicated CRT risk assessment tool. (Page 8-9)<br>b. Particular strengths of the project：NA

For peer review only

**15. Interpretation**	a. Nature of the association between the intervention(s) and the outcomes: NA<br>b. Comparison of results with findings from other publications: NA<br>c. Impact of the project on people and systems : NA<br>d. By evaluating the content and quality of these guidelines, it is found that there is currently a lack of high-quality clinical practice guidelines dedicated to CRT prevention at home and abroad, and there is currently a lack of risk assessment scales dedicated to CRT in clinical practice. We believe that it is urgent to develop clinical practice guidelines and risk assessment scales related to CRT prevention based on evidence-based concepts, so as to involve patients in research based on clinical status, reduce the incidence of CRT, and improve the level of CRT detection, management and prevention.(Page 15-16)<br>e. Costs and strategic trade-offs, including opportunity costs: NA
**16. Limitations**	The evidence in the guidelines included in this study is mainly from developed countries and some developing countries, and other countries need to be cautious when interpreting the results of this study
**17. Conclusions**	The overall quality of the nine guidelines included is good, and the corresponding advice and suggestions about CRT prevention are provided, but the guidelines still need to be improved in terms of rigorism and independence of writing, participants and applications. At present, there is still a lack of high-quality clinical practice guidelines for CRT prevention, and there is also a lack of dedicated CRT risk assessment tool. Therefore, there is an urgent need to develop evidence-based CRT prevention related clinical practice guidelines and risk assessment tools based on the clinical situation to enable patients to participate in the management of CRT, thereafter to to better manage the CRT.(Page 16)
**Other information**	
**18. Funding**	This work was supported by the Sichuan Science and Technology Program (Grant 2022NSFSC1290), and West China Nursing Discipline Development Special Fund Project, Sichuan University (Grant HXHL21011). (Page 17)

For peer review only

Table 2. Glossary of key terms used in SQUIRE 2.0. This Glossary provides the intended meaning of selected words and phrases as they are used in the SQUIRE 2.0 Guidelines. They may, and often do, have different meanings in other disciplines, situations, and settings.

Assumptions

Reasons for choosing the activities and tools used to bring about changes in healthcare services at the system level.

Context

Physical and sociocultural makeup of the local environment (for example, external environmental factors, organizational dynamics, collaboration, resources, leadership, and the like), and the interpretation of these factors ("sense-making") by the healthcare delivery professionals, patients, and caregivers that can affect the effectiveness and generalizability of intervention(s).

Ethical aspects

The value of system-level initiatives relative to their potential for harm, burden, and cost to the stakeholders. Potential harms particularly associated with efforts to improve the quality, safety, and value of healthcare services include opportunity costs, invasion of privacy, and staff distress resulting from disclosure of poor performance.

Generalizability

The likelihood that the intervention(s) in a particular report would produce similar results in other settings, situations, or environments (also referred to as external validity).

Healthcare improvement

Any systematic effort intended to raise the quality, safety, and value of healthcare services, usually done at the system level. We encourage the use of this phrase rather than "quality improvement," which often refers to more narrowly defined approaches.

Inferences

The meaning of findings or data, as interpreted by the stakeholders in healthcare services – improvers, healthcare delivery professionals, and/or patients and families

Initiative

A broad term that can refer to organization-wide programs, narrowly focused projects, or the details of specific interventions (for example, planning, execution, and assessment)

Internal validity

Demonstrable, credible evidence for efficacy (meaningful impact or change) resulting from introduction of a specific intervention into a particular healthcare system.

Intervention(s)

The specific activities and tools introduced into a healthcare system with the aim of changing its performance for the better. Complete description of an intervention includes its inputs, internal activities, and outputs (in the form of a logic model, for example), and the mechanism(s) by which these components are expected to produce changes in a system's performance.

Opportunity costs

For peer review only

Loss of the ability to perform other tasks or meet other responsibilities resulting from the diversion of resources needed to introduce, test, or sustain a particular improvement initiative

Problem

Meaningful disruption, failure, inadequacy, distress, confusion or other dysfunction in a healthcare service delivery systemthat adversely affects patients, staff, or the system as a whole, or that prevents care from reaching its full potential

Process

The routines and other activities through which healthcare services are delivered

Rationale

Explanation of why particular intervention(s)were chosen and why it was expected to work, be sustainable, and be replicable elsewhere.

Systems

The interrelated structures, people, processes, and activities that together create healthcare services for and with individual patients and populations. For example, systems exist from the personal self-care system of a patient, to the individual provider-patient dyad system, to the microsystem, to the macrosystem, and all the way to the market/social/insurance system. These levels are nested within each other.

Theory or theories

Any "reason-giving" account that asserts causal relationships between variables (causal theory) or that makes sense of an otherwise obscure processor situation (explanatory theory). Theories come in many forms, and serve different purposes in the phases ofimprovementwork. It is important to be explicit and well-founded about any informal and formal theory (or theories) that are used.

For peer review only
